# The Identification of Potent, Selective, and Brain Penetrant PI5P4Kγ Inhibitors as In Vivo-Ready Tool Molecules

**DOI:** 10.1021/acs.jmedchem.2c01693

**Published:** 2022-12-14

**Authors:** Timothy P. C. Rooney, Gregory G. Aldred, Helen K. Boffey, Henriëtte M. G. Willems, Simon Edwards, Stephen J. Chawner, Duncan E. Scott, Christopher Green, David Winpenny, John Skidmore, Jonathan H. Clarke, Stephen P. Andrews

**Affiliations:** The ALBORADA Drug Discovery Institute, University of Cambridge, Island Research Building, Cambridge Biomedical Campus, Hills Road, Cambridge CB2 0AH, United Kingdom

## Abstract

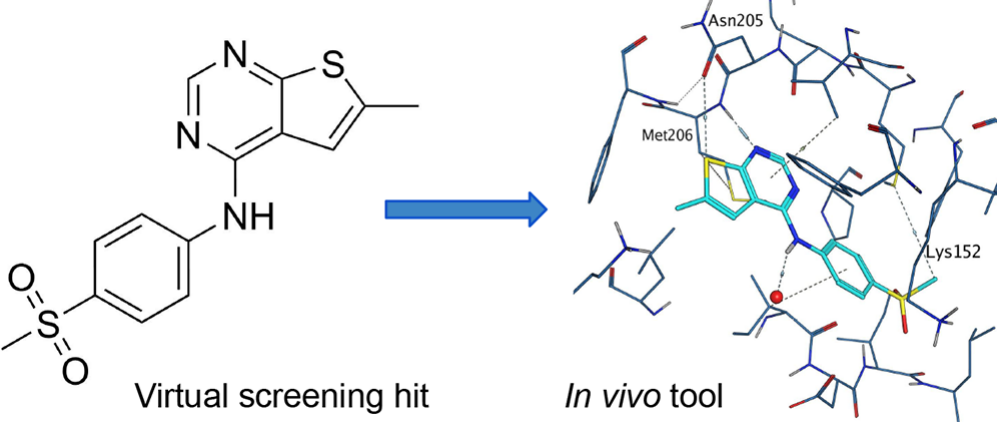

Owing to their central role in regulating cell signaling pathways, the phosphatidylinositol 5-phosphate 4-kinases (PI5P4Ks) are attractive therapeutic targets in diseases such as cancer, neurodegeneration, and immunological disorders. Until now, tool molecules for these kinases have been either limited in potency or isoform selectivity, which has hampered further investigation of biology and drug development. Herein we describe the virtual screening workflow which identified a series of thienylpyrimidines as PI5P4Kγ-selective inhibitors, as well as the medicinal chemistry optimization of this chemotype, to provide potent and selective tool molecules for further use. In vivo pharmacokinetics data are presented for exemplar tool molecules, along with an X-ray structure for ARUK2001607 (**15**) in complex with PI5P4Kγ, along with its selectivity data against >150 kinases and a Cerep safety panel.

## Introduction

Phospholipids mediate many cell signaling events in mammalian cells, and the cycling of phosphoinositides (PIs) plays a central role in these processes.^1,2^ The canonical PI cycle generates the bisphosphorylated signaling molecule phosphatidylinositol bisphosphate (PI(4,5)*P*_2_), a precursor (through PI-specific phospholipase C activity) of inositol trisphosphate (IP_3_) and diacylglycerol (DAG). The latter two molecules are able to initiate cellular signaling cascades through calcium release and activation of protein kinases. Further discoveries have expanded the interconvertible network of PIs to include seven possible derivatives by phosphorylation of sites 3, 4, and 5 of the 6-carbon ring of the inositol headgroup. A broad range of lipid phosphatases and kinases orchestrate the generation of PIs in mammalian cells, which can be cell-type-, pathway-, and subcellular location-specific.

Recent interest has centered around the involvement of specific PIs in the regulation of autophagic pathways.^3,4^ The lipid kinases involved as components of these control mechanisms have thus been identified as potential therapeutic targets in both cancer and neurodegenerative disease.^5−10^ Canonical macroautophagy is dependent on the generation of PI3P by the core complex containing the class III PI 3-kinase (PI3KC3 or yeast orthologue VPS34) and subsequent recruitment of WIPI proteins.^3,10^ Alternatively, PI3P-independent macroautophagy may involve PI5P in the recruitment of WIPI2 to the emerging autophagosome,^11^ suggesting a role in autophagy initiation for enzymes that modulate this inositol lipid. Phosphatidylinositol 5-phosphate 4-kinases (PI5P4Ks) reduce the cellular PI5P pool, using this substrate to generate PI(4,5)*P*_2_,^12^ which has also been shown to be required for successful autophagosome-lysosome fusion.^13,14^ Pharmacological inhibition of PI5P4K activity has been shown to have potential in the treatment of disease, for example, in reducing mutant protein levels in Huntington disease models and reducing proliferation in leukemic cell lines.^15−17^

Mammals express three isoforms (α, β, and γ) of PI5P4Ks which have differing tissue specificity and subcellular localization.^18,19^ Sequence identity between the three isoforms is high, allowing heterodimerization in cells. Variation in catalytic site and putative G-loop sequences may account for the large differences in intrinsic in vitro kinase activity between the isoforms (PI5P4Kγ being 2000-fold less active than PI5P4Kα),^19,20^ although these differences may be attenuated in vivo.^21^ Pan-specific PI5P4K inhibitors have recently been identified that have therapeutic effect in oncology settings.^15,22^ The complexity of the different cellular roles of the PI5P4Ks, and the relevance of these roles to different diseases, suggests that the development of specific tool inhibitors to the different isoforms will enable mechanistic research into these potentially diverse functions.^16,18,23^ In particular, the generation of a potent, specific inhibitor to PI5P4Kγ will enable further elucidation of the role of this kinase in a variety of diseases and validate its potential as a therapeutic target.^9,16^ We have previously reported the development of PI5P4Kγ-specific inhibitors,^18,23^ but these were limited by both modest potency and compromised drug-like properties. Herein we describe the identification of superior tool molecules with low nM PI5P4Kγ inhibition concentrations, good selectivity against other kinases, and optimized drug-like properties, including extended in vivo half-lives and brain penetration. We anticipate these tools will be useful for elucidating the role of PI5P4Kγ in a range of biological pathways.

## Results and Discussion

**Figure 1:**
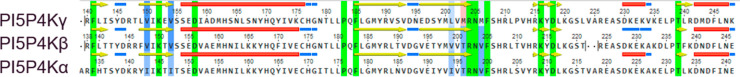
Sequence alignment of the human α, β, and γ sequences. Active site residues are highlighted: green = identical active site residue, blue-to-red scale = similar to dissimilar active site residues (MOE similarity scale). Secondary structure elements are shown as horizontal bars or arrows: red = α helix, yellow = β-sheet, blue = turn.

We have previously described approaches to use known PI5P4K ligands to generate tool molecules for these kinase targets.^23^ Herein we describe complementary approaches to identify novel ligands through virtual screening (VS). For the VS approach, our initial aim was to purchase around 1000 compounds for biological screening to give a reasonable chance to find several hit chemotypes.^24^ We were interested in finding ligands for all three subtypes of PI5P4K. The α, β, and γ subtypes of PI5P4K have almost identical active site residues (Figure 1), with the most significant change being a methionine in PI5P4Kγ (M203) replacing a threonine in PI5P4Kβ (T201) and PI5P4Kα (T196). Owing to the structural similarity, a single virtual screening approach was employed for all three subtypes.

GOLD library screening (fast docking) was used to screen a 31000-member, kinase-focused compound library, commercially available from BioAscent. Most of this library was found to dock to the assumed ATP-site of the PI5P4Kα crystal structure (PDB 2YBX). At the time the docking was carried out, several kinase inhibitors with PI5P4K affinity had been reported.^25,26^ We tested three of these in-house: KW-2449, sunitinib, and palbociclib. All three were found to be inactive (pIC_50_ < 5) in our PI5P4Kα and PI5P4Kγ+ ADP-Glo assays (described below). In the absence of active PI5P4K ligands for benchmarking the docking protocol, the best solutions from the docked set were selected with a MOE pharmacophore that represented key AMP binding features (1.4 Å acceptor projection spheres on Val199NH, Lys209NZ; 1.4 Å donor projection spheres on Arg197O and Asn198OD1, 2 out of 4 required; 1.4 Å exclusion spheres on all other active site residues).

**Figure 2:**
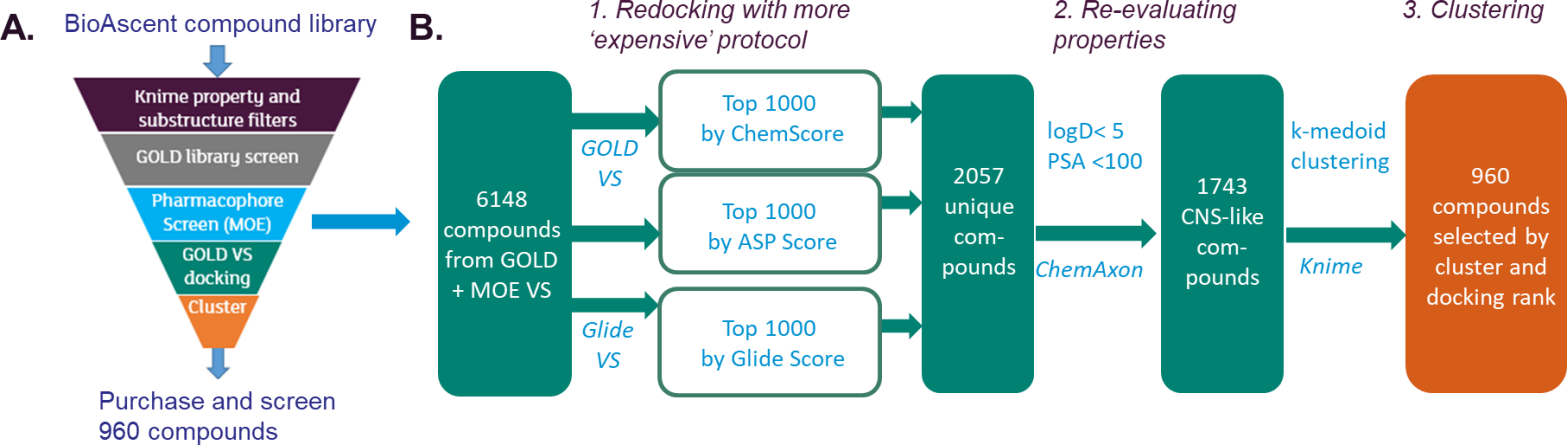
Virtual screening workflow. (A) Overview of workflow. (B) Detail of the redocking step.

The 6148 compounds that passed the pharmacophore filter were redocked with both GOLD and Glide and scored with three different scoring functions. The top 1000 solutions for each were selected to give 2057 unique compounds. A diverse set of 960 compounds was purchased from this subset based on docking scores and clustering and screened in functional assays to measure kinase inhibition using an ADP-Glo reporter against PI5P4Kα and an engineered form of PI5P4Kγ. The overall virtual screening process is summarized in Figure 2.

PI5P4Kγ-WT (wild-type) has particularly low enzymatic activity, which is not trivial to measure and hampers screening for inhibitors. As described previously,^23^ it is possible to use a “PI5P4Kγ+” construct which has been engineered to have a higher functional activity. It is important to note that, compared with PI5P4Kγ-WT, the PI5P4Kγ+ construct contains a number of PI5P4Kα-like mutations (insertion of three amino acids (QAR) at 139 plus an additional 11 amino acid mutations: S132L, E133P, S134N, E135D, G136S, D141G, G142A, E156T, N198G, E199G, and D200E); while this construct is useful for screening large numbers of compounds and for routine screening of chemotypes with well-understood PI5P4K isoform selectivity, there is a possibility of being misled if appropriate additional steps are not taken to ensure that the affinity tracks that of the wild type enzyme. In this work, a cell-based thermal stabilization (InCELL Pulse) assay was used with PI5P4Kγ-WT to confirm genuine PI5P4Kγ activity for compounds of interest, moreover, representative compounds were validated as PI5P4Kγ-WT binders using biophysical methods (see Supporting Information). From the purchased VS set described above, compounds **1**–**5** were found to have pIC_50_ values >5 in the PI5P4Kγ+ assay which equates to a hit rate of 0.5% (Table 1). A further 9 hits were identified for PI5P4Kα and their development will be disclosed elsewhere.

**Table 1:**
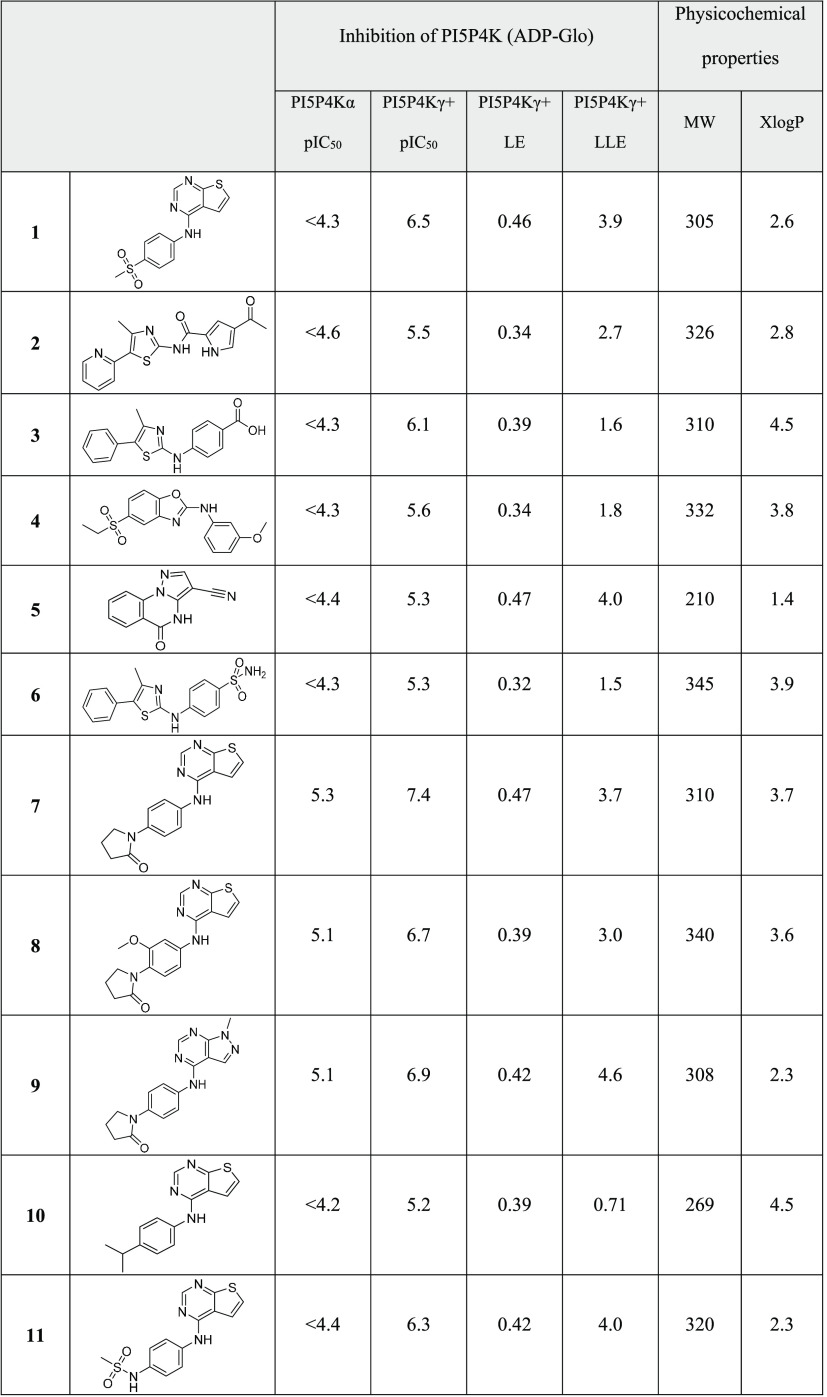
Potency Data for the Virtual Screening Hits and Selected Purchased Analogues

The hits were followed up with analogue purchases, with compounds being selected by substructure, Tanimoto similarity, or shape similarity. The 6 analogues identified for **4**, and 37 analogues of **5** were found to be inactive (data not shown), so these chemotypes were not pursued further. Although several analogues of the **2**/**3** cluster showed activity, they were all less potent than the initial VS hit, with **6** being the most active, so this series was also not pursued further. Compound **1** was a promising hit with submicromolar PI5P4Kγ+ inhibition, high ligand efficiency^27^ (LE = 0.46), high lipophilic ligand efficiency^28^ (LLE = 3.9), and no measurable inhibition of PI5P4Kα. The most active analogues initially purchased for **1** also showed some high LEs and LLEs (**7**–**11**), with compound **7** reaching a pIC_50_ of 7.4 (Table 1). This thienylpyrimidine series was selected for further exploration.

Additional SAR of the series was scoped through synthesis, initially by making small variations to the thienylpyrimidine heterocycle (Table 2) or sulfone (Table 3). Of the heterocycles explored, the original thienylpyrimidine has one of the most favorable LE-LLE combinations, reflecting high levels of inhibition relative to both its MW and XlogP. While replacement of the N atoms in compound **1** by CH did not lead to appreciable loss of activity in the resulting compounds **12** or **13**, it was preferable to maintain the lower XlogP and higher heteroatom count of **1** for drug-like properties. Adding a methyl group at the 2-position of the thienylpyrimidine led to a reduction in activity (**14**), whereas a methyl group at the 6-position was beneficial to activity, LLE and LE (**15**). The *N*-methyl pyrazolopyrimidine **16** also showed good LE and LLE, whereas heterocycles **17**–**21** were less active.

**Table 2:**
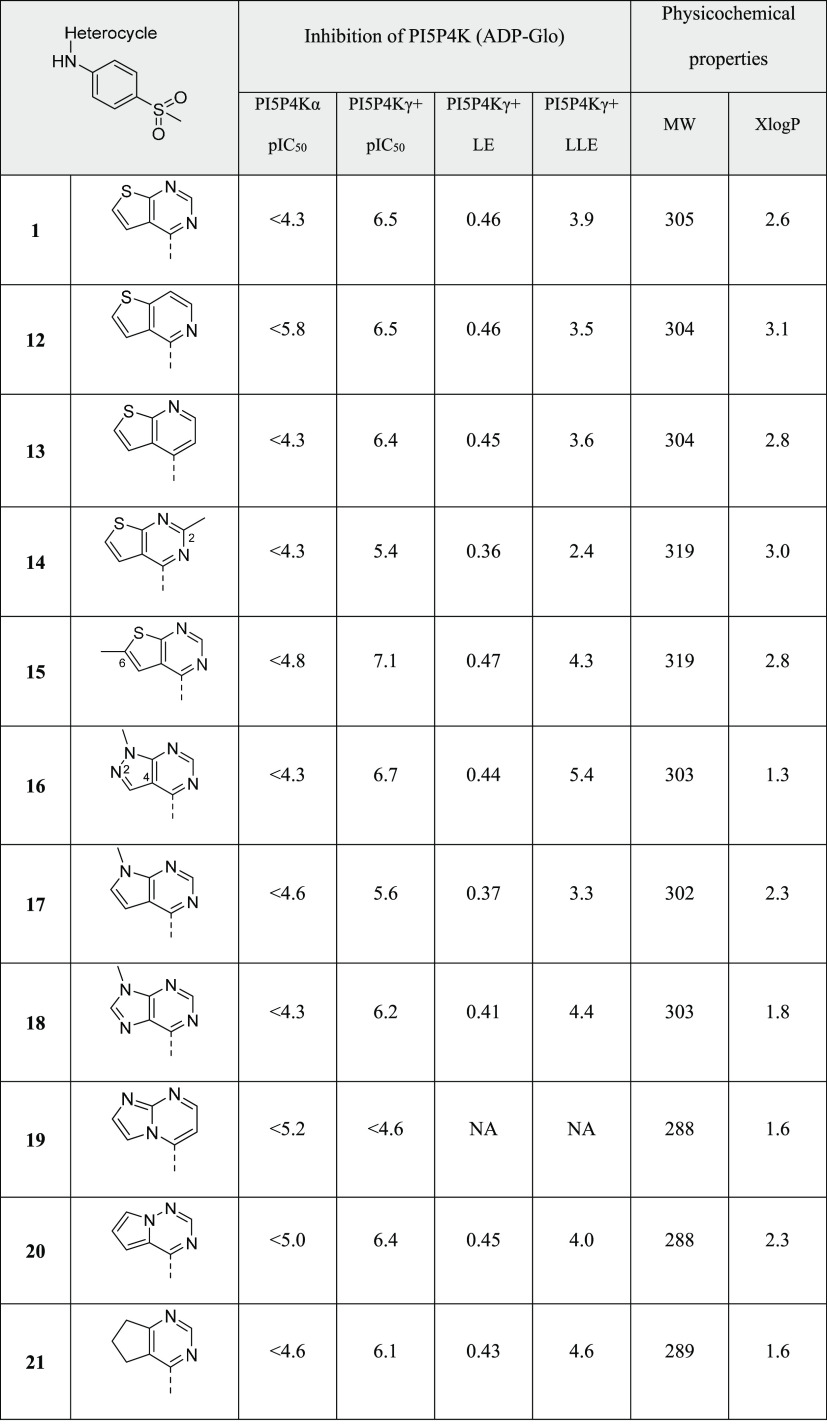
SAR Exploration of the Thienylpyrimidine Moiety of Virtual Screening Hit **1**

An extensive survey of polar groups such as sulfones, sulfonamides, and amides was carried out at position R^1^ as SAR of commercial analogues such as **1**, **7**, and **11** vs **10** showed a preference for polarity at this position. Extension of the methyl group of sulfone **1** with larger aliphatic groups gave a boost in PI5P4Kγ+ inhibition, as exemplified by **22**–**25** (Table 3). Addition of a methylene spacer was not tolerated (**26**), and replacement of the sulfonyl group with a range of lipophilic substituents diminished activity (**10**, **27**–**29**). Of all of the sulfones investigated, **1** retained the highest LE and LLE.

**Table 3:**
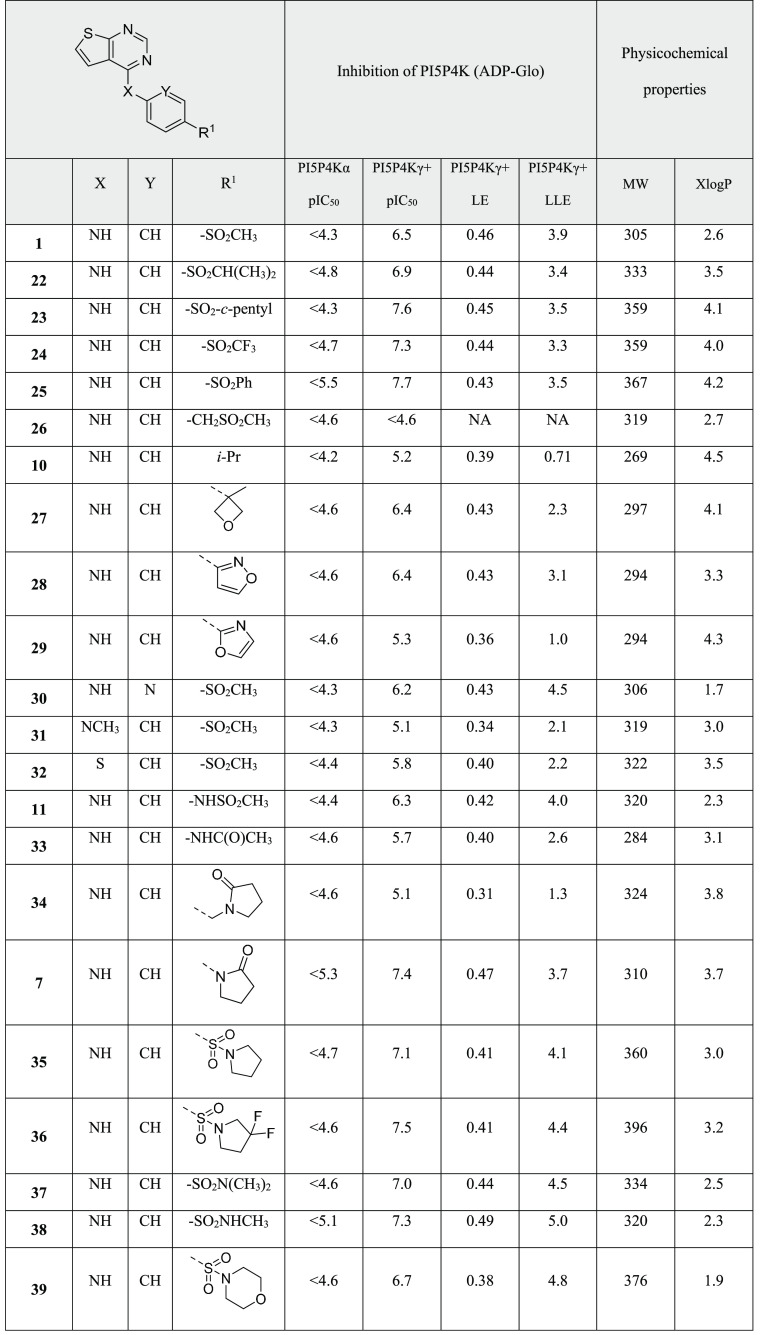
SAR for Varying Positions R^1^, X, and Y

It was possible to substitute carbon for nitrogen at position Y, reducing XlogP and increasing LLE without significant loss of activity (**30**), but the hydrogen bond donor could not be removed at the linker position X without a more significant loss of activity, exemplified by *N*-methylation or NH replacement by S (**31** and **32**, respectively). Sulfonamide **11** has been described above and, indeed, position R^1^ tolerated a range of sulfonamides and amides at this position, with a variety of sizes and configurations. Simple primary amide **33** and methylene-extended **34** had lower activity but, in particular, lactam **7** and sulfonamides **35**–**38** showed high levels of PI5P4Kγ+ inhibition and some of the highest LEs and LLEs identified within the chemical series. Larger sulfonamides such as **39** were tolerated but did not offer an advantage.

Position R^2^ on the thienylpyrimidine ring was next explored for SAR (Table 4). A range of polar and apolar small groups were tested (CH_3_, Cl, CN, CF_3_; Table 4). Of these, chloro showed the lowest inhibition (**40**), whereas methyl (**15**), cyano (**41**), and trifluoromethyl (**42**) showed high levels of inhibition while maintaining good physicochemical properties. Large, apolar phenyl was tolerated (**43**) and was shown to have high inhibition at the cost of lower LLE, whereas polar dimethylaminomethyl (**44**) showed diminished activity. Of these, a methyl group at position R^2^ gave the highest LE (**15**); combining this with two of the R^1^ groups which had given high potency led to compounds **45** and **46**, the latter being one of the most active compounds identified but at the cost of high XlogP.

**Table 4:**
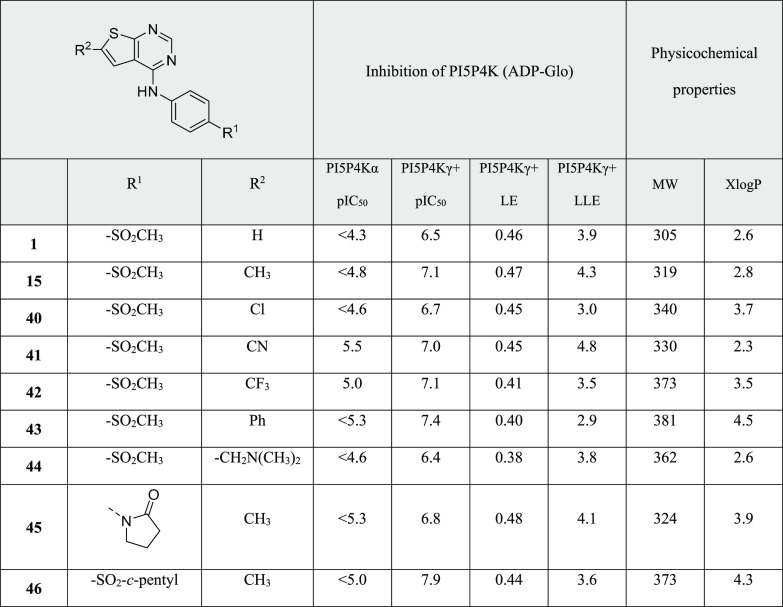
Exploration of Substituents at Position R^2^

A representative compound from the series was submitted for further selectivity profiling: compound **15** was selected as an exemplar of the series with good activity, LLE and LE. Activity was determined against a diverse panel of 140 protein kinases at 10 μM. Of the kinases tested, only AURKB and CLK2 showed <50% residual activity (Table 5). The PI5P 4-kinases are lipid kinases, so as many lipid kinases were screened as possible: 23 commercial lipid kinase assays were identified, including a binding assay for PI5P4Kγ, which returned a *K*_D_ of 7.1 nM. Only one other lipid kinase returned significant levels of activity, PIP5K1C, returning a *K*_D_ of 230 nM. In a Cerep safety panel of 24 diverse cellular and nuclear receptors, 10 enzymes and uptake receptors, plus 6 ion channels, only one hit was identified with >50% inhibition (Table 5). Further details from these screens are provided in Supporting Information, Tables S3–S10.

**Table 5:** Compound **15** Selectivity vs Protein Kinases, Lipid Kinases, and a Cerep Safety Panel

panel screened	results
140 protein kinases	Compound screened at 10 μM. Two hits with <50% residual activity: AURKB (31%), CLK2 (37%)

23 lipid kinases	2 hits: PI5P4Kγ *K*_D_ = 7.1 nM, PIP5K1C K_D_ = 230 nM

Cerep safety panel (24 cellular and nuclear receptors, 10 enzymes and uptake receptors, 6 ion channels)	Compound screened at 10 μM. One hit >50% inhibition compared with control: dopamine uptake (59%)

Compounds that were identified with PI5P4Kγ+ inhibition pIC_50_ ≥ 6.5 were progressed for screening in further assays (Table 6). In particular, these compounds were evaluated for their ability to bind to PI5P4Kγ-WT in cells, for inhibition of PI5P4Kβ, and for in vitro ADMET properties consistent with further development as an in vivo tool molecule. The compounds presented in Table 6 cover a wide range of ADMET properties. Passive permeabilities range from moderate (6.2 × 10^–6^ cm/s) to high (25.9 × 10^–6^ cm/s) in MDCK-MDR1 cells and efflux ratios (ERs) in these cells, which overexpress Pgp, range from low (1.3) to high (9.0). Aqueous solubility at pH 7.4 ranges from very low (<1 μM) to high (>100 μM), and half-lives in mouse liver microsomes (MLM) range from low (1.2 min) to high (214 min).

**Table 6:**
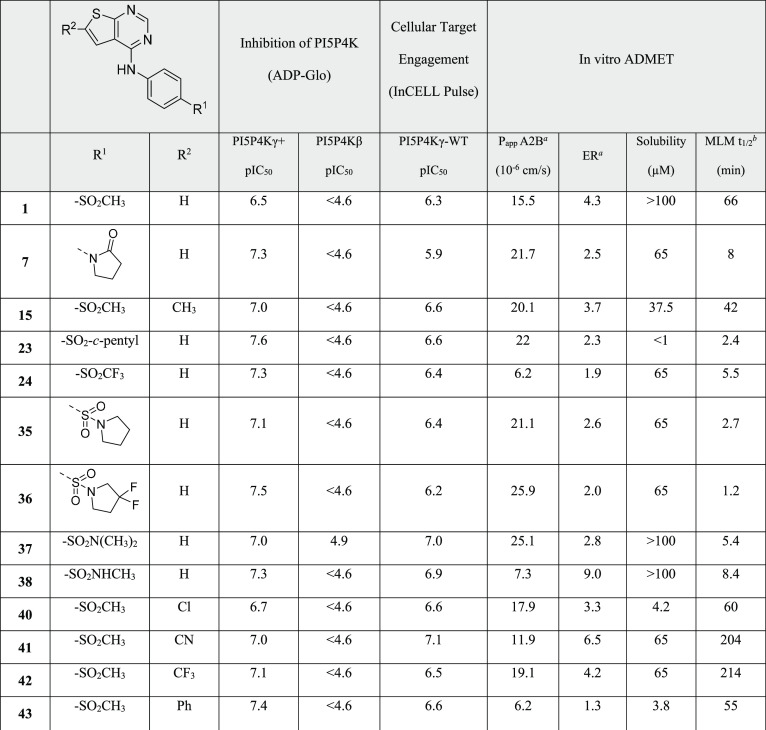
Activity against PI5P4Kβ, Cellular Target Engagement, and in Vitro ADMET Data

a Bi-directional permeability determined in MDCK-MDR1 cells.

b Mouse liver microsome stability.

The compounds presented in Table 6 were tested in a cellular target engagement assay with PI5P4Kγ-WT. The series showed clear engagement of PI5P4Kγ-WT in cells and compounds with optimal ADMET properties showed little drop-off from the PI5P4Kγ+ enzyme assay. Furthermore, a selection of ligands with a range of PI5P4Kγ+ ADP-Glo pIC_50_ values was profiled for binding at the PI5P4K-WT protein using MST and DSF. The rank ordering of PI5P4Kγ+ ADP-Glo pIC_50_s was broadly consistent, with p*K*_D_s measured by MST across an IC_50_ range of more than 2 orders of magnitude (Supporting Information, Table S1), and the ADP-Glo pIC_50_s were consistent with p*K*_D_s measured for 3 diverse compounds by DSF (Supporting Information, Figure S2). In general, the compounds did not exhibit PI5P4Kβ inhibition. ChEMBL was searched for known compounds which are most similar to examples from this series and for the most similar known kinase inhibitors to this chemotype (see Supporting Information).

**Figure 3:**
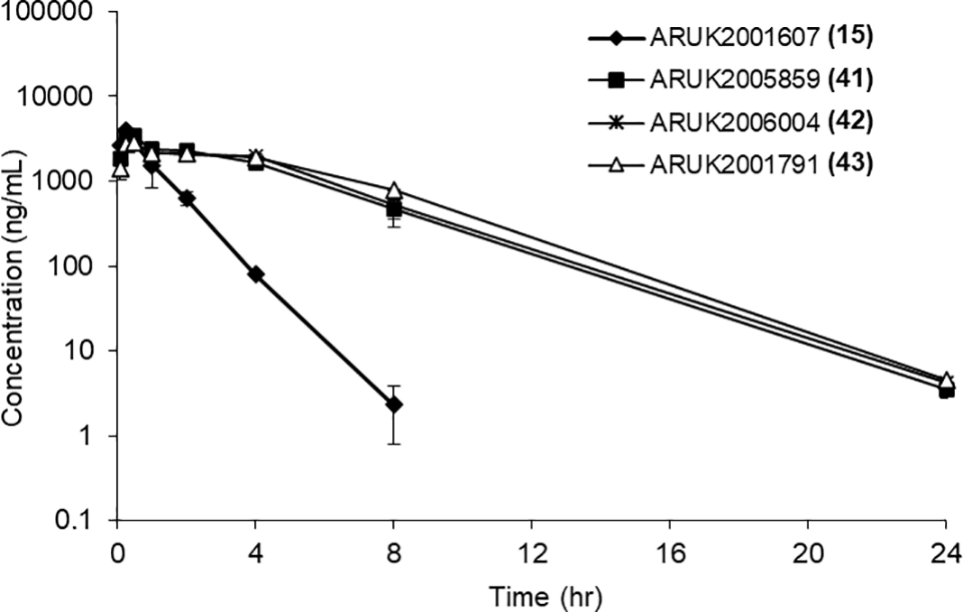
In vivo mouse pharmacokinetics plasma concentration–time profiles for selected compounds (5 mg/kg; ip administration).

Of particular interest for further progression to in vivo pharmacokinetics studies were **15** and **41**–**43**. These compounds showed good potencies at PI5P4Kγ (WT and γ+), were selective vs PI5P4Kα and β, showed moderate to good permeability and efflux in MDCK cells, and moderate to good stability in MLMs. These compounds were administered by cassette intraperitoneally in mice at 5 mg/kg and evaluated for brain and plasma exposure. Brain protein binding (BPB) and plasma protein binding (PPB) were determined in vitro to enable unbound partition coefficients (*K*_p,uu_s) to be determined (Table 7 and Figure 3).

**Table 7:**
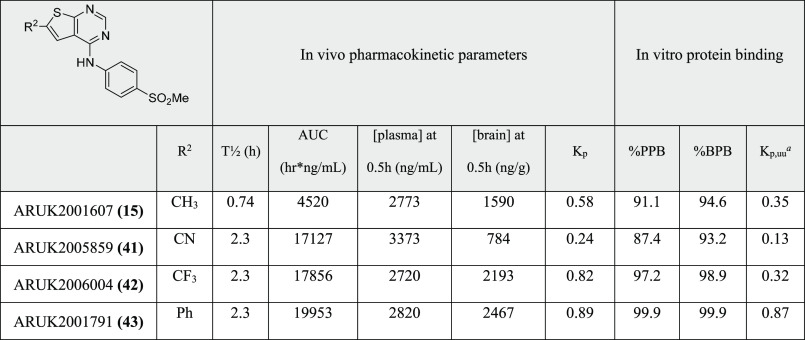
In Vivo Mouse Pharmacokinetics Parameters for Selected Compounds (5 mg/kg; ip Administration)

a Unbound partition coefficient, *K*_pu,u_, determined as a ratio of free brain:free plasma concentrations at 0.5 h; free concentrations for each compartment were calculated from total concentration in that compartment and the level of binding.

Compound **15** had the shortest microsomal half-life of this set, and this tracked over to the shortest in vivo half-life. Lipophilic **43** showed very high levels of plasma and brain protein binding which may, in part, account for its longer half-life compared to **15** (the two compounds have similar microsomal half-lives). Compounds **41** and **42** both showed long microsomal and in vivo half-lives. All four compounds showed good total brain exposure, with moderate–good *K*_p,uu_. Both **15** and **41** showed relatively low PPB and BPB for the series but **41** showed higher efflux in MDCKs than **15**, so is likely a Pgp substrate, hence the lower *K*_p_.

**Figure 4:**
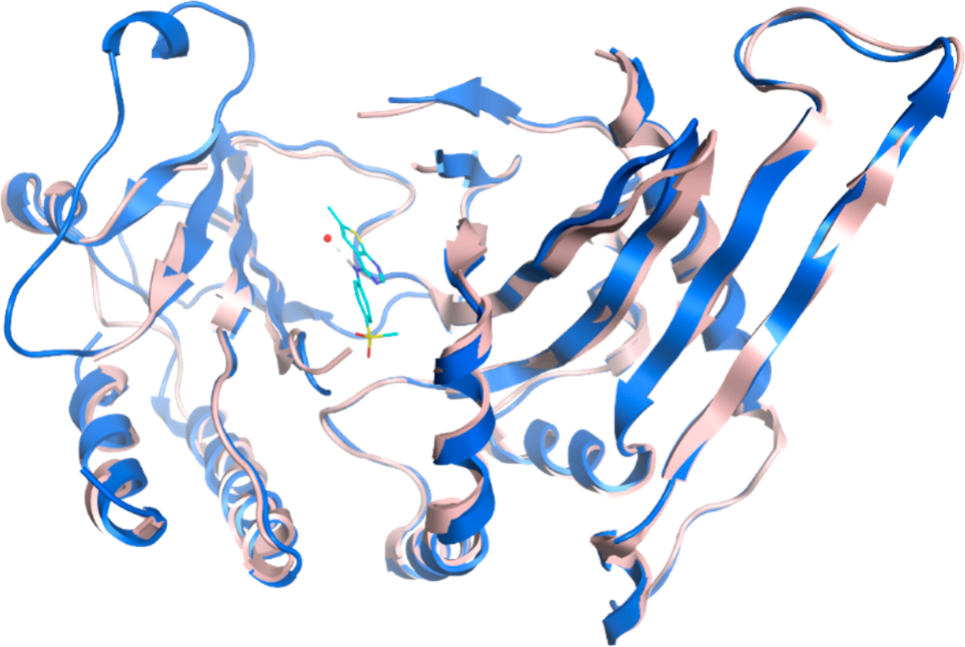
Crystal structure comparison: apo PI5P4Kγ (2GK9; pink) and PI5P4Kγ bound to compound **15** (8BQ4; blue). Only chain A of each structure is shown.

To solve structures of PI5P4Kγ as cocomplexes with examples from this chemical series, a truncated version of human PI5P4Kγ comprising residues His32 to Ala421 was cloned into a bacterial expression vector. This truncation had previously been shown to be suitable for crystallization,^23^ and here this construct, with the region between residues 309–331 deleted, was successfully cocrystallized with **15** at a 2.4 Å resolution (Figure 4, PDB 8BQ4).

**Figure 5:**
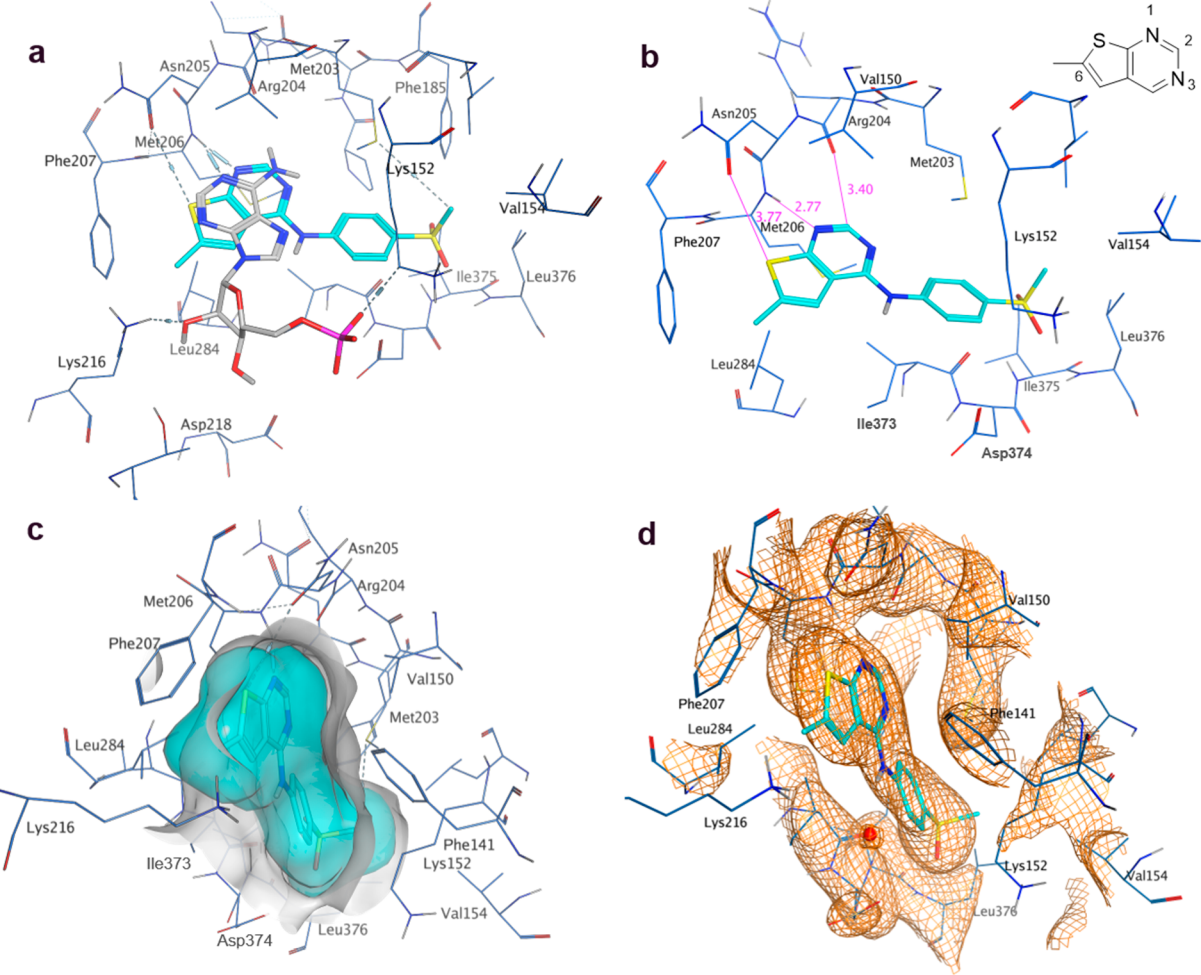
(a) AMP cofactor of PI5P4Kβ (3X01, gray) superposed onto chain A of PI5P4Kγ with **15** (8BQ4, blue); (b) binding pocket in chain A of the **15**-PI5P4Kγ complex with key interactions highlighted; 6-methyl thienylpyrimidine ring numbering shown (c) binding pocket in chain A of the **15**-PI5P4Kγ complex with ligand and receptor molecular surfaces; (d) electron density at 1σ for **15** in binding pocket.

There are two PI5P4Kγ homodimers in the asymmetric unit, as is seen in the 2GK9 apo structure of PI5P4Kγ, and the 7QIE structure with an allosteric ligand.^23^ Overall, the protein structure shows a high degree of similarity with that of apo structure 2GK9. Compound **15** occupies the pocket which is occupied by AMP/GMP in the PI5P4Kβ crystal structures (PDBs 3X01 and 3X02; Figure 5a).^29^ In both chains, **15** binds deeply in the hydrophobic cleft that forms the ATP binding site in lipid kinases. However, **15** binds in a position in which the heterocycle is rotated approximately 90° within the plane from the cofactor position. A hydrogen bond is formed between the ring sulfur and the side chain of Asn205 and also between the N1 nitrogen and the main chain NH of Met206. The hydrogen atom attached to C2 makes an aromatic hydrogen bond to the backbone carbonyl of Arg 204 (Figure 5b). Interestingly, pyrimidine N3, which, together with the amine linker, forms a common kinase binding motif, is in this case not involved in hydrogen bonding. The methyl group extending from the 6-position of the thienylpyrimidine ring makes contact with the side chain of Lys216 and also the phenyl ring of Phe207. The methanesulfonylphenyl moiety then runs deep along the back of the binding cavity, forming mainly hydrophobic interactions with residues Lys152, Met203, Ile373, Asp374, and Leu376 (Figure 5c). Overall, the ligand shows an excellent fit into the active site. Both the active site residues and ligand **15** are generally well-defined in the electron density map (Figure 5d). However, there is little electron density for either of the methyl groups, and the side chains of Lys216 and Lys152 are only partly defined. Only the backbone of Leu376 has clear electron density, so the exact arrangement of the binding pocket near the sulfonylmethyl is somewhat unclear. The activation loop formed from residues 377–402 is disordered.

**Figure 6:**
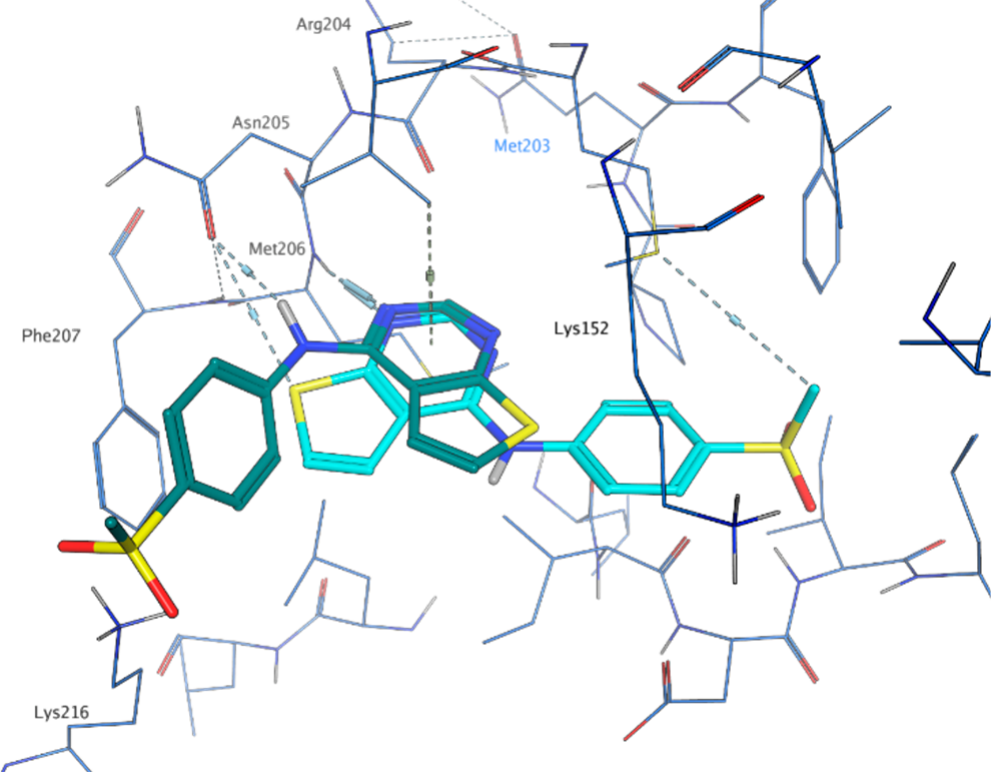
Docked pose of **15** (2GK9; teal) vs X-ray pose of **15** (8BQ4; cyan).

The crystal structure of **15** was obtained late in the program, after many analogues had been purchased or synthesized. Compound design was therefore conducted mostly based on existing SAR or on docking. Docking of **1** and **15** in the 2GK9 apo crystal structure (with Phe207 adjusted to the equivalent position in β structure 3X01 to open the active site) using constraints on Arg204O and Met206NH to favor hinge interaction resulted in a docking pose that showed the pyrimidine ring and amine linker rotated 90° with respect to the position of **15** in the crystal structure (Figure 6). This docking pose scores equally well to the crystal structure pose, but the ligand has a higher internal energy. This docking model led to the design of some of the homologated sulfones and lactams that were targeting an improved interaction with Lys216, e.g., compounds **26** and **34** (Table 3). However, later docking these to the structure obtained with **15** shows that these do not in fact fit as well to the binding site as **15**, a finding which agrees with their poorer enzyme inhibition. The pocket where the sulfone of **15** is located is lined with lipophilic residues: Val154, Phe185, Leu201, Met203, Ile375, and Leu376. Increasing the interactions with these residues by adding lipophilic groups to the sulfone increases potency, as seen in **22**, **23**, and **25** (Table 3). The sulfone does not make clear H-bonding interactions in the crystal structure, although a hydrogen bond with the backbone NH of Ile375 is possible. The potencies of **27** and **7** indeed demonstrate that a single acceptor is sufficient and can lead to better potency. However, the absence of an acceptor near Ile375 appears to be detrimental to potency, as illustrated by **10**.

The crystal structure binding mode of **15** suggests that CN and CF_3_ substitutions on thiophene (**41** and **42**, respectively) potentially make interactions with Lys216. Larger substituents on the thienylpyrimidine ring, such as the phenyl in example **43**, would not appear to fit in the binding pocket, but the potency of **43** suggests that the Phe207 and Lys216 side chains can move a little to accommodate an aromatic ring.

The crystal structure shows that N1 of the thienylpyrimidine ring is involved in a hydrogen bond with the backbone NH of Met206. Compound **12** cannot make this hydrogen bond, so it is surprising that this is equipotent to **1**. Docking suggests that **12** may bind with the pyridine nitrogen at the 5-position, making an interaction with Met260NH instead, and the NH interacting with the side chain of Asn205, similar to the docked pose in Figure 6.

## Conclusions

The PI5P4Ks have a rich biology which has been emerging in recent years. These targets have potential therapeutic benefit in conditions as wide-ranging as cancer, immunological disorders, and neurodegeneration. Further exploration of this biology and the development of drug candidates have been hampered by limited availability of high quality, selective tool molecules. Here, we have described a number of thienylpyrimidine PI5P4Kγ inhibitors with a range of physicochemical properties. This chemotype has afforded tool molecules with low nM PI5P4Kγ potency, good target engagement in cells, and excellent selectivity vs other kinases, including the other PI5P4K isoforms. The pharmacokinetic parameters of exemplars from the chemical series have been described, and several compounds display long in vivo half-lives (>2 h) and good brain penetration in mice, properties which make these molecules amenable to studying a wide range of biological processes in animal models. Furthermore, an X-ray structure of the **15**-PI5P4Kγ complex is provided, which may allow other groups to further develop drug candidates.

## Experimental Section

### Biochemical Assays

Assays to determine kinase activity of PI5P4Ks in the presence of inhibitors and target engagement of PI5P4Kγ in intact cells were performed as described previously.^23^ Recombinant mutant PI5P4Kγ+ was prepared as described previously.^19^ The protein from PIP4K2C (UniGene 6280511), genetically modified to have a specific activity close to that of the active PI5P4Kα isoform^19^ and cloned into the expression vector pGEX6P (Cytiva), was expressed and purified from *Escherichia coli* BL21(DE3). Cultures were induced with 0.4 mM IPTG, and probe sonicated in the presence of protease inhibitors. The GST fusion protein of PI5P4Kγ+ was harvested by binding to glutathione sepharose beads (Cytiva) and cleaved in situ with 50U of PreScission protease (Cytiva) for 4 h at 4 °C. The cleaved protein was further purified by size-exclusion chromatography (ÄKTA Pure, Cytiva). The protein purity was confirmed by sodium dodecyl sulfate–polyacrylamide gel electrophoresis, and the concentration was determined by colorimetric assay (Bio-Rad). Untagged wild-type protein was similarly prepared for PI5P4Kα (*PIP4K2A*; UniGene 138363) and PI5P4Kβ (*PIP4K2B*; UniGene 171988). For some applications, GST-tagged protein was also produced by column chromatography, initially using a GSTrap FF affinity column followed by size-exclusion chromatography (ÄKTA Pure, Cytiva).

PI5P4K activity in the presence of inhibitor compounds was determined by the ADP-Glo assay (Promega) as previously described.^23^ The binding of compounds to PI5P4Kγ in intact cells was assessed using an InCELL Pulse thermal stabilization assay (DiscoverX) as previously described^23^ and luminescence read using a Pherastar FSX plate reader (BMG Labtech).

### Data Analysis

Statistical analysis was performed using nonparametric testing in Prism 8 (GraphPad). Activity pIC_50_ values and in vivo binding pEC_50_ values were estimated using a 4-parameter fit (Dotmatics).

### X-ray Crystallography and Structure Determination

Crystallography was performed by Peak Proteins Ltd. Truncated human PI5P4Kγ was expressed in *E. coli* BL21(DE3) Gold using a pET28b vector. Expression was induced using 0.1 mM IPTG and the cells cultured at 18 °C for 16 h before harvesting by centrifugation. The protein comprised of residues His32 to Ala421 with the region between residues 309 and 331 deleted. Purification of TEV-cleaved protein was by both affinity and size exclusion (Superdex 75) chromatography. The structure of the ligand complex was generated by cocrystallization of human PI5P4Kγ in the presence of **15**. Purified protein (15.5 mg/mL in 20 mM HEPES pH 7.5, 150 mM NaCl, 0.5 mM TCEP) was incubated with 10 mM **15** (from 400 mM stock in DMSO) overnight at 4 °C. Crystals were grown from 20% w/v PEG3350 and 0.3 M ammonium tartrate at 20 °C. For X-ray data collection, they were flash-frozen and X-ray diffraction data were collected (I03 beamline, Diamond Light Source Synchrotron Facility, Oxford, UK) at 100 K. Data were processed using the XDS and Aimless software. The phase information necessary to determine and analyze the structure was obtained by molecular replacement (PHASER, CCP4) using the previously solved structure of a human PI5P4Kγ (PDB 2GK9) as the search model. Subsequent model building and refinement was performed according to standard protocols with the software packages CCP4 and COOT. TLS refinement (REFMAC5, CCP4) has been carried out, which resulted in lower *R*-factors and higher quality of the electron density map. The ligand parametrization and the generation of the corresponding library files was carried out with ACEDRG (CCP4). The water model was built with the “Find waters” algorithm of COOT by putting water molecules in peaks of the Fo–Fc map contoured at 3.0σ, followed by refinement with REFMAC5 and checking all waters with the validation tool of COOT. The criteria for the list of suspicious waters were: *B* factor greater 80 Å2, 2Fo–Fc map less than 1.2σ, distance to closest contact less than 2.3 Å or more than 3.5 Å. The suspicious water molecules and those in the active site (distance to inhibitor less than 10 Å) were checked manually. The occupancy of side chains, which were in negative peaks in the Fo–Fc map (contoured at −3.0σ), were set to zero and subsequently to 0.5 if a positive peak occurred after the next refinement cycle. Parameterization and the generation of the corresponding library files was carried out with ACEDRG (CCP4).

The Ramachandran plot of the final model shows 95.5% of all residues in the most favored region, 4.3% in the additionally allowed region. One residue (Arg336) was observed to be the disallowed region, but experimental electron density supports the observed conformation. Statistics of the final structure and the refinement process are presented in Supporting Information.

### Computational Modeling

The kinase-like subset of the BioAscent compound cloud library (https://compoundcloud.bioascent.com/collection-details/) was filtered to remove PAINS compounds (https://github.com/rdkit/rdkit/tree/master/Data/Pains), compounds with reactive groups^30^ (both implemented in RDKit in Knime), and compounds with undesirable properties (compounds with >4 aromatic rings, >10 rotatable bonds, >3 hydrogen bond donors, clogP >5, TPSA >125). The compounds were (de) protonated where this was their likely state at pH 7.0, converted to 3D, and tautomers and stereoisomers were generated using MOE command line tools (sdwash and sdstereo; release 2016.0802, Chemical Computing Group, www.chemcomp.com). The filtered set was docked to PI5P4Kα crystal structure 2YBX using GOLD (library screening setting, ChemScore kinase scoring, Hbond constraints Asn198 C=O acceptor–Val199 NH donor; GOLD 2017 release, CCDC, www.ccdc.cam.ac.uk). The active site was defined using the GNP ligand from PI5P4Kβ PDB 3X04. The resulting docked poses were filtered by fitting them, using the docked pose coordinates, to a MOE pharmacophore consisting of 4 features (1.4 Å acceptor projection spheres on Val199NH, Lys209NZ; 1.4 Å donor projection spheres on Arg197O and Asn198OD1, 2 out of 4 required) with 1.4 Å exclusion spheres on all other active site atoms. The resulting 6148 hits were redocked with Glide SP (no constraints) (release 2016-3, Schrodinger, www.schrodinger.com), and Gold VS.

The top 1000 best-scoring compounds by GOLD Fitness ChemScore, GOLD Fitness ASP score, and GLIDE SP dock score were then pooled, resulting in 2057 unique compounds. The log *D* and PSA were calculated for these compounds using Marvin, and those with log *D* > 5 and PSA > 100 were removed, leaving 1743 compounds (Marvin 20.15, ChemAxon https://www.chemaxon.com).

These 1743 compounds were clustered into 960 clusters with Knime K-medoids (Morgan FP, radius 4, Tanimoto; Knime version 3.1.0, Knime analytics, www.knime.com). The best ranking compound from each cluster was selected. Unselected compounds from the top 50 hits were added to this, and 21 of the lowest ranking compounds removed to ensure a set of 960 (3 plates).

Dockings for compound design and SAR analysis were done using Glide SP using the PDB 2GK9 structure with H-bonding constraints on Arg204O and Met206NH (1 of 2 required) or using the compound **15** crystal structure once available (no constraints).

XlogPs were calculated using Dotmatics (https://www.dotmatics.com/).

### Chemical Synthesis

#### Standard Techniques

Compounds **2**, **3**, **4**, and **5** were purchased from BioAscent and were tested as supplied. Compounds **6**, **9**, **10**, and **11** were purchased from Enamine Ltd. and were determined by UPLC to have purity >95%. All other compounds were synthesized as described below, and all tested compounds have purity >95% by UPLC analysis. Reagents and solvents were of commercially available reagent grade quality and used without further purification. Reactions requiring anhydrous conditions were carried out in oven-dried glassware under an atmosphere of N_2_. Reactions were monitored by thin-layer chromatography on silica gel 60 F_254_ aluminum or glass supported sheets or by liquid chromatography–mass spectrometry (LCMS). Flash column chromatography was carried out on a Biotage Isolera One system using normal phase (SiO_2_) cartridges. Compounds were loaded in solution or adsorbed onto Celite 545 and eluted using a linear gradient of the specified solvents. Purification by C18 reverse phase HPLC was carried using an Agilent 1260 Infinity machine and a Waters XBridge BEH C18 OBD column [(i) 130 Å, 5 μm, 30 mm × 100 mm; (ii) 130 Å, 5 μm, 19 mm × 250 mm] with a linear gradient of H_2_O (with 0.1% NH_3_) and MeCN (with 0.1% NH_3_). LCMS analysis was performed on a Waters Aquity HClass UPLC system with an Aquity QDa for mass detection. High-resolution mass spectra (HRMS) were measured on a Waters Vion IMS QTof spectrometer. NMR spectra were recorded on a Bruker Advance III (^1^H = 300 MHz, ^19^F = 282 MHz, ^13^C = 75 MHz) spectrometer using the requisite solvent as a reference for internal deuterium lock. The chemical shift data for each signal are given as δ chemical shift (multiplicity, *J* values in Hz, integration) in units of parts per million (ppm) relative to tetramethylsilane (TMS) where δH (TMS) = 0.00 ppm. The multiplicity of each signal is indicated by s (singlet), d (doublet), t (triplet), q (quartet), quin (quintet), or m (multiplet). Signals from exchangeable protons were not always detected. UPLC analysis of final compounds was performed on a Waters Aquity HClass UPLC system and is reported as method name, retention time, UV% purity. UPLC method parameters are detailed in Supporting Information, Table S11.

#### General Synthesis Procedures

##### General Procedure 1

A solution of the requisite aryl chloride (1.0 equiv) and the requisite aniline (1.0 equiv) in 4 M HCl in dioxane (0.1 M reaction concentration) was stirred at 100 °C for 16 h. Purification was achieved via the stated method.

##### General Procedure 2

A microwave flask was charged with the requisite aryl chloride (1.0 equiv) and the requisite aniline (1.0 equiv). The mixture was taken up in IPA (0.1 M reaction concentration), sealed, and heated under μW irradiation at 120 °C for 2 h. The resulting solid was filtered, washed with MeOH, and dried under high vacuum overnight. Purification was achieved via the stated method, if necessary.

##### General Procedure 3

A microwave flask was charged with the requisite aryl chloride (1.0 equiv), the requisite aniline (1.0 equiv), cesium carbonate (2.0 equiv), and Xantphos (0.02 equiv). The mixture was taken up in toluene (0.1 M reaction concentration) and degassed. Tris(dibenzylideneacetone) dipalladium(0) chloroform adduct (0.01 equiv) was added, and the reaction vessel degassed once more, sealed, and heated under μW irradiation at the stated temperature and time. Purification was achieved via the stated method.

##### General Procedure 4

A microwave flask was charged with the requisite aryl chloride (1.0 equiv), the requisite aniline (1.1 equiv), cesium carbonate (3.0 equiv), and Xantphos (0.10 equiv). The mixture was taken up in toluene (0.1 M reaction concentration) and degassed. Tris(dibenzylideneacetone) dipalladium(0) chloroform adduct (0.05 equiv) was added, and the reaction vessel degassed once more, sealed, and heated under μW irradiation at the stated temperature and time. Purification was achieved via the stated method.

#### Synthesis Procedures

##### *N*-(4-Methylsulfonylphenyl)thieno[2,3-*d*]pyrimidin-4-amine (**1**)

2,4-Dichloroquinazoline (50.0 mg, 0.250 mmol) and 4-(methylsulfonyl) aniline hydrochloride (52.2 mg, 0.250 mmol) were reacted according to general procedure 1 and the reaction cooled. EtOAc was added to the mixture and washed with satd. aq NaHCO_3_ (×1) and brine (×1), dried (MgSO_4_), and solvent removed in vacuo. Purification via silica gel chromatography (gradient elution 20–100% EtOAc in petroleum ether) yielded *N*-(4-methylsulfonylphenyl) thieno[2,3-*d*]pyrimidin-4-amine **1** (42.5 mg, 0.139 mmol, 55%) as a beige solid. MS (ESI+) *m*/*z* calcd for C_13_H_12_N_3_O_2_S_2_^+^ 306.4 [M + H]^+^, found 306.0. UPLC (method A) *t*_R_ = 3.68 min, >98%. ^1^H NMR (300 MHz, DMSO-*d*_6_) δ 10.05 (s, 1H), 8.63 (s, 1H), 8.23–8.14 (m, 2H), 8.00–7.87 (m, 3H), 7.83 (d, *J* = 6.0 Hz, 1H), 3.20 (s, 3H).

##### 1-(4-(Thieno[2,3-*d*]pyrimidin-4-ylamino)phenyl)pyrrolidin-2-one (**7**)

1-(4-Aminophenyl)-2-pyrrolidinone (50.0 mg, 0.280 mmol) and 4-chlorothieno[2,3-*d*]pyrimidine (50.8 mg, 0.300 mmol) were reacted according to general procedure 4 under μW irradiation at 120 °C for 1.5 h. Then the mixture was loaded onto an SCX-2 column, washed with MeOH, eluted with 0.5 M NH_3_ in MeOH, and concentrated in vacuo. Purification via preparatory HPLC (gradient elution 15–95% MeCN in H_2_O with 0.1% NH_3_) yielded 1-(4-(thieno[2,3-*d*]pyrimidin-4-ylamino) phenyl) pyrrolidin-2-one **7** (11 mg, 0.035 mmol, 12.5% yield) as a lyophilized white solid. MS (ESI+) *m*/*z* calcd for C_16_H_15_N_4_OS^+^ 311.1 [M + H]^+^, found 311.0. HRMS (ESI+) *m*/*z* calcd for C_16_H_15_N_4_OS^+^ 311.0967 [M + H]^+^, found 311.0971. UPLC analysis (method E), 3.72 min, >98%. 1H NMR (300 MHz, DMSO-*d*_6_) δ 9.69 (s, 1H), 8.48 (s, 1H), 7.92–7.77 (m, 3H), 7.77–7.62 (m, 3H), 3.85 (t, *J* = 7.0 Hz, 2H), 2.50–2.46 (m, 2H), 2.08 (tt, *J* = 7.7, 6.8 Hz, 2H).

##### 1-[2-Methoxy-4-(thieno[2,3-*d*]pyrimidin-4-ylamino)phenyl]pyrrolidin-2-one (**8**)

4-Chlorothieno[2,3-*d*]pyrimidine (50.0 mg, 0.290 mmol) and 1-(4-amino-2-methoxy-phenyl) pyrrolidin-2-one (58.6 mg, 0.280 mmol) were reacted according to general procedure 2 for 1.5 h at 120 °C. Upon cooling to rt the reaction mixture was loaded onto an SCX-II column, washed with MeOH, then eluted with 0.5 M NH_3_ in MeOH and concentrated in vacuo. Purification via preparatory HPLC (gradient elution 20–60% MeCN in H_2_O with 0.1% NH_3_) yielded 37.4 mg of a white solid. ^1^H NMR showed significant amounts of grease, hence the product was loaded onto an SCX-II column and washed through with MeOH. The product was eluted with 0.5 M NH_3_ in MeOH and solvent removed in vacuo to give 1-[2-methoxy-4-(thieno[2,3-*d*]pyrimidin-4-ylamino) phenyl]pyrrolidin-2-one **8** (34.9 mg, 0.103 mmol, 35%) as an off-white solid. MS (ESI+) *m*/*z* calcd for C_17_H_17_N_4_O_2_S^+^ [M + H]^+^ 341.1, found 341.1. UPLC (method D) *t*_R_ = 3.74 min, >98%. ^1^H NMR (300 MHz, chloroform-*d*) δ 8.56 (s, 1H), 8.41 (s, 1H), 7.59 (d, *J* = 6.0 Hz, 1H), 7.37 (dd, *J* = 4.0, 2.0 Hz, 2H), 7.05 (dd, *J* = 8.4, 2.0 Hz, 1H), 7.01 (d, *J* = 8.4 Hz, 1H), 3.75 (t, *J* = 7.1 Hz, 2H), 3.63 (s, 3H), 2.69 (dd, *J* = 8.6, 7.6 Hz, 2H), 2.26 (p, *J* = 7.6 Hz, 2H).

##### *N*-(4-(Methylsulfonyl)phenyl)thieno[3,2-*c*]pyridin-4-amine (**12**)

4-Chlorothieno[3,2-*c*]-pyridine (50.0 mg, 0.290 mmol) and 4-(methylsulfonyl) aniline hydrochloride (67.3 mg, 0.320 mmol) were reacted according to general procedure 4 at 120 °C for 1.5 h. Upon completion, the mixture was loaded onto an SCX-2 column and washed through with MeOH. The product was eluted with 0.5 M NH_3_ in MeOH and concentrated in vacuo. Purification via silica gel chromatography (gradient elution 10–100% EtOAc in petroleum ether) followed by preparatory HPLC (gradient elution 10–70% MeCN in H_2_O with 0.1% NH_3_), followed by silica gel chromatography (gradient elution 0–10% MeOH in DCM) yielded *N*-(4-(methylsulfonyl) phenyl) thieno[3,2-*c*]pyridin-4-amine **12** (13 mg, 0.043 mmol, 14% yield) as a white solid. MS (ESI+) *m*/*z* calcd for C_14_H_13_N_2_O_2_S_2_^+^ [M + H]^+^ 305.0, found 305.1. UPLC analysis (method F), 3.14 min, 97%. ^1^H NMR (300 MHz, DMSO-*d*_6_) δ 9.57 (s, 1H), 8.22–8.11 (m, 2H), 8.10–8.01 (m, 2H), 7.90–7.79 (m, 3H), 7.61 (dd, *J* = 5.6, 0.8 Hz, 1H), 3.17 (s, 3H).

##### *N*-(4-(Methylsulfonyl)phenyl)thieno[2,3-*b*]pyridin-4-amine (**13**)

4-Chloro-thieno[2,3-*b*]pyridine (50 mg, 0.29 mmol) and 4-(methylsulfonyl) aniline hydrochloride (67 mg, 0.32 mmol), Xantphos (17 mg, 0.030 mmol), and cesium carbonate (288 mg, 0.880 mmol), then toluene (3.3 mL) was added and the reaction degassed by passing N_2_ through for 5 min. Then tris(dibenzylideneacetone) dipalladium(0) chloroform adduct (15 mg, 0.010 mmol) was added, the reaction mixture degassed again, then the reaction was heated under microwave irradiation at 120 °C for 1.5 h. LCMS had no indication of product, so additional palladium catalyst (15 mg) was added, the reaction redegassed, and heated under microwave irradiation at 120 °C for 1.5 h. Then the mixture was loaded onto an SCX-2 column and washed through with MeOH. The product was eluted with 0.5 M NH_3_ in MeOH and concentrated in vacuo. Purification via silica gel chromatography (gradient elution 8–100% EtOAc in petroleum ether) yielded *N*-(4-(methylsulfonyl)phenyl)thieno[2,3-*b*]pyridin-4-amine **13** (25 mg, 0.084 mmol, 28% yield) as a white solid. MS (ESI+) *m*/*z* calcd for C_14_H_13_N_2_O_2_S_2_^+^ [M + H]^+^ 305.0, found 305.1. UPLC analysis (method D), 3.72 min, >98%. ^1^H NMR (300 MHz, chloroform-*d*) δ 8.45 (d, *J* = 5.4 Hz, 1H), 7.99–7.88 (m, 2H), 7.52 (d, *J* = 6.3 Hz, 1H), 7.39–7.32 (m, 2H), 7.30 (d, *J* = 6.1 Hz, 1H), 7.18 (dd, *J* = 5.4, 0.4 Hz, 1H), 6.68 (s, 1H), 3.11 (s, 3H).

##### 2-Methyl-*N*-(4-methylsulfonylphenyl)thieno[2,3-*d*]pyrimidin-4-amine (**14**)

4-(Methylsulfonyl) aniline hydrochloride (124 mg, 0.596 mmol) and 4-chloro-2-methylthieno[2,3-*d*]pyrimidine (100 mg, 0.542 mmol) were reacted according to general procedure 1. The reaction was cooled to rt and 0.5 M NH_3_ in MeOH (20 mL) added and solvent removed in vacuo. Purification via preparatory HPLC (gradient elution 10–50% MeCN in H_2_O with 0.1% NH_3_) yielded 2-methyl-*N*-(4-methylsulfonylphenyl)thieno[2,3-*d*]pyrimidin-4-amine **14** (113 mg, 0.354 mmol, 65%) as a white solid. MS (ESI+) *m*/*z* calcd for C_14_H_14_N_3_O_2_S_2_^+^ [M + H]^+^ 320.4, found 320.1. UPLC analysis (method D), 3.92 min, >98%. ^1^H NMR (300 MHz, chloroform-*d*) δ 8.08–7.89 (m, 4H), 7.46–7.28 (m, 3H), 3.10 (s, 3H), 2.76 (s, 3H).

##### 6-Methyl-*N*-(4-(methylsulfonyl)phenyl)thieno[2,3-*d*]pyrimidin-4-amine (**15**)

4-Chloro-6-methylthieno[2,3-*d*]pyrimidine (150 mg, 0.810 mmol, 1.0 equiv) and 4-(methylsulfonyl) aniline hydrochloride (186 mg, 0.890 mmol, 1.1 equiv) were reacted according to general procedure 1. The crude reaction was purified via preparatory HPLC (gradient elution 5–95% MeCN in H_2_O with 0.1% NH_3_) to give 6-methyl-*N*-(4-(methylsulfonyl)phenyl)thieno[2,3-*d*]pyrimidin-4-amine **15** (77.3 mg, 0.298 mmol, 37% yield) as a white lyophilized solid. MS (ESI+) *m*/*z* calcd for C_14_H_14_N_3_O_2_S_2_^+^ [M + H]^+^ 320.1, found 320.1. HRMS (ESI+) *m*/*z* calcd for C_14_H_14_N_3_O_2_S_2_^+^ 320.0527 [M + H]^+^, found 320.0529. UPLC analysis (method D), 4.05 min, >98%. ^1^H NMR (300 MHz, DMSO-*d*_6_) δ 9.89 (s, 1H), 8.56 (s, 1H), 8.22–8.11 (m, 2H), 7.97–7.85 (m, 2H), 7.63 (q, *J* = 1.2 Hz, 1H), 3.19 (s, 3H), 2.62 (d, *J* = 1.2 Hz, 3H). ^13^C NMR (75 MHz, DMSO) δ 167.09, 153.59, 152.65, 144.81, 138.48, 134.27, 128.44, 120.53, 118.61, 117.32, 44.39, 16.59.

##### 1-Methyl-*N*-(4-methylsulfonylphenyl)pyrazolo[3,4-*d*]pyrimidin-4-amine (**16**)

4-Chloro-1-methyl-1*H*-pyrazolo[3,4-*d*]pyrimidine (51.2 mg, 0.300 mmol) and 4-(methylsulfonyl) aniline hydrochloride (63.1 mg, 0.300 mmol) were reacted according to general procedure 1. The reaction was cooled to rt, diluted with EtOAc, then washed with satd. aq NaHCO_3_ (×1) and H_2_O (×2), dried (phase separator column) and solvent removed in vacuo. Purification via silica gel chromatography (gradient elution 0–10% MeOH in DCM) yielded 1-methyl-*N*-(4-methylsulfonylphenyl)pyrazolo[3,4-*d*]pyrimidin-4-amine **16** (21.6 mg, 0.071 mmol, 23%) as a white solid. MS (ESI+) *m*/*z* calcd for C_13_H_14_N_5_O_2_S^+^ [M + H]^+^ 304.1, found 304.1. UPLC (method D) *t*_R_ = 3.11 min, >98%. ^1^H NMR (300 MHz, DMSO-*d*_6_) δ 10.48 (s, 1H), 8.55 (s, 1H), 8.38 (s, 1H), 8.19 (d, *J* = 8.8 Hz, 2H), 7.93 (d, *J* = 8.9 Hz, 2H), 3.99 (s, 3H), 3.20 (s, 3H).

##### 7-Methyl-*N*-(4-(methylsulfonyl)phenyl)-7*H*-pyrrolo[2,3-*d*]pyrimidin-4-amine (**17**)

4-Chloro-7-methyl-7*H*-pyrrolo[2,3-*d*]pyrimidine (44.5 mg, 0.266 mmol) and 4-(methylsulfonyl) aniline (50 mg, 0.29 mmol) were reacted according to general procedure 2. LCMS indicated a poor conversion, so 4 M HCl in dioxane (0.1 mL) was added, and the reaction was stirred at rt for 2 h. After this time, the reaction was diluted with EtOAc (50 mL), washed with satd. aq NaHCO_3_ (2 × 50 mL) and brine (50 mL), then dried (hydrophobic frit) and concentrated in vacuo. Purification via preparatory HPLC (gradient elution 5–50% MeCN in H_2_O with 0.1% HCO_2_H) yielded 7-methyl-*N*-(4-(methylsulfonyl) phenyl)-7*H*-pyrrolo[2,3-*d*]pyrimidin-4-amine **17** (1.7 mg, 0.006 mmol, 2% yield) as a lyophilized white solid. MS (ESI+) *m*/*z* calcd for C_14_H_15_N_4_O_2_S^+^ [M + H]^+^ 303.1, found 303.2. UPLC analysis (method F), 3.00 min, 96%. ^1^H NMR (300 MHz, methanol-*d*_4_) δ 8.43 (s, 1H), 8.24–8.14 (m, 2H), 7.97–7.86 (m, 2H), 7.26 (d, *J* = 3.6 Hz, 1H), 6.84 (d, *J* = 3.6 Hz, 1H), 3.86 (s, 3H), 3.14 (s, 3H), exchangeable N*H* signal not observed.

##### 9-Methyl-*N*-(4-methylsulfonylphenyl)purin-6-amine (**18**)

4-(methylsulfonyl) aniline hydrochloride (50.0 mg, 0.240 mmol) and 6-chloro-9-methyl-9*H*-purine (44.65 mg, 0.260 mmol) were reacted according to general procedure 1. The reaction was cooled to rt and 0.5 M NH_3_ in MeOH (20 mL) added and solvent removed in vacuo. Purification via preparatory HPLC (gradient elution 10–50% MeCN in H_2_O with 0.1% NH_3_) yielded 9-methyl-*N*-(4-methylsulfonylphenyl) purin-6-amine **18** (25.7 mg, 0.085 mmol, 35%) as a white solid. MS (ESI+) *m*/*z* calcd for C_13_H_14_N_5_O_2_S^+^ [M + H]^+^ 304.1, found 304.1. UPLC (method D) *t*_R_ = 2.92 min, >98%. ^1^H NMR (300 MHz, chloroform-*d*) δ 8.66 (s, 1H), 8.16 (s, 1H), 8.14–8.07 (m, 2H), 7.99–7.92 (m, 2H), 7.90 (s, 1H), 3.93 (s, 3H), 3.08 (s, 3H).

##### *N*-(4-(Methylsulfonyl)phenyl)imidazo[1,2-*a*]pyrimidin-5-amine (**19**)

5-Chloroimidazo[1,2-*a*]pyrimidine (45 mg, 0.29 mmol) and 4-(methylsulfonyl) aniline hydrochloride (67 mg, 0.32 mmol) were reacted according to general procedure 4 at 120 °C for 1.5 h. Upon completion, the mixture was loaded onto an SCX-2 column and washed through with MeOH. The product was eluted with 0.5 M NH_3_ in MeOH and concentrated in vacuo. Purification via preparatory HPLC (gradient elution 5–50% MeCN in H_2_O with 0.1% NH_3_) yielded *N*-(4-methylsulfonylphenyl) imidazo[1,2-*a*]pyrimidin-5-amine **19** (23 mg, 0.080 mmol, 27% yield) as a white solid. MS (ESI+) *m*/*z* calcd for C_13_H_13_N_4_O_2_S^+^ [M + H]^+^ 289.1, found 289.1. UPLC analysis (method D), 2.65 min, >98%. ^1^H NMR (300 MHz, DMSO-*d*_6_) δ 10.18 (s, 1H), 8.67 (d, *J* = 7.3 Hz, 1H), 8.17–8.06 (m, 2H), 7.95–7.85 (m, 2H), 7.65 (d, *J* = 1.6 Hz, 1H), 7.40 (d, *J* = 1.6 Hz, 1H), 6.66 (d, *J* = 7.3 Hz, 1H), 3.18 (s, 3H).

##### *N*-(4-(Methylsulfonyl)phenyl)pyrrolo[2,1-*f*][1,2,4]triazin-4-amine (**47**)

2,4-Dichloropyrrolo[2,1-*f*][1,2,4]triazine (50 mg, 0.27 mmol) and 4-(methylsulfonyl) aniline (50 mg, 0.29 mmol) were reacted according to general procedure 2. Upon completion the reaction was diluted with EtOAc (50 mL), washed with satd. aq NaHCO_3_ (2 × 50 mL) and brine (50 mL), then dried (hydrophobic frit) and concentrated in vacuo. Purification via preparatory HPLC (gradient elution 20–70% MeCN in H_2_O with 0.1% NH_3_) yielded *N*-(4-(methylsulfonyl) phenyl) pyrrolo[2,1-*f*][1,2,4]triazin-4-amine **47** (27 mg, 0.084 mmol, 32% yield) as a lyophilized pale-yellow solid. MS (ESI+) *m*/*z* calcd for C_13_H_12_ClN_4_O_2_S^+^ [M + H]^+^ 323.0, found 323.1. UPLC analysis (method E), 4.63 min, >98%. ^1^H NMR (300 MHz, DMSO-*d*_6_) δ 10.62 (s, 1H), 8.14–8.04 (m, 2H), 8.03–7.92 (m, 2H), 7.88 (dd, *J* = 2.6, 1.5 Hz, 1H), 7.32 (dd, *J* = 4.6, 1.5 Hz, 1H), 6.83 (dd, *J* = 4.5, 2.6 Hz, 1H), 3.23 (s, 3H).

##### *N*-(4-(Methylsulfonyl)phenyl)pyrrolo[2,1-*f*][1,2,4]triazin-4-amine (**20**)

A solution of *N*-(4-(methylsulfonyl) phenyl) pyrrolo[2,1-*f*][1,2,4]triazin-4-amine **47** (24 mg, 0.070 mmol) in ethanol (3.7 mL) was flushed with N_2_. Palladium (10% on carbon) (7.9 mg, 0.010 mmol, 0.1 equiv) was added and the flask flushed again with N_2_. Then the flask was flushed with a balloon of H_2_ (×2) and stirred at rt for 96 h. After this time, further palladium (10% on carbon) (7.9 mg, 0.010 mmol, 0.1 equiv) was added and the flask reflushed with H_2_ and stirred for a further 72 h. Then the flask was flushed with N_2_, the reaction mixture diluted with ethanol (10 mL), then passed through a plug of Celite and concentrated in vacuo. Purification via preparatory HPLC (gradient elution 10–60% MeCN in H_2_O with 0.1% NH_3_) yielded *N*-(4-(methylsulfonyl)phenyl)pyrrolo[2,1-*f*][1,2,4]triazin-4-amine **20** (13.4 mg, 0.046 mmol, 63% yield) as a cream-colored lyophilized solid. MS (ESI+) *m*/*z* calcd for C_13_H_13_N_4_O_2_S^+^ [M + H]^+^ 289.1, found 289.2. UPLC analysis (method E), 3.83 min, 96%. ^1^H NMR (300 MHz, chloroform-*d*) δ 8.12 (s, 1H), 8.08–7.94 (m, 4H), 7.73 (dd, *J* = 2.6, 1.4 Hz, 1H), 6.86–6.74 (m, 2H), 3.10 (s, 3H), exchangeable N*H* signal not observed.

##### *N*-(4-(Methylsulfonyl)phenyl)-6,7-dihydro-5*H*-cyclopenta[*d*]pyrimidin-4-amine (**21**)

4-Chloro-6,7-dihydro-5*H*-cyclopentapyrimidine (41.0 mg, 0.266 mmol) and 4-(methylsulfonyl) aniline (50 mg, 0.29 mmol) were reacted according to general procedure 2. Upon completion, the reaction was diluted with EtOAc (50 mL), washed with satd. aq NaHCO_3_ (2 × 50 mL) and brine (50 mL), then dried (hydrophobic frit) and concentrated in vacuo. Purification via preparatory HPLC (gradient elution 10–60% MeCN in H_2_O with 0.1% NH_3_) yielded *N*-(4-(methylsulfonyl) phenyl)-6,7-dihydro-5*H*-cyclopenta[*d*]pyrimidin-4-amine **21** (19 mg, 0.066 mmol, 25% yield) as a lyophilized white solid. MS (ESI+) *m*/*z* calcd for C_14_H_16_N_3_O_2_S^+^ [M + H]^+^ 290.1, found 290.2. UPLC analysis (method E), 3.40 min, 98%. ^1^H NMR (300 MHz, chloroform-*d*) δ 8.68 (s, 1H), 7.91 (s, 4H), 6.71 (s, 1H), 3.11–3.00 (m, 5H), 2.92 (t, *J* = 7.4 Hz, 2H), 2.24 (p, *J* = 7.7 Hz, 2H).

##### *N*-(4-Isopropylsulfonylphenyl)thieno[2,3-*d*]pyrimidin-4-amine (**22**)

4-Chlorothieno[2,3-*d*]pyrimidine (75.0 mg, 0.440 mmol) and 4-(isopropylsulfonyl) aniline (87.6 mg, 0.440 mmol) were reacted according to general procedure 2. Purification via preparatory HPLC (gradient elution 30–70% MeCN in H_2_O with 0.1% NH_3_) yielded *N*-(4-isopropylsulfonylphenyl)thieno[2,3-*d*]pyrimidin-4-amine **22** (87.0 mg, 0.261 mmol, 59%) as a white solid. MS (ESI+) *m*/*z* calcd for C_15_H_16_N_3_O_2_S_2_^+^ [M + H]^+^ 334.1, found 334.2. UPLC (method D) *t*_R_ = 4.30 min, >98%. ^1^H NMR (300 MHz, DMSO-*d*_6_) δ 8.63 (s, 1H), 8.26–8.12 (m, 2H), 7.95 (d, *J* = 6.1 Hz, 1H), 7.87–7.83 (m, 2H), 7.81 (d, *J* = 6.0 Hz, 1H), 3.40–3.29 (m, 1H, partially obs. by HOD peak), 1.17 (d, *J* = 6.8 Hz, 6H), exchangeable N*H* not observed.

##### *N*-(4-Cyclopentylsulfonylphenyl)thieno[2,3-*d*]pyrimidin-4-amine (**23**)

4-(Cyclopentanesulfonyl) aniline (109 mg, 0.484 mmol) and 4-chlorothieno[2,3-*d*]pyrimidine (75.0 mg, 0.440 mmol) were reacted according to general procedure 1. The reaction was cooled to rt and 0.5 M NH_3_ in MeOH (20 mL) added and solvent removed in vacuo. Purification via preparatory HPLC (gradient elution 10–50% MeCN in H_2_O with 0.1% NH_3_) yielded *N*-(4-cyclopentylsulfonylphenyl) thieno[2,3-*d*]pyrimidin-4-amine **23** (120 mg, 0.334 mmol, 76%) as a white solid. MS (ESI+) *m*/*z* calcd for C_17_H_18_N_3_O_2_S_2_^+^ 360.1 [M + H]^+^, found 360.1. HRMS (ESI+) *m*/*z* calcd for C_17_H_18_N_3_O_2_S_2_^+^ 360.0840 [M + H]^+^, found 360.0842. UPLC analysis (method D), 4.77 min, >98%. ^1^H NMR (300 MHz, chloroform-*d*) δ 8.73 (s, 1H), 8.02–7.92 (m, 2H), 7.92–7.81 (m, 2H), 7.56 (s, 1H), 7.53–7.41 (m, 2H), 3.53 (tt, *J* = 8.7, 7.2 Hz, 1H), 2.21–2.02 (m, 2H), 2.01–1.86 (m, 2H), 1.86–1.73 (m, 2H), 1.71–1.58 (m, 2H). ^13^C NMR (75 MHz, DMSO) δ 167.54, 154.72, 153.32, 144.76, 132.08, 129.63, 125.29, 120.74, 119.92, 117.99, 63.40, 27.28, 26.00.

##### *N*-[4(Trifluoromethylsulfonyl)phenyl]thieno[2,3-*d*]pyrimidin-4-amine (**24**)

4-(Trifluoromethylsulfonyl) aniline (109 mg, 0.484 mmol) and 4-chlorothieno[2,3-*d*]pyrimidine (75.0 mg, 0.440 mmol) were reacted according to general procedure 1. The reaction was cooled to rt and 0.5 M NH_3_ in MeOH (20 mL) added and solvent removed in vacuo. Purification via preparatory HPLC (gradient elution 10–50% MeCN in H_2_O with 0.1% NH_3_) yielded *N*-[4 (trifluoromethylsulfonyl) phenyl]thieno[2,3-*d*]pyrimidin-4-amine **24** (133 mg, 0.369 mmol, 84%) as a white solid. MS (ESI+) *m*/*z* calcd for C_13_H_9_N_3_O_2_S_2_F_3_^+^ 360.0 [M + H]^+^, found 360.1 [M + H]^+^. HRMS (ESI+) *m*/*z* calcd for C_13_H_9_N_3_O_2_S_2_F_3_^+^ 360.0088 [M + H]^+^, found 360.0088. UPLC analysis (method D), 5.46 min, >98%. ^1^H NMR (300 MHz, chloroform-*d*) δ 8.80 (s, 1H), 8.24–8.12 (m, 2H), 8.11–8.01 (m, 2H), 7.57 (d, J = 6.0 Hz, 1H), 7.46–7.36 (m, 2H). ^13^C NMR (75 MHz, DMSO) δ 167.98, 154.40, 153.11, 148.64, 132.62, 125.96, 121.05, 120.08 (d, *J* = 326 Hz, *C*F_3_), 119.88, 118.48. ^19^F NMR (282 MHz, DMSO-*d*_6_) δ −78.82 (s, 3F).

##### *N*-(4-(Phenylsulfonyl)phenyl)thieno[2,3-*d*]pyrimidin-4-amine (**25**)

4-Chloro-6-methylthieno[2,3-*d*]pyrimidine (75.0 mg, 0.440 mmol, 1.0 equiv) and 4-(phenylsulfonyl) aniline (113 mg, 0.480 mmol) were reacted according to general procedure 1. The crude reaction was purified via preparatory HPLC (gradient elution 20–60% MeCN in H_2_O with 0.1% NH_3_) to give *N*-[4-(benzenesulfonyl) phenyl]thieno[2,3-*d*]pyrimidin-4-amine **25** (90.3 mg, 0.246 mmol, 56% yield) as a white lyophilized solid. MS (ESI+) *m*/*z* calcd for C_18_H_14_N_3_O_2_S_2_^+^ [M + H]^+^ 368.5, found 368.1. UPLC analysis (method D), 4.93 min, >98%. ^1^H NMR (300 MHz, chloroform-*d*) δ 8.70 (s, 1H), 8.03–7.87 (m, 6H), 7.65–7.46 (m, 4H), 7.38 (d, *J* = 6.1 Hz, 1H), 7.30 (s, 1H).

##### *N*-[4-(Methylsulfonylmethyl)phenyl]thieno[2,3-*d*]pyrimidin-4-amine (**26**)

4-Chlorothieno[2,3-*d*]pyrimidine (75.mg, 0.440 mmol) and 4-(methanesulfonylmethyl) aniline (81.43 mg, 0.440 mmol) were reacted according to general procedure 2 to give *N*-[4-(methylsulfonylmethyl)phenyl]thieno[2,3-*d*]pyrimidin-4-amine **26** (126 mg, 0.394 mmol, 90%) as a cream solid. MS (ESI+) *m*/*z* calcd for C_14_H_14_N_3_O_2_S_2_^+^ [M + H]^+^ 320.1, found 320.2. UPLC (method D) *t*_R_ = 3.41 min, >98%. ^1^H NMR (300 MHz, DMSO-*d*_6_) δ 10.05 (s, 1H), 8.57 (s, 1H), 7.98 (d, *J* = 6.0 Hz, 1H), 7.90–7.82 (m, 2H), 7.78 (d, *J* = 6.0 Hz, 1H), 7.46–7.39 (m, 2H), 4.48 (s, 2H), 2.92 (s, 3H).

##### *N*-(4-(3-Methyloxetan-3-yl)phenyl)thieno[2,3-*d*]pyrimidin-4-amine (**27**)

4-Chlorothieno[2,3-*d*]pyrimidine (50 mg, 0.29 mmol) and 4-(3-methyloxetan-3-yl) aniline (53 mg, 0.32 mmol) were reacted according to general procedure 4 at 100 °C for 1.5 h. Upon completion, the mixture was loaded onto an SCX-2 column and washed through with MeOH. The product was eluted with 0.5 M NH_3_ in MeOH and concentrated in vacuo. Product was detected by LCMS in both the wash and elution fractions, so both were carried forward to purification. Purification via preparatory HPLC (gradient elution 10–60% MeCN in H_2_O with 0.1% NH_3_) yielded *N*-(4-(3-methyloxetan-3-yl)phenyl)thieno[2,3-*d*]pyrimidin-4-amine **27** (17 mg, 0.057 mmol, 20% yield) as a white solid. MS (ESI+) *m*/*z* calcd for C_16_H_16_N_3_OS^+^ [M + H]^+^ 298.1, found 298.3. UPLC analysis (method D), 5.42 min, >95%. ^1^H NMR (300 MHz, chloroform-*d*) δ 8.62 (s, 1H), 7.70–7.59 (m, 2H), 7.40 (d, *J* = 6.0 Hz, 1H), 7.33–7.24 (m, 2H), 7.17 (d, *J* = 6.1 Hz, 1H), 5.00 (d, *J* = 5.5 Hz, 2H), 4.69 (d, *J* = 5.6 Hz, 2H), 1.77 (s, 3H), exchangeable N*H* signal not observed.

##### *N*-(4-Isoxazol-3-ylphenyl)thieno[2,3-*d*]pyrimidin-4-amine (**28**)

4-(1,2-Oxazol-3-yl) aniline hydrochloride (104 mg, 0.527 mmol) and 4-chlorothieno[2,3-*d*]pyrimidine (75.0 mg, 0.440 mmol) were reacted according to general procedure 3 in 1,4-dioxane (3 mL) at 130 °C for 30 min. The reaction was cooled to rt, filtered, and solvent removed in vacuo. Purification via preparatory HPLC (gradient elution 20–60% MeCN in H_2_O with 0.1% NH_3_) yielded *N*-(4-isoxazol-3-ylphenyl)thieno[2,3-*d*]pyrimidin-4-amine **28** (71.8 mg, 0.244 mmol, 55%) as a white solid. MS (ESI+) *m*/*z* calcd for C_15_H_11_N_4_OS^+^ [M + H]^+^ 295.3, found 295.2. UPLC analysis (method F), 4.43 min, >98%. ^1^H NMR (300 MHz, DMSO-*d*_6_) δ 9.87 (s, 1H), 8.99 (d, *J* = 1.7 Hz, 1H), 8.58 (s, 1H), 8.10–8.00 (m, 2H), 7.99–7.89 (m, 3H), 7.78 (d, *J* = 6.0 Hz, 1H), 7.14 (d, *J* = 1.7 Hz, 1H).

##### *N*-(4-Oxazol-2-ylphenyl)thieno[2,3-*d*]pyrimidin-4-amine (**29**)

4-(1,3-Oxazol-2-yl) aniline (113 mg, 0.703 mmol) and 4-chlorothieno[2,3-*d*]pyrimidine (100 mg, 0.586 mmol) were reacted according to general procedure 3 in 1,4-dioxane (3 mL) at 125 °C for 120 min. The reaction was cooled to rt, filtered, and solvent removed in vacuo. Purification via SCX-2 column eluting with 0.5 M methanolic ammonia and preparatory HPLC (gradient elution 20–60% MeCN in H_2_O with 0.1% NH_3_) yielded *N*-(4-oxazol-2-ylphenyl)thieno[2,3-*d*]pyrimidin-4-amine **29** (9.5 mg, 0.032 mmol, 6%) as a white solid. MS (ESI+) *m*/*z* calcd for C_15_H_11_N_4_OS^+^ [M + H]^+^ 295.3, found 295.2. UPLC analysis (method D), 2.91 min, >95%. ^1^H NMR (300 MHz, DMSO-*d*_6_) δ 9.91 (s, 1H), 8.59 (s, 1H), 8.20 (d, *J* = 0.8 Hz, 1H), 8.13–8.06 (m, 2H), 8.04–7.93 (m, 3H), 7.79 (d, *J* = 6.0 Hz, 1H), 7.36 (d, *J* = 0.8 Hz, 1H).

##### *N*-(5-Methylsulfonyl-2-pyridyl)thieno[2,3-*d*]pyrimidin-4-amine (**30**)

4-Chlorothieno[2,3-*d*]pyrimidine (50.0 mg, 0.290 mmol) and 5-(methylsulfonyl)-2-pyridinamine (50.46 mg, 0.290 mmol) were reacted according to general procedure 3 for 1.5 h at 120 °C. Upon cooling to rt, the reaction mixture was loaded onto an SCX-II column, washed with MeOH, then eluted with 0.5 M NH_3_ in MeOH and DCM (1:1) and concentrated in vacuo. Purification via preparatory HPLC (gradient elution 20–60% MeCN in H_2_O with 0.1% NH_3_) yielded *N*-(5-methylsulfonyl-2-pyridyl)thieno[2,3-*d*]pyrimidin-4-amine **30** (27.4 mg, 0.089 mmol, 31%) as a white solid. MS (ESI+) *m*/*z* calcd for C_12_H_11_N_4_O_2_S_2_^+^ [M + H]^+^ 307.0, found 307.1. UPLC (method D) *t*_R_ = 3.39 min, >98%. ^1^H NMR (300 MHz, DMSO-d6) δ 11.05 (s, 1H), 8.85 (dd, *J* = 2.6, 0.8 Hz, 1H), 8.74 (d, *J* = 9.4 Hz, 2H), 8.33 (dd, *J* = 9.0, 2.6 Hz, 1H), 8.15 (d, *J* = 6.0 Hz, 1H), 7.83 (d, *J* = 6.0 Hz, 1H), 3.31 (s, 3H).

##### *N*-Methyl-*N*-(4-methylsulfonylphenyl)thieno[2,3-*d*]pyrimidin-4-amine (**31**)

*N*-Methyl-4-(methylsulfonyl) aniline hydrochloride (107 mg, 0.484 mmol) and 4-chlorothieno[2,3-*d*]pyrimidine (75.0 mg, 0.440 mmol) were reacted according to general procedure 1. The reaction was cooled to rt and 0.5 M NH_3_ in MeOH (20 mL) added and solvent removed in vacuo. Purification via preparatory HPLC (gradient elution 10–50% MeCN in H_2_O with 0.1% NH_3_) yielded *N*-methyl-*N*-(4-methylsulfonylphenyl)thieno[2,3-*d*]pyrimidin-4-amine **31** (69.3 mg, 0.186 mmol, 42%) as a white solid. MS (ESI+) *m*/*z* calcd for C_14_H_14_N_3_O_2_S_2_^+^ [M + H]^+^ 320.4, found 320.1. UPLC analysis (method D), 3.67 min, 98%. ^1^H NMR (300 MHz, chloroform-*d*) δ 8.76 (s, 1H), 8.07–7.95 (m, 2H), 7.51–7.37 (m, 2H), 7.12 (d, J = 6.1 Hz, 1H), 6.01 (d, *J* = 6.1 Hz, 1H), 3.73 (s, 3H), 3.15 (s, 3H).

##### 4-(4-Methylsulfonylphenyl)sulfanylthieno[2,3-*d*]pyrimidine (**32**)

4-Chlorothieno[2,3-*d*]pyrimidine (55.0 mg, 0.320 mmol) was dissolved in DCM (3.5 mL) with triethylamine (0.09 mL, 0.640 mmol). 4-Methanesulfonylbenzene-1-thiol (60.69 mg, 0.320 mmol) was added and the reaction stirred at rt for 18 h. A resulting crystalline solid was filtered, washed with DCM, and dried under high vacuum to give 4-(4-methylsulfonylphenyl) sulfanylthieno[2,3-*d*]pyrimidine **32** (43.1 mg, 0.134 mmol, 41%). MS (ESI+) *m*/*z* calcd for C_13_H_11_N_2_O_2_S_3_^+^ [M + H]^+^ 323.0, found 323.0. UPLC (method D) *t*_R_ = 4.49 min, >98%. ^1^H NMR (300 MHz, DMSO-d6) δ 8.78 (s, 1H), 8.10–8.00 (m, 3H), 7.99–7.90 (m, 2H), 7.60 (d, *J* = 6.0 Hz, 1H), 3.33 (s, 3H).

##### *N*-[4-(Thieno[2,3-*d*]pyrimidin-4-ylamino)phenyl]acetamide Hydrochloride (**33**)

4-Chlorothieno[2,3-*d*]pyrimidine (100.0 mg, 0.586 mmol) and 4′-aminoacetanilide (88.0 mg, 0.586 mmol) were reacted according to general procedure 2. Purification via filtration and washing with MeOH yielded *N*-[4-(thieno[2,3-*d*]pyrimidin-4-ylamino)phenyl]acetamide hydrochloride **33** (163 mg, 0.508 mmol, 87%) as a pale-green solid. MS (ESI+) *m*/*z* calcd for C_14_H_12_N_4_OS^+^ [M + H]^+^ 285.1, found 285.2. UPLC (method D) *t*_R_ = 3.23 min, 100%. ^1^H NMR (300 MHz, DMSO-*d*_6_) δ 10.38 (s, 1H), 10.07 (s, 1H), 8.58 (s, 1H), 7.99 (d, *J* = 6.0 Hz, 1H), 7.80 (d, *J* = 5.9 Hz, 1H), 7.68 (d, *J* = 9.1 Hz, 2H), 7.63 (d, *J* = 9.3 Hz, 2H), 6.90 (brs, 1H), 2.06 (s, 3H).

##### 1-(4-(Thieno[2,3-*d*]pyrimidin-4-ylamino)benzyl)pyrrolidin-2-one (**34**)

A 5 mL microwave vial was charged with 4-chlorothieno[2,3-*d*]pyrimidine (75 mg, 0.44 mmol), 1-[(4-aminophenyl) methyl]pyrrolidin-2-one (92 mg, 0.48 mmol), and IPA (4.4 mL), then sealed and headed at 120 °C under μW irradiation for 2 h. Upon completion, the reaction was diluted with EtOAc (50 mL), washed with satd. aq NaHCO_3_ (50 mL) and brine (50 mL), then dried (MgSO_4_) and concentrated in vacuo to give 1-(4-(thieno[2,3-*d*]pyrimidin-4-ylamino)benzyl)pyrrolidin-2-one **34** (134 mg, 0.413 mmol, 94% yield) as a pale-green solid. MS (ESI+) *m*/*z* calcd for C_17_H_17_N_4_OS^+^ [M + H]^+^ 325.1, found 325.3. UPLC analysis (method F), 3.62 min, >98%. ^1^H NMR (300 MHz, chloroform-*d*) δ 8.55 (s, 1H), 7.63–7.53 (m, 2H), 7.39 (s, 1H), 7.33–7.23 (m, 2H), 7.19 (s, 2H), 4.39 (s, 2H), 3.25 (dd, *J* = 7.5, 6.6 Hz, 2H), 2.39 (t, *J* = 8.1 Hz, 2H), 2.03–1.87 (m, 2H).

##### *N*-(4-Pyrrolidin-1-ylsulfonylphenyl)thieno[2,3-*d*]pyrimidin-4-amine (**35**)

4-(1-Pyrrolidinylsulfonyl) aniline (99.5 mg, 0.440 mmol) and 4-chlorothieno[2,3-*d*]pyrimidine (75.0 mg, 0.440 mmol) were reacted according to general procedure 1 at 80 °C. The reaction was cooled to rt, and the resulting precipitate was filtered, washed with EtOAc, and taken up in DMSO with 0.5 M NH_3_ in MeOH until dissolution occurred. The MeOH was removed in vacuo and the DMSO solution used for purification. Purification via preparatory HPLC (gradient elution 40–80% MeCN in H_2_O with 0.1% NH_3_) yielded *N*-(4-pyrrolidin-1-ylsulfonylphenyl)thieno[2,3-*d*]pyrimidin-4-amine **35** (82.1 mg, 0.228 mmol, 52%) as a white solid. MS (ESI+) *m*/*z* calcd for C_16_H_17_N_4_O_2_S_2_^+^ [M + H]^+^ 361.1, found 361.2. HRMS (ESI+) *m*/*z* calcd for C_16_H_17_N_4_O_2_S_2_^+^ 361.0793 [M + H]^+^, found 361.0796. UPLC (method D) *t*_R_ = 4.87 min, >98%. ^1^H NMR (300 MHz, DMSO-*d*_6_) δ 10.02 (br s, 1H), 8.62 (s, 1H), 8.24–8.12 (m, 2H), 7.95 (d, *J* = 6.0 Hz, 1H), 7.88–7.77 (m, 3H), 3.21–3.10 (m, 4H), 1.73–1.58 (m, 4H). ^13^C NMR (75 MHz, DMSO) δ 167.48, 154.74, 153.34, 144.14, 129.79, 128.81, 125.20, 120.72, 119.93, 117.93, 48.28, 25.16.

##### *N*-[4-(3,3-Difluoropyrrolidin-1-yl) sulfonylphenyl]acetamide (**48**)

3,3-Difluoropyrrolidine hydrochloride (35.6 mg, 0.248 mmol) was taken up in DCM (10 mL) and triethylamine (0.09 mL, 0.68 mmol) and stirred for 1 min before adding dropwise to *N*-acetylsulfanilyl chloride (50.0 mg, 0.214 mmol) in DCM (2 mL) when complete dissolution occurred. The reaction was stirred at 21 °C overnight. Further DCM was added and the reaction mixture washed with satd. aq NaHCO_3_ (×1) and brine (×1), dried (Na_2_SO_4_), and solvent removed in vacuo to give *N*-[4-(3,3-difluoropyrrolidin-1-yl) sulfonylphenyl]acetamide **48** (67.0 mg, 0.214 mmol, 100%) as a white solid. MS (ESI+) *m*/*z* calcd for C_12_H_14_F_2_N_2_O_3_S^+^ [M + H]^+^ 305.1, found 305.2. UPLC (method C) *t*_R_ = 1.59 min, >98%. ^1^H NMR (300 MHz, chloroform-*d*) δ 7.76–7.60 (m, 4H), 7.37 (s, 1H), 3.47 (t, *J* = 12.9 Hz, 2H), 3.35 (t, *J* = 7.2 Hz, 2H), 2.29–2.13 (m, 2H), 2.17 (s, 3H).

##### 4-(3,3-Difluoropyrrolidin-1-yl) sulfonylaniline (**49**)

*N*-[4-(3,3-Difluoropyrrolidin-1-yl) sulfonylphenyl]acetamide **48** (67.0 mg, 0.214 mmol) was taken up in methanol (2 mL) and 1 M HCl aqueous (4 mL, 4 mmol) added. The reaction was heated at 85 °C for 3 h when the reaction mixture was basified with satd. aq NaHCO_3_ and extracted with EtOAc (×2). The combined organic layers were washed with brine (×1), dried (Na_2_SO_4_), and concentrated in vacuo to give 4-(3,3-difluoropyrrolidin-1-yl) sulfonylaniline **49** (56 mg, 0.214 mmol, 100%) as a colorless oil. ^1^H NMR (300 MHz, chloroform-*d*) δ 7.58–7.46 (m, 2H), 6.70–6.59 (m, 2H), 4.14 (brs, 2H), 3.44 (t, *J* = 13.1 Hz, 2H), 3.31 (t, *J* = 7.2 Hz, 2H), 2.20 (tt, *J* = 13.8, 7.2 Hz, 2H).

##### *N*-[4-(3,3-Difluoropyrrolidin-1-yl)sulfonylphenyl]thieno[2,3-*d*]pyrimidin-4-amine (**36**)

4-Chlorothieno[2,3-*d*]pyrimidine (35.8 mg, 0.210 mmol) and 4-(3,3-difluoropyrrolidin-1-yl) sulfonylaniline **49** (55.0 mg, 0.210 mmol) were reacted according to general procedure 2. Purification via preparatory HPLC (gradient elution 40–80% MeCN in H_2_O with 0.1% NH_3_) yielded *N*-[4-(3,3-difluoropyrrolidin-1-yl)sulfonylphenyl]thieno[2,3-*d*]pyrimidin-4-amine **36** (46.3 mg, 0.117 mmol, 56%) as a white solid. MS (ESI+) *m*/*z* calcd for C_16_H_15_F_2_N_4_O_2_S_2_^+^ [M + H]^+^ 397.1, found 397.3. HRMS (ESI+) *m*/*z* calcd for C_16_H_15_F_2_N_4_O_2_S_2_^+^ 397.0599 [M + H]^+^, found 397.0594. UPLC (method D) *t*_R_ = 4.89 min, >98%. ^1^H NMR (300 MHz, DMSO-*d*_6_) δ 10.05 (s, 1H), 8.64 (s, 1H), 8.28–8.16 (m, 2H), 7.96 (d, *J* = 6.0 Hz, 1H), 7.89–7.84 (m, 2H), 7.82 (d, *J* = 6.0 Hz, 1H), 3.59 (t, *J* = 13.1 Hz, 2H), 3.40–3.35 (m, obsd by HOD peak, 2H), 2.35 (tt, *J* = 14.3, 7.3 Hz, 2H). ^13^C NMR (75 MHz, DMSO) δ 167.54, 154.70, 153.31, 144.75, 129.19, 128.33 (t, *J* = 248.9 Hz, *C*F_2_), 128.18, 125.30, 120.72, 119.92, 118.01, 54.32 (t, *J* = 31.8 Hz, *C*H_2_CF_2_), 46.14, 34.02 (t, *J* = 23.9 Hz, *C*H_2_CF_2_). ^19^F NMR (282 MHz, DMSO-*d*_6_) δ −98.23 (quin, *J* = 13.6 Hz, C*F*_2_).

##### *N*,*N*-Dimethyl-4-(thieno[2,3-*d*]pyrimidin-4-ylamino)benzenesulfonamide (**37**)

*N*,*N*-Dimethylsulfanilamide (88.0 mg, 0.440 mmol) and 4-chlorothieno[2,3-*d*]pyrimidine (75.0 mg, 0.440 mmol) were reacted according to general procedure 1 at 80 °C. The reaction was cooled to rt, and the resulting precipitate was filtered, washed with EtOAc, and taken up in DMSO with 0.5 M NH_3_ in MeOH until dissolution occurred. The MeOH was removed in vacuo and the DMSO solution used for purification. Purification via preparatory HPLC (gradient elution 40–80% MeCN in H_2_O with 0.1% NH_3_) yielded *N*,*N*-dimethyl-4-(thieno[2,3-*d*]pyrimidin-4-ylamino) benzenesulfonamide **37** (95.7 mg, 0.286 mmol, 65%) as a white solid. MS (ESI+) *m*/*z* calcd for C_14_H_15_N_4_O_2_S_2_^+^ [M + H]^+^ 335.1, found 335.1. HRMS (ESI+) *m*/*z* calcd for C_14_H_15_N_4_O_2_S_2_^+^ 335.0631 [M + H]^+^, found 335.0635. UPLC (method D) *t*_R_ = 4.59 min, >98%. ^1^H NMR (300 MHz, DMSO-*d*_6_) δ 10.03 (s, 1H), 8.63 (s, 1H), 8.26–8.14 (m, 2H), 7.95 (d, *J* = 6.1 Hz, 1H), 7.81 (d, *J* = 6.0 Hz, 1H), 7.79–7.73 (m, 2H), 2.62 (s, 6H). ^13^C NMR (75 MHz, DMSO) δ 167.49, 154.74, 153.34, 144.24, 129.05, 128.19, 125.23, 120.71, 119.93, 117.94, 38.14.

##### *N*-Methyl-4-(thieno[2,3-*d*]pyrimidin-4-ylamino)benzenesulfonamide (**38**)

4-Chlorothieno[2,3-*d*]pyrimidine (75.0 mg, 0.440 mmol) and 4-amino-*N*-methyl-benzenesulfonamide (81.9 mg, 0.440 mmol) were reacted according to general procedure 2 to give *N*-methyl-4-(thieno[2,3-*d*]pyrimidin-4-ylamino) benzenesulfonamide **38** (85.6 mg, 0.267 mmol, 61%) as a pale-green solid. MS (ESI+) *m*/*z* calcd for C_13_H_13_N_4_O_2_S_2_^+^ [M + H]^+^ 321.0, found 321.3. HRMS (ESI+) *m*/*z* calcd for C_13_H_13_N_4_O_2_S_2_^+^ 321.0474 [M + H]^+^, found 321.0474. UPLC (method D) *t*_R_ = 3.66 min, >98%. ^1^H NMR (300 MHz, DMSO-*d*_6_) δ 10.03 (s, 1H), 8.61 (s, 1H), 8.20–8.09 (m, 2H), 7.97 (d, *J* = 6.1 Hz, 1H), 7.85–7.74 (m, 3H), 7.41–7.28 (m, 1H), 2.43 (d, *J* = 5.1 Hz, 3H). ^13^C NMR (75 MHz, DMSO) δ 167.42, 154.81, 153.37, 143.58, 133.08, 128.07, 125.08, 120.90, 119.98, 117.85, 29.17.

##### *N*-(4-Morpholinosulfonylphenyl)thieno[2,3-*d*]pyrimidin-4-amine (**39**)

4-Chlorothieno[2,3-*d*]pyrimidine (75.0 mg, 0.440 mmol) and 4-morpholinosulfonylaniline (107 mg, 0.440 mmol) were reacted according to general procedure 2 to give *N*-(4-morpholinosulfonylphenyl) thieno[2,3-*d*]pyrimidin-4-amine **39** (135 mg, 0.59 mmol, 82%) as a pale-green solid. MS (ESI+) *m*/*z* calcd for C_16_H_17_N_4_O_3_S_2_^+^ [M + H]^+^ 377.1, found 377.3. UPLC (method D) *t*_R_ = 4.23 min, 99%. ^1^H NMR (300 MHz, DMSO-*d*_6_) δ 10.21 (s, 1H), 8.65 (s, 1H), 8.30–8.18 (m, 2H), 8.03 (d, *J* = 6.0 Hz, 1H), 7.83 (d, *J* = 6.0 Hz, 1H), 7.80–7.69 (m, 2H), 3.66–3.62 (m, 4H), 2.93–2.83 (m, 4H).

##### 6-Chlorothieno[2,3-*d*]pyrimidin-4-ol (**50**)

Thieno[2,3-*d*]pyrimidin-4-ol (2.00 g, 13.14 mmol) was taken up in acetic acid (50 mL) and 1-chloropyrrolidine-2,5-dione (1.76 g, 13.1 mmol) added. The reaction was heated at 90 °C overnight. The reaction was cooled to rt and the resulting precipitate filtered and washed with water (×4) to give 6-chlorothieno[2,3-*d*]pyrimidin-4-ol **50** (1.30 g, 6.97 mmol, 53%) as a gray solid. MS (ESI+) *m*/*z* calcd for C_6_H_3_ClN_2_OS^+^ [M + H]^+^ 187.0, found 187.0. UPLC (method C) *t*_R_ = 2.32 min, 97%. ^1^H NMR (300 MHz, DMSO-*d*_6_) δ 12.69 (brs, 1H), 8.17 (d, *J* = 2.6 Hz, 1H), 7.45 (s, 1H).

##### 4,6-Dichlorothieno[2,3-*d*]pyrimidine (**51**)

6-Chlorothieno[2,3-*d*]pyrimidin-4-ol **50** (1.25 g, 6.7 mmol) was taken up in toluene (50 mL), cooled on ice, and phosphorus oxychloride (6.26 mL, 67.0 mmol) was added. The mixture was heated to 100 °C for 2 h. The reaction mixture was cooled and added slowly to ice-cold aq NaHCO_3_ solution, left to quench on ice for a further 30 min, then extracted with EtOAc. The organic layer was washed with satd. aq NaHCO_3_ (×2) and brine (×1), dried (Na_2_SO_4_) and solvent removed in vacuo to yield 4,6-dichlorothieno[2,3-*d*]pyrimidine **51** (1.32 g, 6.44 mmol, 96%) as a light-brown solid. MS (ESI+) *m*/*z* calcd for C_6_H_2_Cl_2_N_2_S^+^ [M + H]^+^ 204.9, found 204.9. UPLC (method C) *t*_R_ = 3.08 min, 89%. ^1^H NMR (300 MHz, DMSO-*d*_6_) δ 8.97 (s, 1H), 7.79 (s, 1H).

##### 6-Chloro-*N*-(4-methylsulfonylphenyl)thieno[2,3-*d*]pyrimidin-4-amine (**40**)

4,6-Dichlorothieno[2,3-*d*]pyrimidine **51** (75.0 mg, 0.370 mmol) and 4-(methylsulfonyl) aniline (62.6 mg, 0.370 mmol) were reacted according to general procedure 3 for 2 h at 125 °C. Upon cooling to rt the reaction mixture was loaded onto an SCX-II column, washed with MeOH, then eluted with 0.5 M NH_3_ in MeOH and concentrated in vacuo. Purification via preparatory HPLC (gradient elution 30–70% MeCN in H_2_O with 0.1% NH_3_) yielded 6-chloro-*N*-(4-methylsulfonylphenyl) thieno[2,3-*d*]pyrimidin-4-amine **40** (16.2 mg, 0.048 mmol, 13%) as a white solid. MS (ESI+) *m*/*z* calcd for C_13_H_11_ClN_3_O_2_S_2_^+^ [M + H]^+^ 340.0, found 340.0. HRMS (ESI+) *m*/*z* calcd for C_13_H_11_ClN_3_O_2_S_2_^+^ 339.9976 [M + H]^+^, found 339.9974. UPLC (method F) *t*_R_ = 4.66 min, >98%. ^1^H NMR (300 MHz, DMSO-*d*_6_) δ 9.99 (s, 1H), 8.63 (s, 1H), 8.19–8.08 (m, 2H), 8.02 (s, 1H), 7.97–7.89 (m, 2H), 3.20 (s, 3H). ^13^C NMR (75 MHz, DMSO) δ 166.10, 153.98, 153.63, 144.30, 134.81, 128.52, 127.86, 120.87, 119.64, 117.80, 44.35.

##### 4-Chlorothieno[2,3-*d*]pyrimidine-6-carbaldehyde (**52**)

*N*,*N*-Diisopropylamine (0.90 mL, 6.45 mmol) was dissolved in anhydrous THF (15 mL) under N_2_ and cooled to 0 °C. *n*-Butyllithium (2.58 mL, 6.45 mmol) was added dropwise, and after 10 min, the mixture was cooled to −78 °C when a solution of 4-chlorothieno[2,3-*d*]pyrimidine (1.00 g, 5.86 mmol) in anhydrous THF (5 mL) was added dropwise. The reaction was left at −78 °C for 1 h, when a solution of *N*,*N*-dimethylformamide (0.54 mL, 7.03 mmol) was added dropwise. The reaction was stirred for 15 min at −78 °C. The reaction was quenched with satd. aq NH_4_Cl, EtOAc was added and the layers separated. The organic layer was washed with brine, dried (Na_2_SO_4_), and solvent removed. The resulting solid was triturated with DCM followed by purification via silica gel chromatography (gradient elution 0–100% EtOAc in petroleum ether) to yield 4-chlorothieno[2,3-*d*]pyrimidine-6-carbaldehyde **52** (0.81 g, 4.076 mmol, 70%) as a yellow solid. MS (ESI+) *m*/*z* calcd for C_7_H_3_ClN_2_OS^+^ [M + H]^+^ 199.0, found no observed mass ion. UPLC (method A) *t*_R_ = 2.38 min, >98%. ^1^H NMR (300 MHz, chloroform-*d*) δ 10.18 (s, 1H), 9.01 (s, 1H), 8.16 (s, 1H).

##### 4-Chlorothieno[2,3-*d*]pyrimidine-6-carbonitrile (**53**)

4-Chlorothieno[2,3-*d*]pyrimidine-6-carbaldehyde **52** (0.6 g, 3.02 mmol) and ethyl *N*-{[(2,4,6-trimethylbenzene)sulfonyl]oxy}ethanecarboximidate (948.18 mg, 3.32 mmol) were taken up in DCM (30 mL) and trifluoromethanesulfonic acid (0.02 mL, 0.230 mmol) added. The reaction was left to stir for 4 days at rt. The reaction mixture was washed with H_2_O ×2, dried (MgSO_4_), and solvent removed. Purification via silica gel chromatography (gradient elution 0–10% to 50% EtOAc in petroleum ether) yielded 4-chlorothieno[2,3-*d*]pyrimidine-6-carbonitrile **53** (266 mg, 1.41 mmol, 47%) as a cream solid. MS (ESI+) *m*/*z* calcd for C_7_H_2_ClN_3_S^+^ [M + H]^+^ 196.0, found no observed mass ion. UPLC (method A) *t*_R_ = 2.53 min, 98%. ^1^H NMR (300 MHz, chloroform-*d*) δ 8.95 (s, 1H), 7.95 (s, 1H).

##### 4-(4-Methylsulfonylanilino)thieno[2,3-*d*]pyrimidine-6-carbonitrile (**41**)

4-Chlorothieno[2,3-*d*]pyrimidine-6-carbonitrile **53** (50.0 mg, 0.260 mmol) and 4-(methylsulfonyl) aniline (43.8 mg, 0.260 mmol) were reacted according to general procedure 3 for 2 h at 125 °C. After cooling, the resulting solid was filtered and washed with DCM. Purification via preparatory HPLC (gradient elution 20–60% MeCN in H_2_O with 0.1% NH_3_) yielded 4-(4-methylsulfonylanilino)thieno[2,3-*d*]pyrimidine-6-carbonitrile **41** (32.2 mg, 0.097 mmol, 38%) as a beige solid. MS (ESI+) *m*/*z* calcd for C_14_H_11_N_4_O_2_S_2_^+^ [M + H]^+^ 331.0, found 331.1. HRMS (ESI+) *m*/*z* calcd for C_14_H_11_N_4_O_2_S_2_^+^ 331.0318 [M + H]^+^, found 331.0320. UPLC (method D) *t*_R_ = 4.00 min, 99%. ^1^H NMR (300 MHz, DMSO-*d*_6_) δ 8.63 (s, 1H), 8.63 (s, 1H), 8.06–7.97 (m, 2H), 7.97–7.84 (m, 2H), 3.20 (s, 3H).

##### Methyl 2-Amino-5-(trifluoromethyl)thiophene-3-carboxylate (**54**)

Methyl 2-aminothiophene-3-carboxylate (1.00 g, 6.36 mmol) and tris(BPY) ruthenium(II) dichloride hexahydrate (47.6 mg, 0.060 mmol) were sealed in a vial, evacuated of air, and backfilled with N_2_. To this MeCN (2.5 mL) was added and the mixture stirred at rt before addition of TMEDA (1.9 mL, 12.72 mmol), followed by 5 min stirring before gaseous trifluoromethyl iodide (3.74 g, 19.09 mmol) was added and the reaction left to stir for 60 h with an LED light attached. After this time, the solvent was removed under reduced pressure and crude material purified via silica gel chromatography (gradient elution 2–50% EtOAc in petroleum ether) to yield methyl 2-amino-5-(trifluoromethyl) thiophene-3-carboxylate **54** (850 mg, 3.77 mmol, 59% yield). ^1^H NMR (300 MHz, chloroform-*d*) δ 7.36 (q, *J* = 1.4 Hz, 1H), 6.21 (s, 2H) and 3.83 (s, 3H). ^19^F NMR (282 MHz, chloroform-d) δ −55.75.

##### 6-(Trifluoromethyl)-3*H*-thieno[2,3-*d*]pyrimidin-4-one (**55**)

Methyl 2-amino-5-(trifluoromethyl) thiophene-3-carboxylate **54** (850 mg, 3.77 mol) and formamidine acetate (2.25 g, 50.96 mmol) were taken up in IPA (3 mL) and heated at 120 °C for 24 h. After this time, no SM was identified by LCMS analysis, so reaction poured into ice and stirred for 30 min, before filtering and yielding 6-(trifluoromethyl)-3*H*-thieno[2,3-*d*]pyrimidin-4-one **55** (550 mg, 2.5 mmol, 74% yield) as a gray solid. Product carried forward without further purification.

##### 4-Chloro-6-(trifluoromethyl)thieno[2,3-*d*]pyrimidine (**56**)

6-(Trifluoromethyl)-3*H*-thieno[2,3-*d*]pyrimidin-4-one **55** (129 mg, 0.590 mmol) was taken up in phosphorus oxychloride (1.11 mL, 11.9 mmol), and the reaction mixture was heated for 1 h at 100 °C. After this time, LCMS analysis showed no remaining starting material, so the solvent was removed under reduced pressure, crude material taken up in DCM, and the solvent removed again ×2, and finally once with toluene. The resulting product 4-chloro-6-(trifluoromethyl) thieno[2,3-*d*]pyrimidine **56** was taken forward without further purification.

##### *N*-(4-Methylsulfonylphenyl)-6-(trifluoromethyl)thieno[2,3-*d*]pyrimidin-4-amine (**42**)

4-Chloro-6-(trifluoromethyl) thieno[2,3-*d*]pyrimidine **56** (90.0 mg, 0.380 mmol) was taken up in IPA (2 mL) in a μW vial, and to this was added 4-(methylsulfonyl) aniline (129 mg, 0.750 mmol). The mixture was heated under μW irradiation at 100 °C for 2 h. The reaction mixture was then concentrated under reduced pressure to give a thick oil, which was dry loaded on silica for initial purification via silica gel chromatography (gradient elution 0–10% MeOH in DCM). Impurities were still visible so it further purified by preparatory HPLC (gradient elution 5–95% MeCN in H_2_O with 0.1% NH_3_) to yield *N*-(4-methylsulfonylphenyl)-6-(trifluoromethyl)thieno[2,3-*d*]pyrimidin-4-amine **42** (88.0 mg, 0.236 mmol, 40% yield) as an off-white solid. MS (ESI−) for C_14_H_10_F_3_N_3_O_2_S_2_ [M – H]^−^ 373.0, found 372.0. HRMS (ESI+) *m*/*z* calcd for C_14_H_10_F_3_N_3_O_2_S_2_^+^ 374.0239 [M + H]^+^, found 374.0240. UPLC analysis (method B), 5.08 min, >95%. ^1^H NMR (300 MHz, DMSO-*d*_6_) δ 10.33 (s, 1H), 8.75 (s, 1H), 8.63 (t, *J* = 1.4 Hz, 1H), 8.21–8.11 (m, 2H), 8.02–7.91 (m, 2H), 3.22 (s, 3H). ^13^C NMR (75 MHz, DMSO) δ 168.14, 155.97, 155.86, 143.91, 135.28, 128.56, 125.36 (q, *J* = 38.1 Hz, *C*CF_3_), 123.92 (q, *J* = 4.4 Hz, *C*HCCF_3_), 122.77 (d, *J* = 269.3 Hz, *C*F_3_), 121.20, 116.71, 44.31. ^19^F NMR (282 MHz, DMSO-*d*_6_) δ −55.25 (d, *J* = 1.1 Hz, CF_3_).

##### *N*-(4-Methylsulfonylphenyl)-6-phenyl-thieno[2,3-*d*]pyrimidin-4-amine (**43**)

4-(Methylsulfonyl) aniline hydrochloride (100 mg, 0.484 mmol) and 4-chloro-6-phenylthieno[2,3-*d*]pyrimidine (75.0 mg, 0.440 mmol) were reacted according to general procedure 1. The reaction was cooled to rt and 0.5 M NH_3_ in MeOH (20 mL) added and solvent removed in vacuo. Purification via preparatory HPLC (gradient elution 10–50% MeCN in H_2_O with 0.1% NH_3_) yielded *N*-(4-methylsulfonylphenyl)-6-phenyl-thieno[2,3-*d*]pyrimidin-4-amine **43** (43.6 mg, 0.114 mmol, 26%) as a white solid. MS (ESI+) *m*/*z* calcd for C_19_H_16_N_3_O_2_S_2_^+^ 382.1 [M + H]^+^, found 382.1. HRMS (ESI+) *m*/*z* calcd for C_19_H_16_N_3_O_2_S_2_^+^ 382.0684 [M + H]^+^, found 382.0683. UPLC analysis (method D), 5.28 min, >98%. ^1^H NMR (300 MHz, chloroform-*d*) δ 8.71 (s, 1H), 8.08–7.91 (m, 4H), 7.79–7.68 (m, 2H), 7.59–7.38 (m, 4H), 7.35 (s, 1H), 3.11 (s, 3H). ^13^C NMR (75 MHz, DMSO) δ 166.87, 154.36, 153.55, 144.56, 140.46, 134.58, 133.29, 130.00, 129.56, 128.54, 126.41, 120.70, 119.36, 115.64, 44.37.

##### Methyl 4-Chlorothieno[2,3-*d*]pyrimidine-6-carboxylate (**57**)

4,6-Dichloro-5-pyrimidinecarbaldehyde (1.0 g, 5.65 mmol) was dissolved in DCM (40 mL) with DIPEA (0.98 mL, 5.65 mmol) and cooled to −80 °C. Methyl sulfanylacetate (0.5 mL, 5.65 mmol) in DCM (40 mL) was added dropwise over 30 min and the reaction left to warm to rt overnight. Washed with water ×3 and brine ×1, dried (MgSO_4_), and solvent removed to give an orange oil that solidified on standing. The solid was taken back up in IPA and DIPEA (2 mL) and heated to 85 °C overnight. Reaction mixture was cooled to rt and solvent removed in vacuo. Purification via silica gel chromatography (gradient elution 0–50% EtOAc in petroleum ether) yielded methyl 4-chlorothieno[2,3-*d*]pyrimidine-6-carboxylate **57** (1.09 g, 4.77 mmol, 84%). MS (ESI+) *m*/*z* calcd for C_8_H_5_ClN_2_O_2_S^+^ [M + H]^+^ 229.0, found no observed mass ion. UPLC (method A) *t*_R_ = 2.77 min. ^1^H NMR (300 MHz, CDCl_3_) δ 8.97 (s, 1H), 8.16 (s, 1H), 4.03 (s, 3H).

##### Methyl 4-(4-Methylsulfonylanilino)thieno[2,3-*d*]pyrimidine-6-carboxylate (**58**)

Methyl 4-chlorothieno[2,3-*d*]pyrimidine-6-carboxylate **57** (0.5 g, 2.19 mmol) and 4-(methylsulfonyl) aniline (0.37 g, 2.19 mmol) were reacted according to general procedure 3 for 2 h at 125 °C. Upon cooling, the reaction mixture was loaded onto an SCX-II column and washed through with a large volume of MeOH. A solid persisted on top of the column, which was recovered and dried under high vacuum. Further product was eluted from the column with 0.5 M NH_3_ in MeOH. Solvent removed in vacuo and the resulting solid triturated with MeOH (×3). Batches combined to give methyl 4-(4-methylsulfonylanilino)thieno[2,3-*d*]pyrimidine-6-carboxylate **58** (0.406 g, 1.12 mmol, 51%) as a pale-yellow solid. MS (ESI+) *m*/*z* calcd for C_15_H_13_N_3_O_4_S_2_^+^ [M + H]^+^ 364.0, found 364.1. UPLC (method F) *t*_R_ = 4.29 min, 98%. ^1^H NMR (300 MHz, DMSO-*d*_6_) δ 10.35 (s, 1H), 8.81 (s, 1H), 8.72 (s, 1H), 8.27–8.13 (m, 2H), 8.01–7.89 (m, 2H), 3.94 (s, 3H), 3.21 (s, 3H).

##### 4-(4-Methylsulfonylanilino)thieno[2,3-*d*]pyrimidine-6-carboxylic Acid (**59**)

Methyl 4-(4-methylsulfonylanilino) thieno[2,3-*d*]pyrimidine-6-carboxylate **58** (0.40 g, 1.1 mmol) was taken up in MeOH/THF (1:1) (6 mL) and lithium hydroxide (0.23 g, 5.5 mmol) in H_2_O (2 mL) was added. The suspension was stirred at rt overnight. The reaction mixture was acidified to pH ∼4 with 1 M HCl and the resulting “clay-like” white solid filtered and washed with H_2_O. The solid was suspended in water and MeCN and lyophilized to give 4-(4-methylsulfonylanilino) thieno[2,3-*d*]pyrimidine-6-carboxylic acid **59** (0.383 g, 1.10 mmol, 100%) as a white solid. MS (ESI+) *m*/*z* calcd for C_14_H_12_N_3_O_4_S_2_^+^ [M + H]^+^ 350.0, found 350.1. UPLC (method F) *t*_R_ = 3.40 min, 100%. ^1^H NMR (300 MHz, DMSO-*d*_6_) δ 13.78 (br s, 1H), 10.30 (s, 1H), 8.712 (s, 1H), 8.710 (s, 1H), 8.25–8.14 (m, 2H), 8.00–7.89 (m, 2H), 3.21 (s, 3H).

##### *N*,*N*-Dimethyl-4-(4-methylsulfonylanilino)thieno[2,3-*d*]pyrimidine-6-carboxamide (**60**)

4-(4-Methylsulfonylanilino) thieno[2,3-*d*]pyrimidine-6-carboxylic acid **59** (50.0 mg, 0.140 mmol) was dissolved in DMF (2 mL) and dimethylamine (0.07 mL, 0.140 mmol) was added with HATU (54.4 mg, 0.140 mmol) and DIPEA (0.02 mL, 0.140 mmol). The mixture was left to stir overnight at rt. Upon completion, the reaction mixture was diluted with EtOAc, washed with satd. NaHCO_3_ ×1 and brine ×2, then dried (Na_2_SO_4_), filtered, and concentrated in vacuo to give *N*,*N*-dimethyl-4-(4-methylsulfonylanilino)thieno[2,3-*d*]pyrimidine-6-carboxamide **60** (54 mg, 0.143 mmol, 100%) as a beige solid. MS (ESI+) *m*/*z* calcd for C_16_H_16_N_4_O_3_S_2_^+^ [M + H]^+^ 377.1, found 377.2. UPLC (method A) *t*_R_ = 2.41 min, >98%.

##### 6-[(Dimethylamino) Methyl]-*N*-(4-methylsulfonylphenyl)thieno[2,3-*d*]pyrimidin-4-amine (**44**)

*N*,*N*-Dimethyl-4-(4-methylsulfonylanilino)thieno[2,3-*d*]pyrimidine-6-carboxamide **60** (54.0 mg, 0.150 mmol) was dissolved in THF (2.12 mL) and cooled to 0 °C when LiAlH_4_ (1 M in THF, 0.15 mL, 0.150 mmol) was added dropwise. The reaction was left to stir at 0 °C for 1 h. Then satd. aq NaHCO_3_ was added and extracted with EtOAc (×2), the combined organic layers were washed with brine (×1), then dried (Na_2_SO_4_), filtered, and concentrated in vacuo (45 mg crude). Purification via preparatory HPLC (gradient elution 20–60% MeCN in H_2_O with 0.1% NH_3_) yielded 6-[(dimethylamino)methyl]-*N*-(4-methylsulfonylphenyl)thieno[2,3-*d*]pyrimidin-4-amine **44** (27.7 mg, 0.076 mmol, 52%) as a cream solid. MS (ESI+) *m*/*z* calcd for C_16_H_19_N_4_O_2_S_2_^+^ [M + H]^+^ 363.1, found 363.3. UPLC (method D) *t*_R_ = 3.86 min, 98%. ^1^H NMR (300 MHz, DMSO-*d*_6_) δ 9.95 (s, 1H), 8.58 (s, 1H), 8.23–8.11 (m, 2H), 7.97–7.85 (m, 2H), 7.80 (d, *J* = 1.1 Hz, 1H), 3.77–3.70 (m, 2H), 3.19 (s, 3H), 2.26 (s, 6H).

##### 1-[4-[(6-Methylthieno[2,3-*d*]pyrimidin-4-yl)amino]phenyl]pyrrolidin-2-one (**45**)

4-Chloro-6-methylthieno[2,3-*d*]pyrimidine (50.0 mg, 0.270 mmol) and 1-(4-aminophenyl)-2-pyrrolidinone (47.7 mg, 0.270 mmol) were reacted according to general procedure 4. Upon cooling to rt, the reaction mixture was loaded onto an SCX-II column, washed with MeOH, then eluted with 0.5 M NH_3_ in MeOH and concentrated in vacuo. Purification via preparatory HPLC (gradient elution 20–60% MeCN in H_2_O with 0.1% NH_3_) yielded 1-[4-[(6-methylthieno[2,3-*d*]pyrimidin-4-yl)amino]phenyl]pyrrolidin-2-one **45** (12.0 mg, 0.037 mmol, 14%) as a white solid. MS (ESI+) *m*/*z* calcd for C_17_H_16_N_4_OS^+^ [M + H]^+^ 325.1, found 325.1. UPLC (method D) *t*_R_ = 4.06 min, 100%. ^1^H NMR (300 MHz, DMSO-*d*_6_) δ 9.50 (s, 1H), 8.41 (s, 1H), 7.86–7.75 (m, 2H), 7.71–7.59 (m, 2H), 7.55 (d, J = 1.3 Hz, 1H), 3.84 (t, J = 7.0 Hz, 2H), 2.59 (d, J = 1.2 Hz, 3H), 2.52–2.46 (m, 2H, obs. by DMSO peak), 2.15–1.99 (m, 2H).

##### *N*-(4-(Cyclopentylsulfonyl)phenyl)-6-methylthieno[2,3-*d*]pyrimidin-4-amine (**46**)

A dry 5 mL μW flask was charged with 4-chloro-6-methylthieno[2,3-*d*]pyrimidine (50.0 mg, 0.270 mmol), 4-(cyclopentanesulfonyl) aniline (67.1 mg, 0.300 mmol), Xantphos (15.7 mg, 0.030 mmol) and cesium carbonate (176 mg, 0.540 mmol), then toluene (3 mL) was added and the reaction degassed by passing N_2_ through for 5 min. Then tris(dibenzylideneacetone) dipalladium(0) chloroform adduct (14.0 mg, 0.010 mmol) was added, the reaction mixture degassed again, and reaction was heated under microwave irradiation at 120 °C for 1.5 h. Upon completion the mixture was loaded onto an SCX-2 column and washed through with MeOH. The product was eluted with 0.5 M NH_3_ in MeOH and concentrated in vacuo. Purification via preparative HPLC (gradient elution 5–95% MeCN in H_2_O with 0.1% NH_3_) followed by purification via silica gel chromatography (gradient elution 8–60% EtOAc in petroleum ether) yielded *N*-(4-(cyclopentylsulfonyl) phenyl)-6-methylthieno[2,3-*d*]pyrimidin-4-amine **46** (15.0 mg, 0.040 mmol, 14.8% yield) as a white solid. MS (ESI+) *m*/*z* calcd for C_18_H_20_N_3_O_2_S_2_^+^ [M + H]^+^ 374.5, found 374.2. UPLC analysis (method E), 5.17 min, >98%. ^1^H NMR (300 MHz, chloroform-*d*) δ 8.67 (s, 1H), 8.02–7.84 (m, 4H), 7.18 (s, 1H), 7.03 (q, *J* = 1.2 Hz, 1H), 3.61–3.44 (m, 1H), 2.66 (d, *J* = 1.2 Hz, 3H), 2.20–2.02 (m, 2H), 1.99–1.85 (m, 2H), 1.85–1.72 (m, 2H), 1.71–1.60 (m, 2H).

## Supporting Information Available

Supplementary tables and figures as well as further experimental details (biophysical experiments, X-ray parameters, selectivity screening data, chemical synthesis schemes, ChEMBL similarity searches, selected NMR and HPLC spectra) (PDF)
Molecular formula strings (CSV)

## Accession Codes

8BQ4: Authors will release the atomic coordinates upon article publication.

## Abbreviations Used

ADMET: absorption, distribution, metabolism, excretion and toxicity
aq: aqueous
ATP: adenosine triphosphate
BPY: 2,2′-bipyridine
DCM: dichloromethane
DIPEA: *N*,*N*-diisopropylethylamine
DMF: *N*,*N*-dimethylformamide
DMSO: dimethyl sulfoxide
DSF: differential scanning fluorimetry
ER: efflux ratio
HATU: 1-[bis(dimethylamino) methylene]-1*H*-1,2,3-triazolo[4,5-*b*]pyridinium 3-oxid hexafluorophosphate
HPLC: high-performance liquid chromatography
HRMS: high-resolution mass spectra
IPA: isopropyl alcohol
LCMS: liquid chromatography–mass spectrometry
LE: ligand efficiency
LED: light-emitting diode
LLE: lipophilic ligand efficiency
MLM: mouse liver microsomes
MST: microscale thermophoresis
NCS: *N*-chlorosuccinimide
PI5P4K: phosphatidylinositol 5-phosphate 4-kinase
SAR: structure–activity relationship
TFA: trifluoroacetic acid
TfOH: trifluoromethanesulfonic acid
THF: tetrahydrofuran
TMEDA: tetramethylethylenediamine
TMS: tetramethylsilane
UPLC: ultraperformance liquid chromatography
WT: wild-type
Xantphos: 4,5-bis(diphenylphosphino)-9,9-dimethylxanthene
μW: microwave
