# Supplementary material for: The Identification of Potent, Selective, and Brain Penetrant PI5P4Kγ Inhibitors as In Vivo-Ready Tool Molecules
Source: J Med Chem. 2022 Dec 14;66(1):804–21. doi: 10.1021/acs.jmedchem.2c01693 (PMC9841522; doi:10.1021/acs.jmedchem.2c01693)

Supporting Information

The Identification of Potent, Selective, and Brain Penetrant PI5P4Kγ Inhibitors as In Vivo-Ready Tool Molecules

Timothy P. C. Rooney,‡ Gregory G. Aldred,‡ Helen K. Boffey,‡ Henriëtte M. G. Willems,‡ Simon Edwards, Stephen J. Chawner, Duncan E. Scott, Christopher Green, David Winpenny, John Skidmore, Jonathan H. Clarke, Stephen P. Andrews*

Affiliations

The ALBORADA Drug Discovery Institute, University of Cambridge, Island Research Building, Cambridge Biomedical Campus, Hills Road, Cambridge, CB2 0AH, United Kingdom

*corresponding author: spa26@cam.ac.uk

Table of Contents

	Page
Biophysical experiments	S2
X-ray crystallography data and kinase inhibitor similarity searching	S7
Kinase selectivity screening	S8
Cerep selectivity screening	S14
ADMET experimental methods	S16
UPLC method parameters	S17
Synthetic Schemes	S18
References for supporting information	S20
NMR spectra of compounds from Table 6	S21
HPLC traces of compounds from Table 6	S34

Biophysical Experiments

A selection of compounds from the series was validated biophysically against the PI5P4Kγ-WT protein both by microscale thermophoresis (MST) and differential scanning fluorimetry (DSF). In the MST experiments, PI5P4Kγ-WT protein was labelled with a suitable fluorophore and compounds were titrated against the labelled-protein. The MST experiments showed a good MST response in the presence of compounds that were active in the kinetic assay and no response in titrations of compounds inactive in the kinetic assay, Figure S1 and Figure S3. In particular, compound **1** and isomers **15** and **14,** which differ by a single methyl group, gave the expected MST responses (Figure S1). A good correlation between the potencies in the kinetic and MST experiments was obtained, suggesting minimal influence of the covalently attached fluorophore. A good correlation between MST pK_Ds and ADP-Glo pIC_{50}s (with PI5P4Kγ+) was also observed.

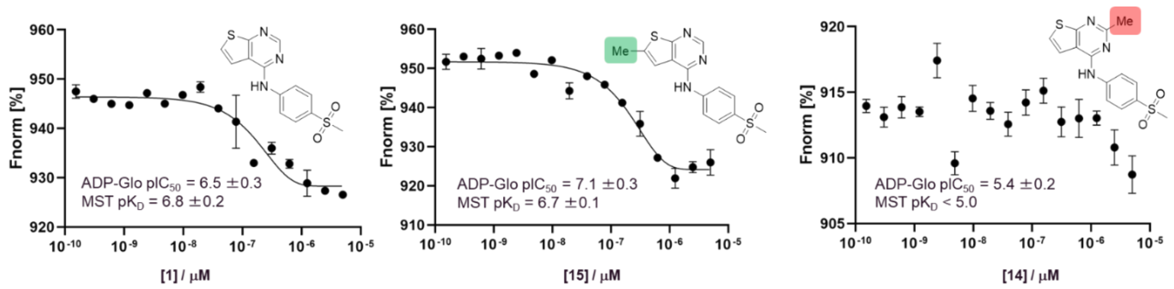

Figure S1. Sensitivity of the MST response to moving a methyl group is shown; MST activity is retained for active compound **15** versus **1**, which is not observed for inactive compound **14**.

An exemplar set of biophysical validation data is shown for compound **25** (Figure S2). In addition to MST data (Figure S2A-B), compound binding to PI5P4Kγ was further characterized using DSF as a label-free biophysical technique. The active compounds showed clear stabilisation of melting temperature (T_M) of the PI5P4Kγ-WT protein with increasing concentration of **25**, Figure S2C. Further, the raw DSF titration data was fitted according to Bai *et. al.*[1] (Figure S2D), to provide an estimation of the pK_D (7.3 ± 0.2), which was in good agreement with that obtained in both the kinetic assay and the MST experiment.

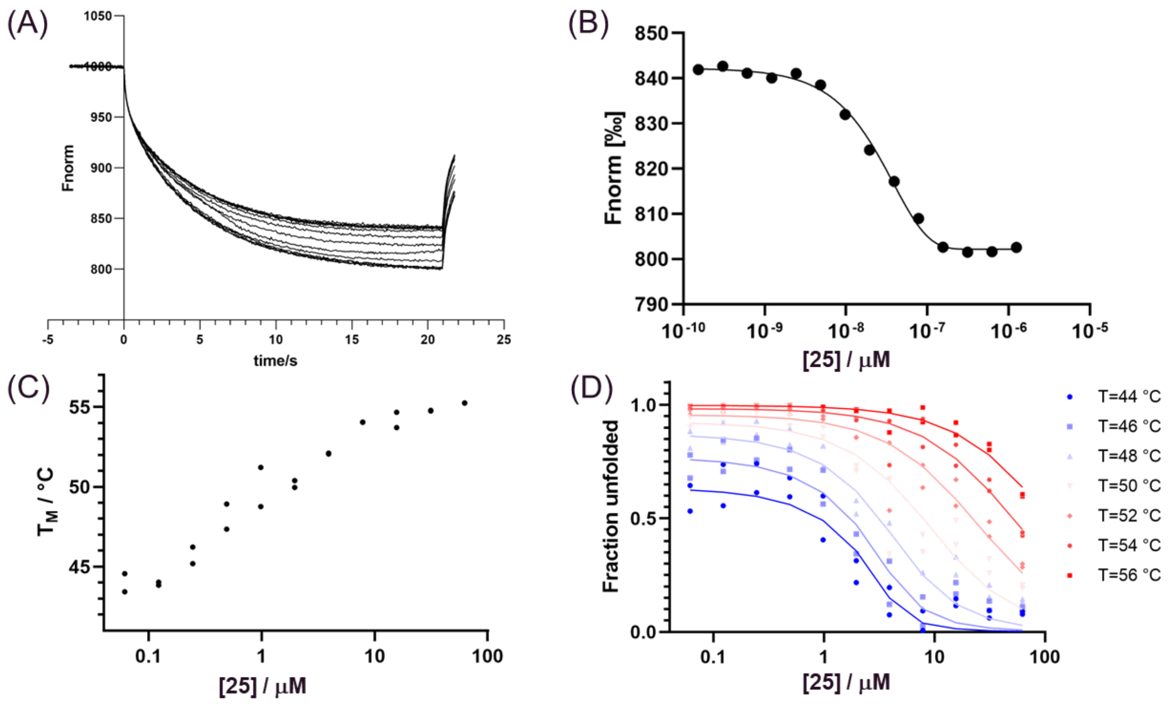

Figure S2. Biophysical validation of **25** binding to PI5P4Kγ-WT. (A) Raw MST response of labelled-protein in the presence of **25** and (B) the fitted data for the same titration. (C) The protein melting temperature in a DSF titration of **25** is plotted and (D) the raw data processed into an isothermal DSF plot, Bai *et. al*.[1]

MST experimental

Labelling PI5P4Kγ-WT for MST experiments

PI5P4Kγ-WT was labelled with a Monolith RED-NHS Protein Labelling Kit (NanoTemper, Cat# MO-L001) containing proprietary labelling buffer, buffer exchange column A and purification column B. Briefly, a solution of PI5P4Kγ-WT (212 µM) was diluted to 20 µM with labelling buffer and buffer exchanged into labelling buffer by centrifugation (1500 g for 2 minutes, column A). The buffer-exchanged protein solution (100 µL) was mixed with a solution of RED-NHS dye (63 µM, 100 µL) to give a 200 µL solution with approximately 3:1 dye to protein ratio. The solution was incubated at room temperature in the dark for 30 minutes. Purification column B was equilibrated with PBS (pH 7.4) and the unreacted dye separated from the protein by passing through column B and collecting fractions. The collected fractions were assessed by MST (NanoTemper Monolith NT.115, 20% LED power) by diluting 1 µL of each fraction 100-fold. Each fraction was also assessed by UV absorbance and

fractions were pooled that contained both significant absorbance at 280 and 650 nm. The resulting labelled protein stock PI5P4Kγ (4 µM) was divided into 10 µL aliquots and stored at -20 °C.

Performing MST experiments with labelled PI5P4Kγ-WT

An aliquot of labelled PI5P4Kγ-WT protein (4 µL at 4 µM) was diluted into 396 µL MST buffer (PBS pH 7.4 with 0.05% TWEEN-20) and spun down for 10 mins at 15,000 g at 4 °C. The supernatant (~390 µL) was collected and used in MST experiments. The protein solution (19 µL) was mixed with a DMSO solution of the compound (1 µL) to give the final protein concentration as 40 nM in MST buffer with 5% DMSO and equilibrated for 30 minutes at room temperature. The solution was transferred into a premium coated capillary (NanoTemper, Cat# MO-K005) and the MST signal measured with a NanoTemper Monolith NT.115 instrument running MO.Control v1.5.2 software. The temperature was controlled at 22 °C, the excitation power was between 20%-40% with either medium or high MST power for 20 seconds (see Table S1). The pre- and post-MST time was 4 seconds and 1 second respectively. The compounds were titrated in a two-fold dilution series across 16 capillaries from 5 µM to 0.153 nM. Titration data was fitted with NanoTemper software MO.Affinity Analysis v2.2.6 to obtain the K_D (n=2). Representative MST data for compounds **14** and **45** is shown in Figure S3.

Table S1. MST pK_Ds (PI5P4Kγ-WT) compared to ADP-Glo pIC_{50}s (PI5P4Kγ+)

**Compound**	**LED power**	**MST power**	**MST analysis range**	**PI5P4Kγ-WT MST pK_D**	**PI5P4Kγ+ ADP-Glo pIC_{50}**
**1**	20%	Medium	4.0-5.0 s	6.8 ± 0.2	6.5
**15**	40%	Medium	4.0-5.0 s	6.7 ± 0.1	7.0
**25**	20%	High	1.5-2.5 s	8.2 ± 0.2	7.7
**14**	30%	Medium	4.0-5.0 s	< 5.0	5.4
**45**	40%	Medium	4.0-5.0 s	6.2 ± 0.1	6.8

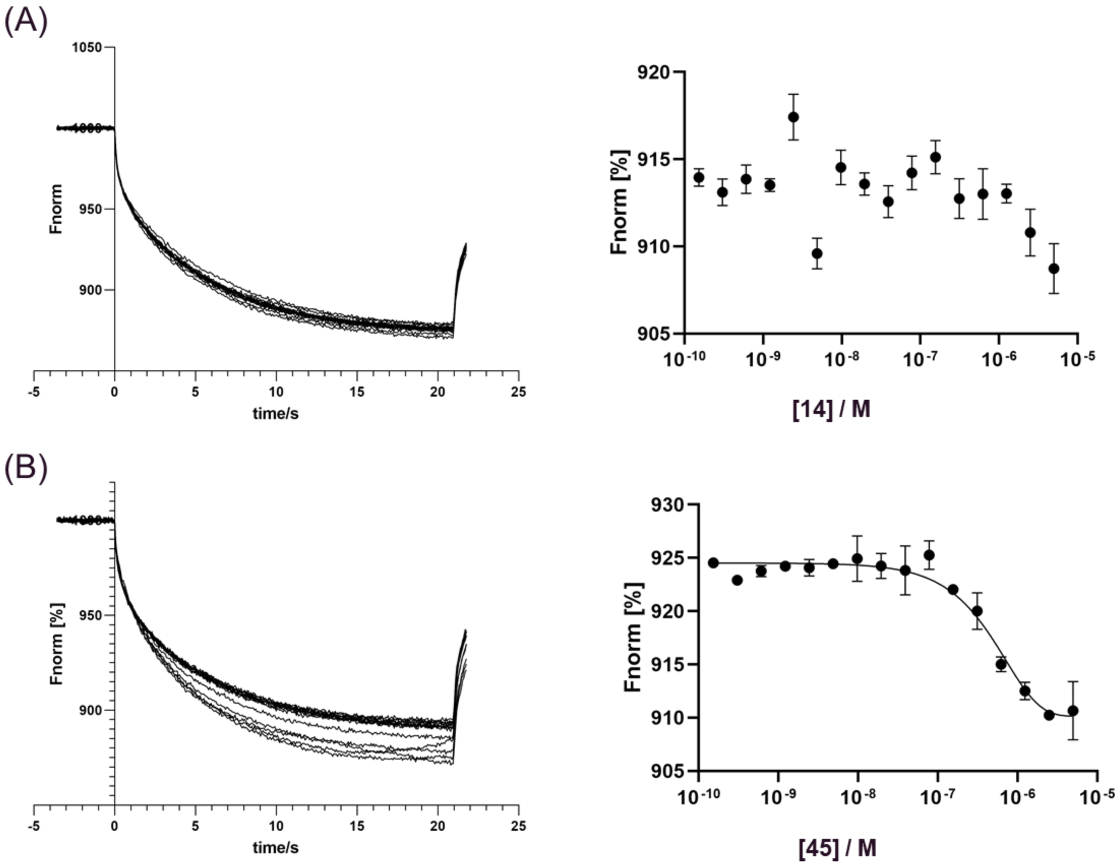

Figure S3. MST traces (left) and fitted data (right) for titrations of (A) inactive compound **14** and (B) active compound **45**.

Differential Scanning Fluorimetry titrations

The DSF experiments were performed under conditions previously reported by Boffey *et. al.*[2] Briefly, the thermal shift experiment was performed with an Applied Biosystem StepOne Real-Time PCR System in 96-well plates (Life technologies, cat. 4346906) sealed with optically clear lids (Life technologies, cat. 4360954). The final concentration of the wild-type PI5P4K-γ protein was 4 μM and the ligand was titrated in 2-fold dilutions from 62.5 μM to 0.061 μM (11 points) with DMSO-only controls. A 5000x stock solution of Sypro Orange was used (Life technologies, cat. S6650), at a final concentration of 5x. The buffer was 50 mM HEPES pH 7.4, 5 mM $MgCl_2$, and 100 mM NaCl with 5% DMSO in a total volume of 20 μL. The plates were heated from 25 to 90 °C at a rate of 0.5 °C/min. The raw data was fitted using the analysis python script 'DSF-fitting' reported by Bai *et. al.*[1] and the data plotted in Prism version 9.3.1. The isothermal DSF pK_D for compounds **1**, **25** and **45** were

calculated as 6.0 ± 0.1, 7.3 ± 0.2 and 6.1 ± 0.3 respectively. The DSF titrations for **1** and **45** are shown in Figure S4.

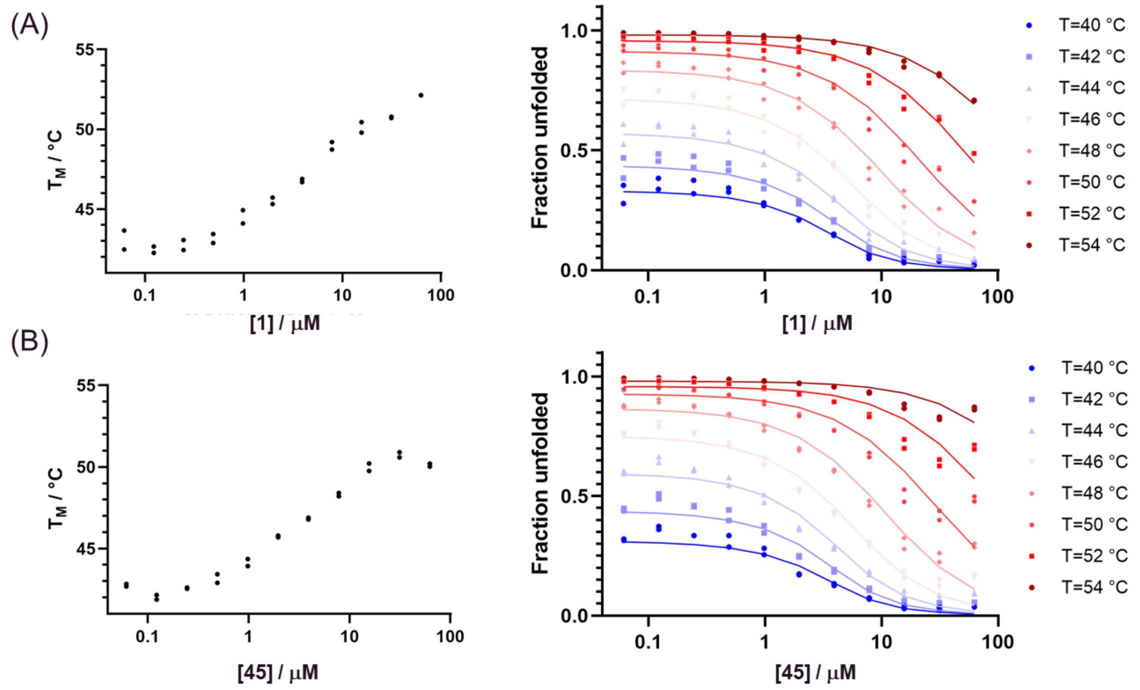

Figure S4. (left) PI5P4Kγ-WT melting temperature in DSF titrations and (right) raw data fitted to isothermal DSF plots (as in ref 1) for (A) **1** and (B) **45**.

Table S2. Data collection and refinement statistics for X-ray crystal structures of PI5P4Kγ bound to compound **15**.

PDB ID	8BQ4
Protein/Ligand	PI5P4Kγ/compound **15**
Wavelength [Å]	0.97624
Space group	P 2_12_12
Cell dimensions	
a; b; c; [Å]	120.01; 78.86; 96.97
α; β; γ; [◦]	90.0; 90.0; 90.0
Resolution [Å]	2.42 (2.46-2.42)[a]
Unique reflections	35432 (1773)[2]
Multiplicity	6.4 (6.5)[2]
Completeness [%]	98.8 (100)[2]
R_{sym} [%]	7.2(260)[2]
R_{meas} [%]	7.9 (238)[2]
Mean(I)/sd	11.4 (0.6)[2]
CC(1/2)	0.999 (0.403)
Number of reflections (free)	35005 (1752)
R_{cryst} [%]	22.2
R_{free}[%]	24.7
Total number of atoms:	
Protein	4744
Water	38
Ligand	42
Deviation from ideal geometry:	
Bond lengths [Å]	0.01
Bond angles [◦]	1.12

[a]values in parenthesis refer to the highest resolution bin.

Similarity Searching

The ChEMBL database was searched for compounds similar to the novel inhibitors reported in this manuscript and hits obtained were further examined for available kinase activity in the ChEMBL database. Substructure search for **15** and **1** did not yield any hits in the ChEMBL database. The chemotype was assessed more generally by performing a similarity search in ChEMBL for **15** and **1**. There were no similarity hits for compound **15** at >=40% similarity. There were 23 hits with 50-64% similarity to compound **1**. Three of these hits had reported kinase activity. CHEMBL355983 was reported to show a pIC_{50} of 7.5 in an EGFR inhibition assay.[3] ChEMBL3628791 was reported to show a pIC_{50} of 5.5 in an EGFR inhibition assay and to be inactive against KDR.[4] CHEMBL3628798 was tested in 9 kinase assays, and shown to inhibit KIT with a pIC_{50} of 9, KDR, FLT4, PDGFRB and RAF1 with pIC_{50}'s between 5 and 6, and was inactive against EGFR, MET, SRC and FLT1.[4] The 3 hits contain the N-phenylbenzo[b]thiophen-4-amine core, but not the sulfone substituent. Searches were performed in ChEMBL version 31 in November 2022.

Table S3. Kinase selectivity screening for compound **15** at 10 μM against a general kinase panel of 140 targets in radiometric filter binding assay using 33P-g-ATPat the MRC PPU International Centre for Kinase Profiling, University of Dundee.

**Kinase**	**% activity remaining**	**s.d.**
MKK1	**108**	14
MKK2	**95**	21
MKK6	**109**	2
ERK1	**104**	13
ERK2	**113**	8
ERK5	**97**	3
JNK1	**118**	12
JNK2	**101**	4
JNK3	**95**	5
p38a MAPK	**106**	11
p38b MAPK	**96**	10
p38g MAPK	**102**	4
p38d MAPK	**98**	4
ERK8	**90**	13
RSK1	**106**	0
RSK2	**104**	7
PDK1	**114**	7
PKBa	**100**	2
PKBb	**95**	3
SGK1	**68**	4
S6K1	**94**	21
PKA	**123**	1
ROCK 2	**128**	12
PRK2	**99**	2
PKCa	**112**	5
PKCγ	**124**	20
PKCz	**103**	3
PKD1	**107**	0
STK33	**95**	9

MSK1	**91**	9
MNK1	**91**	4
MNK2	**80**	0
MAPKAP-K2	**107**	6
MAPKAP-K3	**93**	22
PRAK	**111**	17
CAMKKb	**101**	4
CAMK1	**109**	5
SmMLCK	**106**	8
PHK	**93**	2
DAPK1	**107**	4
CHK1	**99**	11
CHK2	**83**	24
GSK3b	**124**	7
CDK2-Cyclin A	**75**	3
CDK9-Cyclin T1	**100**	17
PLK1	**106**	3
Aurora A	**113**	7
Aurora B	**31**	0
TLK1	**121**	21
LKB1	**119**	4
AMPK (hum)	**91**	3
MARK1	**124**	18
MARK2	**106**	8
MARK3	**100**	27
MARK4	**109**	6
BRSK1	**97**	7
BRSK2	**104**	1
MELK	**101**	7
NUAK1	**103**	9
SIK2	**103**	7
SIK3	**95**	1
TSSK1	**95**	3

CK1γ2	**94**	7
CK1δ	**103**	2
CK2	**103**	1
TTBK1	**106**	11
TTBK2	**124**	19
DYRK1A	**96**	3
DYRK2	**80**	5
DYRK3	**101**	9
NEK2a	**91**	6
NEK6	**107**	1
IKKb	**106**	5
IKKe	**104**	15
TBK1	**100**	0
PIM1	**120**	7
PIM2	**110**	0
PIM3	**66**	2
SRPK1	**106**	4
EF2K	**108**	2
EIF2AK3	**102**	6
HIPK1	**106**	7
HIPK2	**101**	3
HIPK3	**106**	9
CLK2	**37**	1
PAK2	**124**	0
PAK4	**110**	0
PAK5	**116**	6
PAK6	**94**	18
MST2	**108**	8
MST3	**107**	2
MST4	**103**	22
GCK	**103**	13
MAP4K3	**108**	5
MAP4K5	**101**	0

MINK1	**86**	13
MEKK1	**101**	9
MLK1	**97**	8
MLK3	**93**	1
TESK1	**96**	2
TAO1	**111**	9
ASK1	**112**	22
TAK1	**109**	0
IRAK1	**86**	6
IRAK4	**99**	2
RIPK2	**74**	1
OSR1	**115**	2
TTK	**88**	6
MPSK1	**106**	4
WNK1	**107**	7
ULK1	**107**	2
ULK2	**128**	10
TGFBR1	**109**	11
Src	**108**	8
Lck	**102**	3
CSK	**91**	7
YES1	**111**	12
ABL	**98**	4
BTK	**111**	2
JAK3	**103**	13
SYK	**91**	4
ZAP70	**101**	9
TIE2	**144**	56
BRK	**90**	18
EPH-A2	**98**	10
EPH-A4	**125**	21
EPH-B1	**107**	1
EPH-B2	**101**	13

EPH-B3	**86**	18
EPH-B4	**112**	4
FGF-R1	**109**	39
HER4	**99**	11
IGF-1R	**124**	4
IR	**107**	11
IRR	**101**	15
TrkA	**83**	21
DDR2	**127**	0
VEG-FR	**100**	3
PDGFRA	**99**	14
PINK	**111**	7

Table S4. Lipid kinase selectivity screening for compound **15** at 10 μM against a kinase panel of 15 protein kinase targets using ADP-Glo assay at the MRC PPU International Centre for Kinase Profiling, University of Dundee.

**Kinase**	**% activity remaining**	**s.d.**
PI3K a	**99**	12
PI3K b	**110**	9
PI3K g	**91**	6
PIK4CB	**71**	23
PIP5K2a	**90**	6
DGK b	**100**	1
SPHK2	**104**	8
PI4K2a	**107**	4
PI3KaE545K	**91**	1
CHKb	**102**	2
SPHK1	**105**	1
DGK z	**106**	7
DGK g	**112**	3
CHKa	**113**	6
PI3KaE524K	**82**	2

Table S5. Lipid kinase selectivity screening for compound **15** at 10 μM against a kinase panel of 13 protein kinase targets generated using KINOMEscan™ technology[a] at DiscoverX.

**Kinase**	**% activity remaining**
PIK3C2B	**68**
PIK3C2G	**28**
PIK3CA	**100**
PIK3CB	**86**
PIK3CD	**80**
PIK3CG	**67**
PIK4CB	**70**
PIKFYVE	**100**
PIP5K1A	**92**
PIP5K1C	**24**
PIP5K2B	**100**
PIP5K2C	**1.9**
VPS34	**64**
PIK3C2B	**68**

Table S6. Lipid kinase K_D data for compound **15** generated using KINOMEscan™ technology[a] at DiscoverX.

**Kinase**	**K_D (nM)**
PIK3C2G	**>3000**
PIP5K1C	**230**
PIP5K2C	**7.1**

[a] Data were generated at Eurofins Discovery using DiscoverX KINOMEscan™ technology. Streptavidin-coated magnetic beads were treated with biotinylated small molecule ligands for 30 minutes at room temperature to generate affinity resins for kinase assays. The liganded beads were blocked with excess biotin and washed with blocking buffer (SeaBlock (Pierce), 1% BSA, 0.05% Tween 20, 1 mM DTT) to remove unbound ligand and to reduce non-specific binding. Binding reactions were assembled by combining kinases, liganded affinity beads, and test compounds in 1x binding buffer (20% SeaBlock, 0.17x PBS, 0.05% Tween 20, 6 mM DTT). Test compounds were prepared as 111X stocks in 100% DMSO. K_Ds were determined using an 11-point 3-fold

compound dilution series with three DMSO control points. All compounds for K_D measurements are distributed by acoustic transfer (non-contact dispensing) in 100% DMSO. The compounds were then diluted directly into the assays such that the final concentration of DMSO was 0.9%. All reactions performed in polypropylene 384-well plate. Each was a final volume of 0.02 ml. The assay plates were incubated at room temperature with shaking for 1 hour and the affinity beads were washed with wash buffer (1x PBS, 0.05% Tween 20). The beads were then re-suspended in elution buffer (1x PBS, 0.05% Tween 20, 0.5 μM nonbiotinylated affinity ligand) and incubated at room temperature with shaking for 30 minutes. The kinase concentration in the eluates was measured by qPCR.

Table S7. Reaction Biology PI3KC2a IC_{50} data for compound **15** using ADP-Glo assay.

**Kinase**	**IC_{50} (nM)**
PI3KC2a	**>3000**

Table S8. Eurofins Discovery Services SafetyScreen™ Functional Panel for compound **15** (10μM): cellular functional assays.

**Assay**	**% of Control Agonist Response**	
	**Agonist Effect**	**Antagonist Effect**
A2A	**-0.9**	**67.3**
alpha 1A (h)	**3.3**	**77**
alpha 2A (h)	**0.3**	**113.2**
beta 1 (h)	**-2.5**	**89.4**
beta 2 (h)	**-2.8**	**90.5**
CB1 (h)	**-36.5**	**89.2**
CB2 (h)	**-43**	**89.7**
CCK1 (CCKA) (h)	**-0.7**	**104.3**
D1 (h)	**-2.3**	**80.7**
D2S (h)	**-3**	**108.8**
ETA (h)	**-1.3**	**106.7**
H1 (h)	**-3.8**	**86.8**
H2 (h)	**-0.8**	**106.4**
kappa (KOP) (h)	**-1.6**	**86.1**
M1 (h)	**0.5**	**77.7**
M2 (h)	**2.2**	**82.7**

M3 (h)	**3.6**	**68.1**
delta (DOP) (h)	**-0.4**	**102.4**
mu (MOP) (h)	**32.5**	**88.7**
5-HT1A (h)	**-2.1**	**105.4**
5HT1B (h)	**19.2**	**99.6**
5-HT2A (h)	**-2**	**103.6**
5-HT2B (h)	**-6.5**	**92.1**
V1a (h)	**15.4**	**81.4**

Table S9. Eurofins Discovery Services SafetyScreen™ Functional Panel for compound **15** (10μM): enzyme and cell-based assays.

**Assay**	**% of Control Values**
COX1(h)	**88.4**
COX2(h)	**82.6**
PDE3A (h)	**95.9**
PDE4D2 (h)	**90.2**
Lck kinase (h)	**107.7**
acetylcholinesterase (h)	**95.2**
MAO-A (h)	**64.5**
norepinephrine uptake	**81.3**
dopamine uptake	**40.9**
5-HT uptake	**98.4**

Table S10. Eurofins Discovery Services SafetyScreen™ Functional Panel for compound **15** (10μM): Ion channel targets.

**Assay**	**Normalised % Inhibition[a]**	**Normalised peak current[b]**
Nav 1.5	**0.4**	**-**
Cav 1.2	**-4.2**	**-**
KCNQ1/minK	**-15.9**	**-**
hERG	**12.2**	**-**
GABA A α1β2g2 (+EC_{20} GABA)	**-**	**95.6**
hnAChR α4β2	**-**	**7.0**

[a]IonWorks Quattro and [b]IonFlux HT electrophysiological platforms.

ADMET experimental methods

MDR1-MDCK Permeability (bi-directional): was performed by Cyprotex Discovery. Briefly, compounds were administered at 10 µM (1% DMSO final) to the apical or basolateral side of a confluent monolayer of MDR1-MDCK cells, then incubated at 37 °C for 60 minutes before appearance on the opposite side of the monolayer was determined LC-MS/MS. The efflux ratio (ER) is calculated from the ratio of B-A to A-B permeabilities.

Turbidimetric aqueous solubility: analysis was performed by Cyprotex Discovery. Compound in DMSO at 10 mM was serially diluted to solutions of 0.1, 0.3, 1 and 3 mM in DMSO, then further diluted 1 in 100 in buffer (0.01 M PBS, pH 7.4, 1% DMSO final). 7 replicate wells of each dilution were equilibrated for 2 hours at 37 °C before absorbance is measured at 620 nm. The solubility is estimated from the concentration of test compound that produces an increase in absorbance above vehicle control (1% DMSO in buffer).

Mouse Microsomal stability: analysis was performed by Cyprotex Discovery. Briefly, test compounds in DMSO were incubated at a concentration of 1 µM (0.25% DMSO final) with mouse hepatic microsomes (0.5 mg protein/mL) in the presence of NADPH (1 mM) at 37 °C. Aliquots were taken at time intervals (0, 5, 15, 30 and 45 min) and stopped by transferring into acetonitrile, then analysed using generic LC-MS/MS conditions for compound remaining, allowing the determination of the half-life for the compound.

Plasma Protein Binding: analysis was performed by Cyprotex Discovery. Briefly, solutions of test compound (0.5% final DMSO concentration) were prepared in 100% mouse plasma. The plasma solution was added to one side of an equilibrium dialysis system while buffer (pH 7.4) was added to the other. Compound concentration on both sides of the membrane was measured by LC-MS/MS and, by comparison to a set of calibration standards (protein-free and protein-containing solutions), the fraction of bound compound was calculated.

Brain Protein Binding: analysis was performed by Shanghai ChemPartner. Briefly, solutions of test compound (1 µM, 0.2% final DMSO concentration) were incubated with 33% CD-1 mouse brain homogenate (w/v) in 50 mM sodium phosphate buffer, pH 7.4). Aliquots of the brain tissue spiked test compounds were applied to the donor side of a dialysis chamber and buffer applied to the other. The dialysis block was incubated for 5 hr at 37 °C, then samples taken for LC-MS/MS analysis.

In vivo pharmacokinetic parameters: were determined in a 4 in 1 cassette study by Shanghai ChemPartner. Briefly, a dosing solution of the 4 test compounds each at 1 mg/mL was prepared using the formulation 10% DMSO, 10% NMP, 40% PEG400, 20% PG, 20% (20% HP-β-CD in water). Male CD1 mice (n=12), aged 6 to 8

weeks, were purchased from Jihui Laboratory Animal Co. LTD, Qualification No.: SCXK (SH) 20170012 20170012023814. Administration was at 5 mL/kg *via* intraperitoneal injection to achieve a dose of 5 mg/kg for each compound. Plasma samples were taken at 8 time points (0.083, 0.25, 0.5, 1, 2, 4, 8 and 24 hr post dose) and brain sampling performed at 3 time points (0.5, 2 and 8 hr post dose). Bioanalysis of compound concentration was measured by LC-MS/MS by comparison to a set of calibration standards.

All animal experiments reported in the manuscript were conducted in compliance with local guidelines and legislation in an AAALAC accredited animal facility.

Unbound brain partition coefficient ($K_{pu,u}$): was determined as a ratio of free brain to free plasma concentrations at 0.5 hr. Free concentrations were calculated using the total concentration in each compartment determined in the *in vivo* PK study, and adjusted using the tissue specific binding level determined *in vitro*.

UPLC methods

Table S11. UPLC method parameters

Method	Column	Additive	Flow rate	Gradient (time, %MeCN in H_2O)
A	BEH C18 (130 Å, 1.7 µm, 2.1 mm × 50 mm)	10 mM NH_3	0.6 mL/min	0 min, 5%; 0.8 min, 5%; 3.3 min, 95%; 4.3 min, 95%; 4.5 min, 5%; 5.5 min, 5%.
B	BEH C18 (130 Å, 1.7 µm, 2.1 mm × 50 mm)	1 mM NH_3	0.6 mL/min	0 min, 5%; 0.8 min, 5%; 3.3 min, 95%; 4.3 min, 95%; 4.5 min, 5%; 5.5 min, 5%.
C	HSS C18 (100 Å, 1.8 µm, 2.1 mm × 50 mm)	0.1% HCO_2H	0.6 mL/min	0 min, 5%; 0.8 min, 5%; 3.3 min, 95%; 4.3 min, 95%; 4.5 min, 5%; 5.5 min, 5%.
D	BEH C18 (130 Å, 1.7 µm, 2.1 mm × 50 mm)	10 mM NH_3	0.6 mL/min	0 min, 5%; 0.8 min, 5%; 8.3 min, 95%; 9.3 min, 95%; 9.5 min, 5%; 10.5 min, 5%.
E	BEH C18 (130 Å, 1.7 µm, 2.1 mm × 50 mm)	1 mM NH_3	0.6 mL/min	0 min, 5%; 0.8 min, 5%; 8.3 min, 95%; 9.3 min, 95%; 9.5 min, 5%; 10.5 min, 5%.
F	HSS C18 (100 Å, 1.8 µm, 2.1 mm × 50 mm)	0.1% HCO_2H	0.6 mL/min	0 min, 5%; 0.8 min, 5%; 8.3 min, 95%; 9.3 min, 95%; 9.5 min, 5%; 10.5 min, 5%.

Synthetic Schemes:

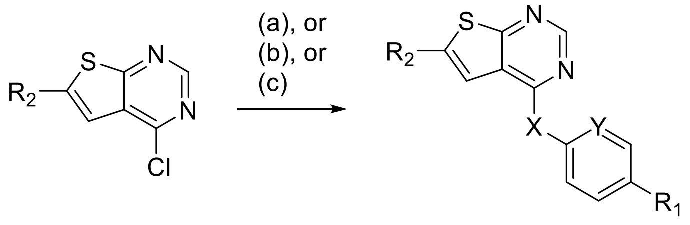

Scheme S1. *Reagents and conditions*: (a) R-NH_2, HCl, dioxane, 100 ^oC. (b) R-NH_2, IPA, 120 ^oC. (c) R-NH_2, Cs_2CO_3, Xantphos, $Pd_2(dba)_3$, 100 to 130 ^oC.

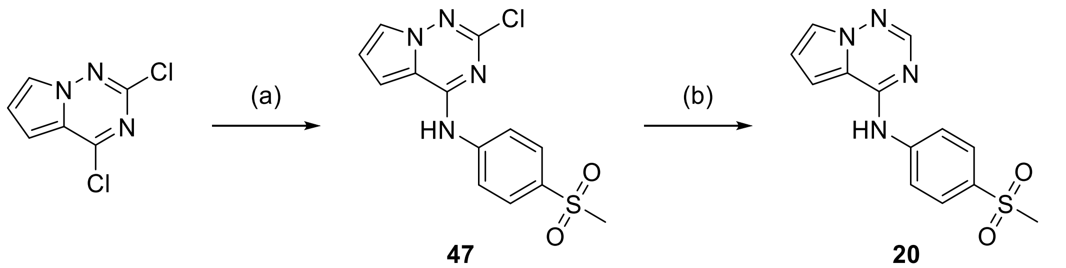

47 **20**

Scheme S2. *Reagents and conditions*: (a) 4-(methylsulfonyl)aniline, IPA, 120 ^oC. (b) H_2, Pd/C, EtOH, rt.

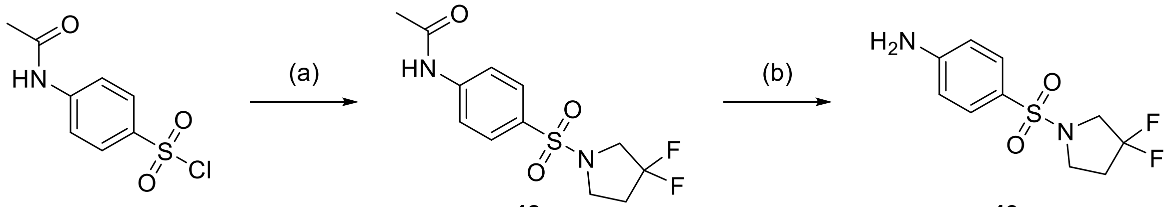

48 **49**

Scheme S3. *Reagents and conditions*: (a) 3,3-Difluoropyrrolidine.HCl, NEt_3, DCM, rt. (b) HCl, MeOH, H_2O, 85 ^oC.

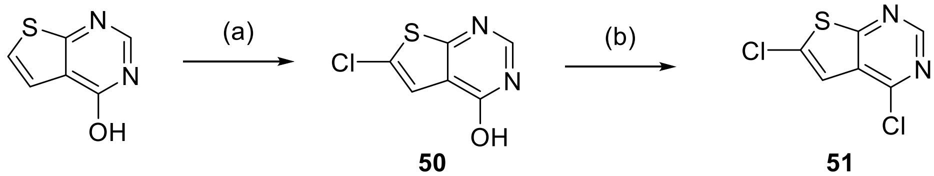

50 **51**

Scheme S4. *Reagents and conditions*: (a) NCS, CH_3CO_2H, 90 ^oC. (b) $POCl_3$, toluene, 100 ^oC.

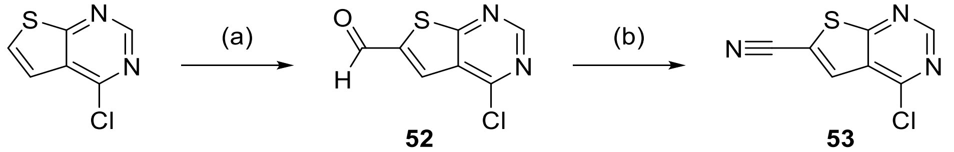

Scheme S5. *Reagents and conditions*: (a) DMF, NH^{i}Pr$_2$, ^{n}BuLi, THF, -78 °C. (b) Ethyl *N*-(mesitylsulfonyl)oxyacetimidate, TfOH, DCM, rt.

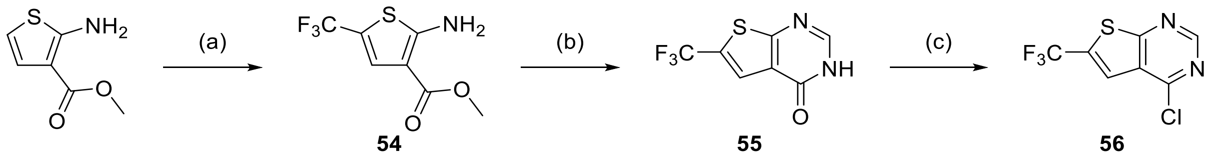

Scheme S6. *Reagents and conditions*: (a) Ru(BPY)$_3$, MeCN, TMEDA, CF_3I, LEDs. (b) HN=CHNH$_2$, IPA, 120 °C. (c) $POCl_3$, 100 °C.

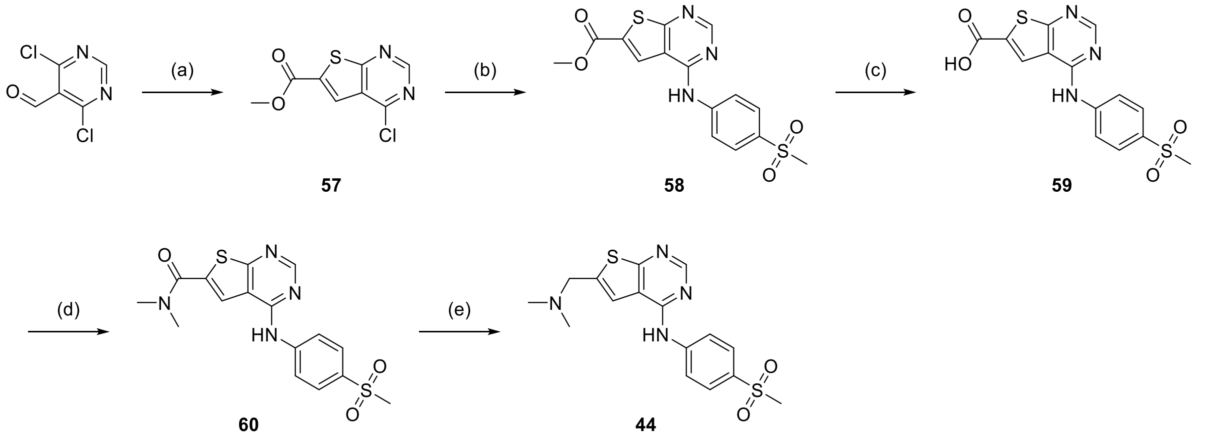

Scheme S7. *Reagents and conditions*: (a) $HSCH_2CO_2CH_3$, DIPEA, DCM, -80 ^oC to rt. (b) 4-(methylsulfonyl)aniline, dioxane, 120 ^oC. (c) LiOH, MeOH, THF, H_2O, rt. (d) HNMe$_2$, HATU, DIPEA, DMF, rt. (e) $LiAlH_4$, THF, 0 ^oC.

References for Supporting Information

(1) Bai, N., Roder, H., Dickson, A., Karanicolas, J., Isothermal Analysis of ThermoFluor Data can readily provide Quantitative Binding Affinities. *Sci Rep*, **2019**, *9*, 2650.

(2) Boffey, H. K., Rooney, T. P. C., Willems, H. M. G., Edwards, S., Green, C., Howard, T., Ogg, D., Romero, T., Scott, D. E., Winpenny, D., Duce, J., Skidmore, J., Clarke, J. H., Andrews, S. P. Development of Selective Phosphatidylinositol 5-Phosphate 4-Kinase γ inhibitors with a Non-ATP-Competitive, Allosteric Binding Mode. *J Med Chem* **2022**, *65* (4), 3359–3370.

(3) Showalter, H. D. H., Bridges, A. J., Zhou, H., Sercel, A. D., McMichael, A., Fry, D. W. Tyrosine Kinase Inhibitors. 16. 6,5,6-Tricyclic Benzothieno[3,2-*d*]pyrimidines and Pyrimido[5,4-*b*]- and -[4,5-*b*]indoles as Potent Inhibitors of the Epidermal Growth Factor Receptor Tyrosine Kinase *J. Med Chem*, **1999**, 42 (26), 5464-5474.

(4) Ravez, S., Arsenlis, S., Barczyk, A., Dupont, A., Frédérick, R., Hesse, S., Kirsch, G., Depreux, P., & Goossens, L. Synthesis and biological evaluation of di-aryl urea derivatives as c-Kit inhibitors. *Bioorg. Med. Chem*., **2015**, 23(22), 7340–7347.

NMR Spectra of compounds from Table 6

Compound **1**: (Insufficient material for ^{13}C spectrum)

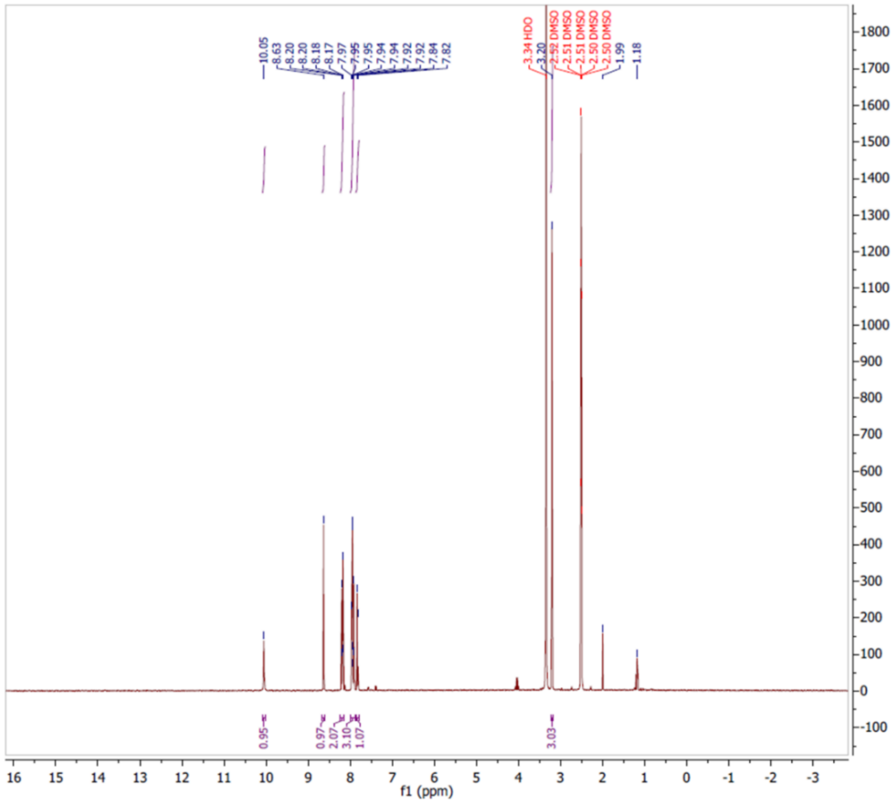

Parameter	Value
Title	HKB3-072-3.10.fid
Origin	Bruker BioSpin GmbH
Instrument	spect
Solvent	DMSO
Temperature	296.4
Pulse Sequence	zg30
Experiment	1D
Probe	5 mm PABBO BB-1H/ D Z-GRD Z104275/ 0242
Number of Scans	16
Receiver Gain	191.9
Relaxation Delay	1.0000
Pulse Width	14.0000
Spectrometer Frequency	300.13
Spectral Width	6009.6
Lowest Frequency	-1151.4
Nucleus	1H
Acquired Size	32768
Spectral Size	65536

^{1}H NMR (300 MHz, DMSO-d_6) δ 10.05 (s, 1H), 8.63 (s, 1H), 8.23 – 8.14 (m, 2H), 8.00 – 7.89 (m, 3H), 7.83 (d, J = 6.0 Hz, 1H), 3.20 (s, 3H).

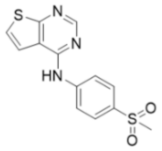

Compound **7**: (Insufficient material for ^{13}C spectrum)

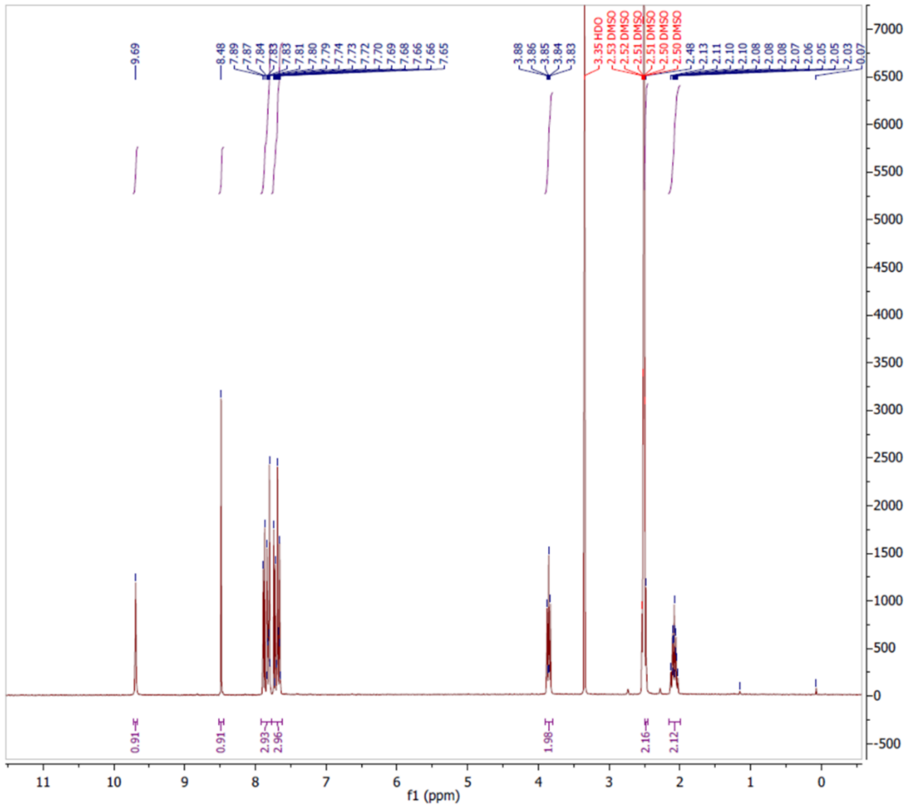

Parameter	Value
Title	tpcr10-099-3.10.fid
Origin	Bruker BioSpin GmbH
Instrument	spect
Solvent	DMSO
Temperature	293.1
Pulse Sequence	zg30
Experiment	1D
Probe	5 mm PABBO BB-1H/ D Z-GRD Z104275/ 0242
Number of Scans	128
Receiver Gain	191.9
Relaxation Delay	1.0000
Pulse Width	14.0000
Acquisition Time	5.4526
Acquisition Date	2022-03-30T15:24:00
Spectrometer Frequency	300.13
Spectral Width	6009.6
Lowest Frequency	-1151.4
Nucleus	1H
Acquired Size	32768
Spectral Size	65536

^{1}H NMR (300 MHz, DMSO-d_6) δ 9.69 (s, 1H), 8.48 (s, 1H), 7.92 – 7.77 (m, 3H), 7.77 – 7.62 (m, 3H), 3.85 (t, J = 7.0 Hz, 2H), 2.50 – 2.46 (m, 2H), 2.08 (tt, J = 7.7, 6.8 Hz, 2H).

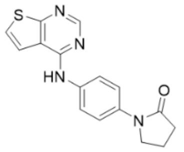

Compound **15**

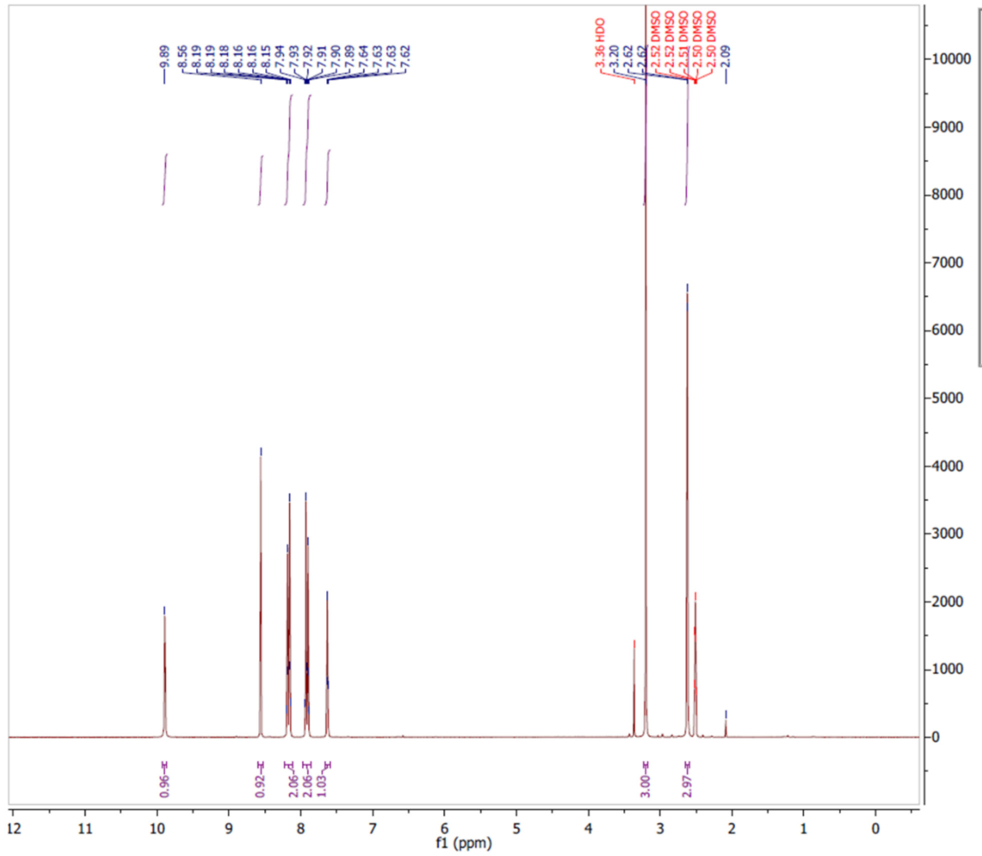

Parameter	Value
Title	tpcr11-47-4.10.fid
Origin	Bruker BioSpin GmbH
Instrument	spect
Solvent	DMSO
Temperature	292.1
Pulse Sequence	zg30
Experiment	1D
Probe	5 mm PABBO BB-1H/ D Z-GRD Z104275/ 0242
Number of Scans	32
Receiver Gain	191.9
Relaxation Delay	1.0000
Pulse Width	15.0000
Spectrometer Frequency	300.13
Spectral Width	6009.6
Lowest Frequency	-1151.4
Nucleus	1H
Acquired Size	32768
Spectral Size	65536

^{1}H NMR (300 MHz, DMSO-d_6) δ 9.89 (s, 1H), 8.56 (s, 1H), 8.22 – 8.12 (m, 2H), 7.97 – 7.86 (m, 2H), 7.63 (q, J = 1.2 Hz, 1H), 3.20 (s, 3H), 2.62 (d, J = 1.2 Hz, 3H).

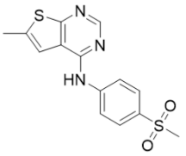

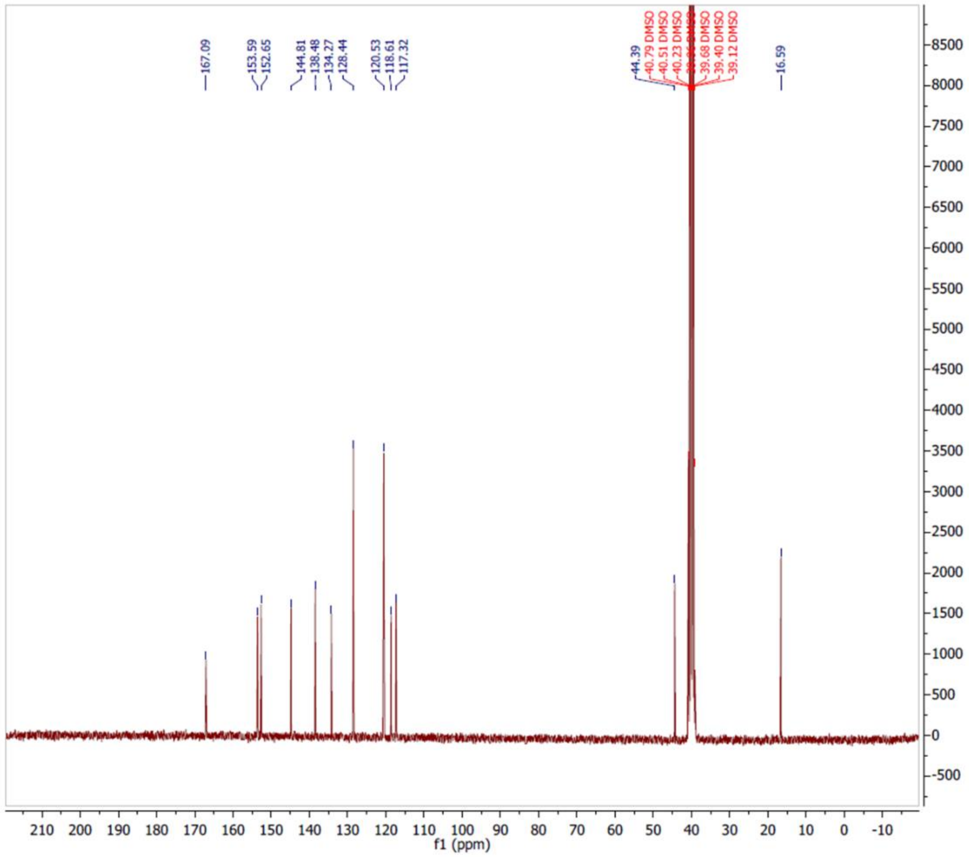

Parameter	Value
Title	tpcr11-47-4.12.fid
Origin	Bruker BioSpin GmbH
Instrument	spect
Solvent	DMSO
Temperature	292.7
Pulse Sequence	zgpg30
Experiment	1D
Probe	5 mm PABBO BB-1H/ D Z-GRD Z104275/ 0242
Number of Scans	4096
Receiver Gain	191.9
Relaxation Delay	2.0000
Pulse Width	10.0000
Acquisition Time	1.8175
Acquisition Date	2022-10-13T05:10:00
Spectrometer Frequency	75.48
Spectral Width	18028.8
Lowest Frequency	-1468.5
Nucleus	13C
Acquired Size	32768
Spectral Size	65536

^{13}C NMR (75 MHz, DMSO) δ 167.09, 153.59, 152.65, 144.81, 138.48, 134.27, 128.44, 120.53, 118.61, 117.32, 44.39, 16.59.

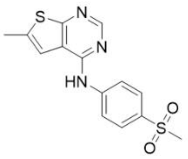

Compound **23**

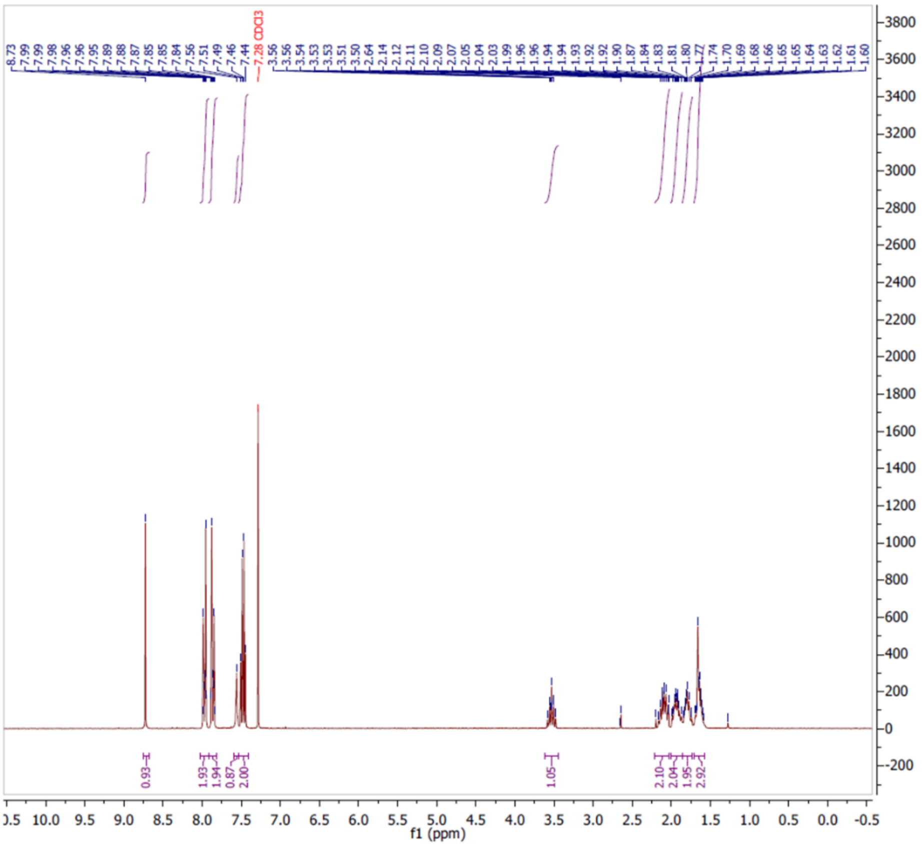

Parameter	Value
Title	SDE3-057-2 CDCl3.10.fid
Origin	Bruker BioSpin GmbH
Instrument	spect
Solvent	CDCl3
Temperature	297.8
Pulse Sequence	zg30
Experiment	1D
Probe	5 mm PABBO BB-1H/ D Z-GRD Z104275/ 0242
Number of Scans	16
Receiver Gain	191.9
Relaxation Delay	1.0000
Pulse Width	14.0000
Spectrometer Frequency	300.13
Spectral Width	6009.6
Lowest Frequency	-1151.4
Nucleus	1H
Acquired Size	32768
Spectral Size	65536

^{1}H NMR (300 MHz, Chloroform-*d*) δ 8.73 (s, 1H), 8.02 – 7.92 (m, 2H), 7.92 – 7.81 (m, 2H), 7.56 (s, 1H), 7.53 – 7.41 (m, 2H), 3.53 (tt, *J* = 8.7, 7.2 Hz, 1H), 2.21 – 2.02 (m, 2H), 2.01 – 1.86 (m, 2H), 1.86 – 1.73 (m, 2H), 1.71 – 1.58 (m, 2H).

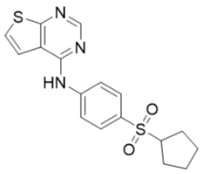

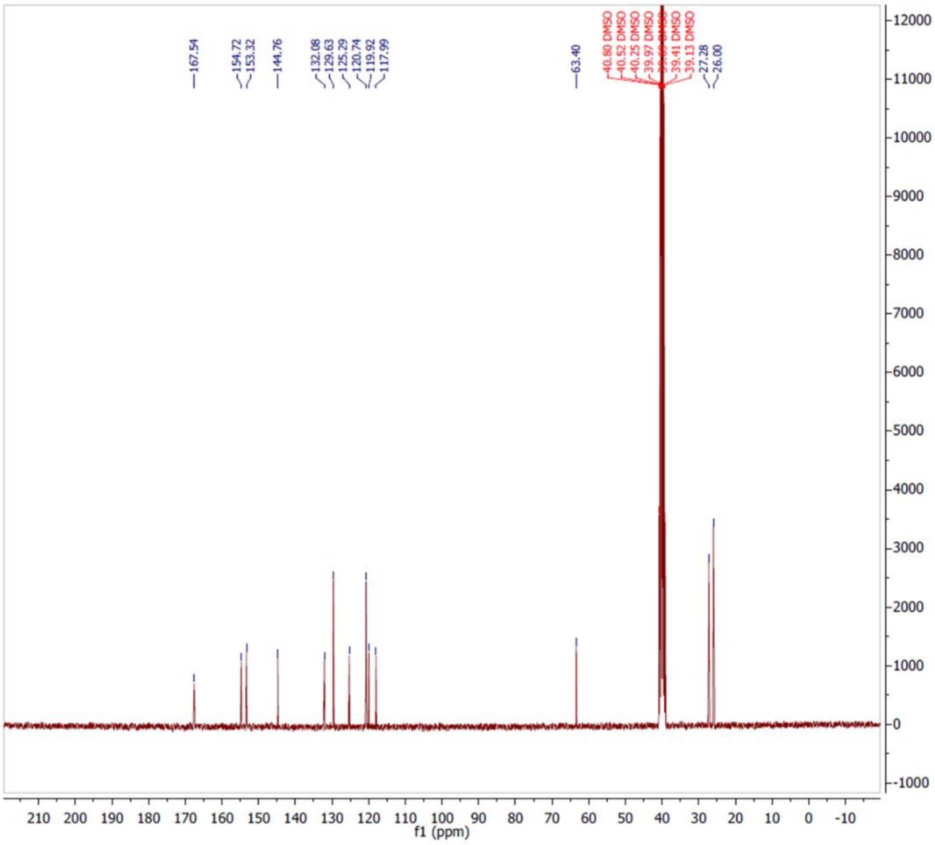

Parameter	Value
Title	tpcr11-46-1.11.fid
Origin	Bruker BioSpin GmbH
Instrument	spect
Solvent	DMSO
Temperature	294.0
Pulse Sequence	zgpg30
Experiment	1D
Probe	5 mm PABBO BB-1H/ D Z-GRD Z104275/ 0242
Number of Scans	4096
Receiver Gain	191.9
Relaxation Delay	2.0000
Pulse Width	10.0000
Spectrometer Frequency	75.48
Spectral Width	18028.8
Lowest Frequency	-1468.5
Nucleus	13C
Acquired Size	32768
Spectral Size	65536

^{13}C NMR (75 MHz, DMSO) δ 167.54, 154.72, 153.32, 144.76, 132.08, 129.63, 125.29, 120.74, 119.92, 117.99, 63.40, 27.28, 26.00.

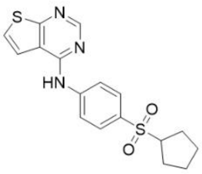

Compound **24**

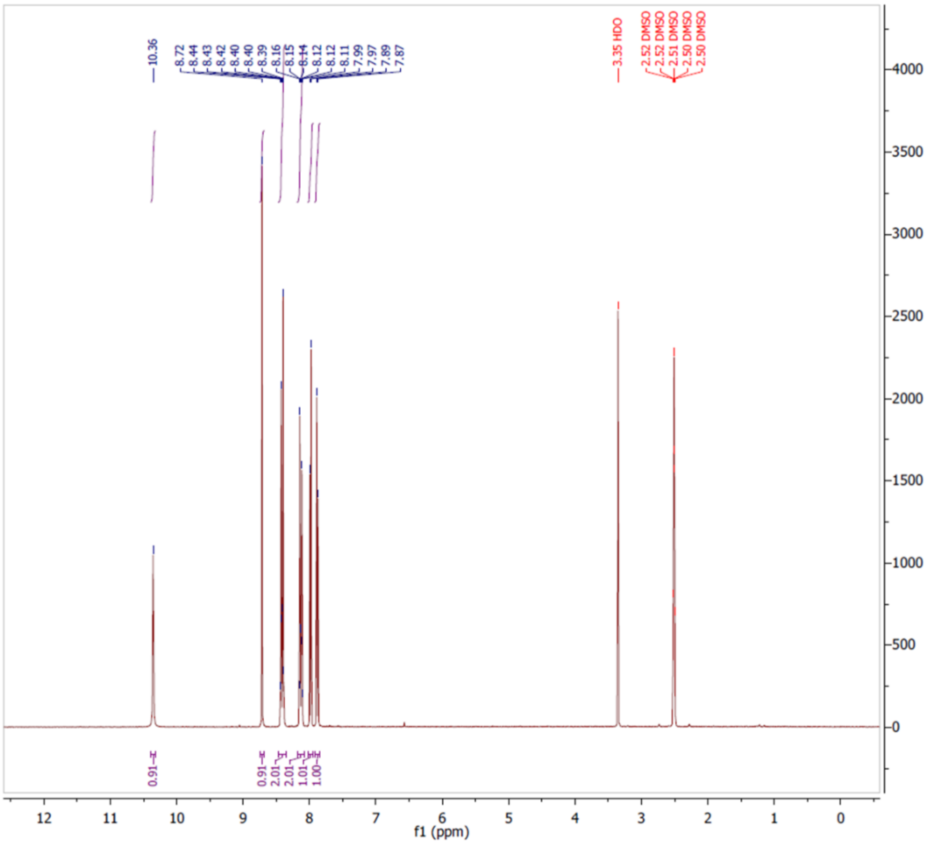

Parameter	Value
Title	tpcr11-46-2.10.fid
Origin	Bruker BioSpin GmbH
Instrument	spect
Solvent	DMSO
Temperature	293.3
Pulse Sequence	zg30
Experiment	1D
Probe	5 mm PABBO BB-1H/ D Z-GRD Z104275/ 0242
Number of Scans	32
Receiver Gain	191.9
Relaxation Delay	1.0000
Pulse Width	15.0000
Spectrometer Frequency	300.13
Spectral Width	6009.6
Lowest Frequency	-1151.4
Nucleus	1H
Acquired Size	32768
Spectral Size	65536

^{1}H NMR (300 MHz, DMSO-d_6) δ 10.36 (s, 1H), 8.72 (s, 1H), 8.47 – 8.36 (m, 2H), 8.19 – 8.08 (m, 2H), 7.98 (d, J = 6.1 Hz, 1H), 7.88 (d, J = 6.0 Hz, 1H).

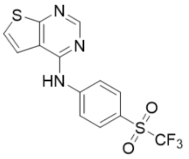

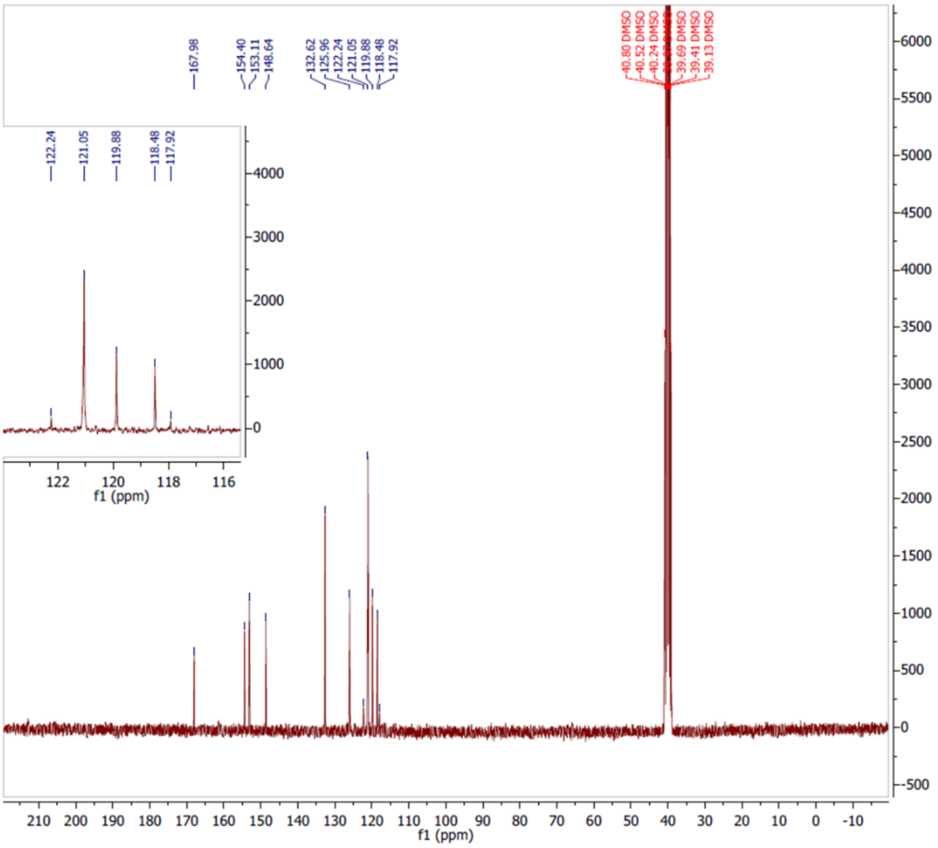

Parameter	Value
Title	tpcr11-46-2.12.fid
Origin	Bruker BioSpin GmbH
Instrument	spect
Solvent	DMSO
Temperature	294.5
Pulse Sequence	zgpg30
Experiment	1D
Probe	5 mm PABBO BB-1H/ D Z-GRD Z104275/ 0242
Number of Scans	4096
Receiver Gain	191.9
Relaxation Delay	2.0000
Pulse Width	10.0000
Acquisition Time	1.8175
Acquisition Date	2022-09-24T23:43:00
Spectrometer Frequency	75.48
Spectral Width	18028.8
Lowest Frequency	-1468.5
Nucleus	13C
Acquired Size	32768
Spectral Size	65536

^{13}C NMR (75 MHz, DMSO) δ 167.98, 154.40, 153.11, 148.64, 132.62, 125.96, 122.24, 121.05, 119.88, 118.48, 117.92.

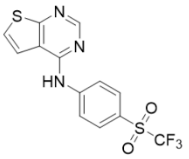

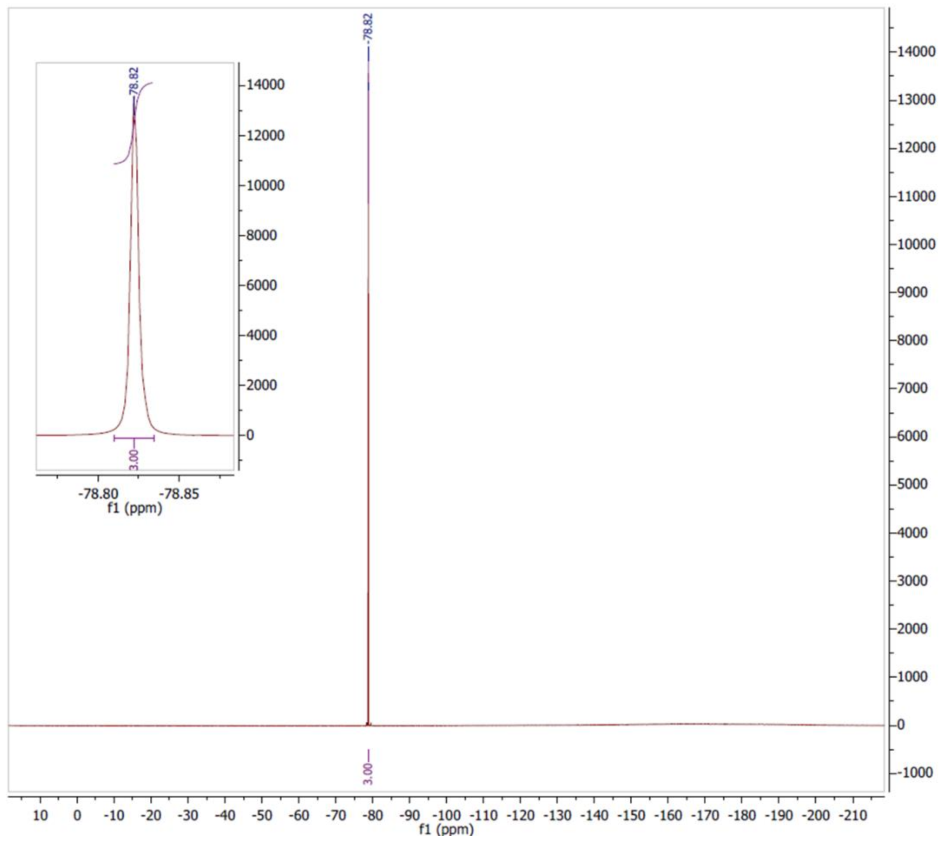

Parameter	Value
Title	tpcr11-46-2.11.fid
Origin	Bruker BioSpin GmbH
Instrument	spect
Solvent	DMSO
Temperature	293.2
Pulse Sequence	zgflqn
Experiment	1D
Probe	5 mm PABBO BB-1H/ D Z-GRD Z104275/ 0242
Number of Scans	32
Receiver Gain	191.9
Relaxation Delay	1.0000
Pulse Width	15.0000
Acquisition Time	0.9787
Acquisition Date	2022-09-24T19:16:00
Spectrometer Frequency	282.38
Spectral Width	66964.3
Lowest Frequency	-61722.4
Nucleus	19F
Acquired Size	65536
Spectral Size	131072

^{19}F NMR (282 MHz, DMSO-d_6) δ -78.82.

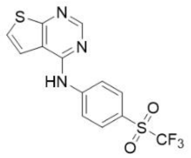

Compound **35**

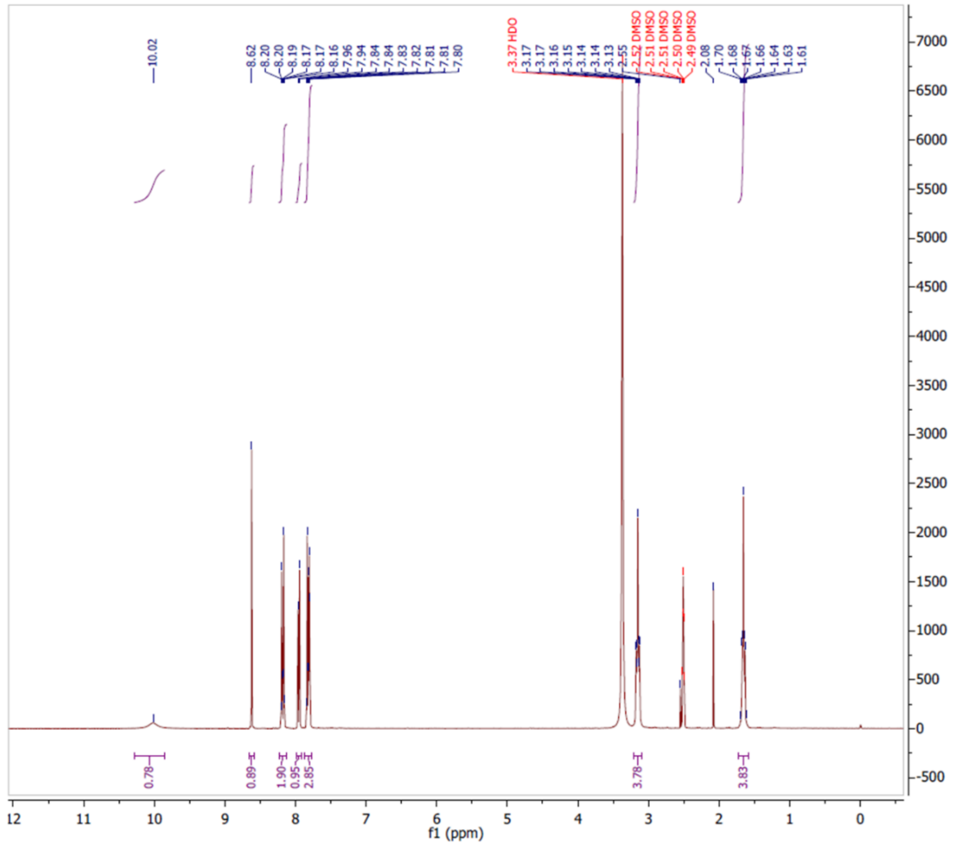

Parameter	Value
Title	HKB4-048-2-3.10.fid
Origin	Bruker BioSpin GmbH
Instrument	spect
Solvent	DMSO
Temperature	295.4
Pulse Sequence	zg30
Experiment	1D
Probe	5 mm PABBO BB-1H/ D Z-GRD Z104275/ 0242
Number of Scans	16
Receiver Gain	191.9
Relaxation Delay	1.0000
Pulse Width	14.0000
Spectrometer Frequency	300.13
Spectral Width	6009.6
Lowest Frequency	-1151.4
Nucleus	1H
Acquired Size	32768
Spectral Size	65536

^{1}H NMR (300 MHz, DMSO-d_6) δ 10.02 (s, 1H), 8.62 (s, 1H), 8.23 – 8.13 (m, 2H), 7.95 (d, J = 6.0 Hz, 1H), 7.87 – 7.77 (m, 3H), 3.20 – 3.10 (m, 4H), 1.73 – 1.58 (m, 4H).

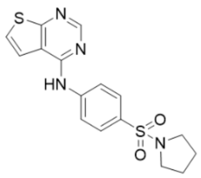

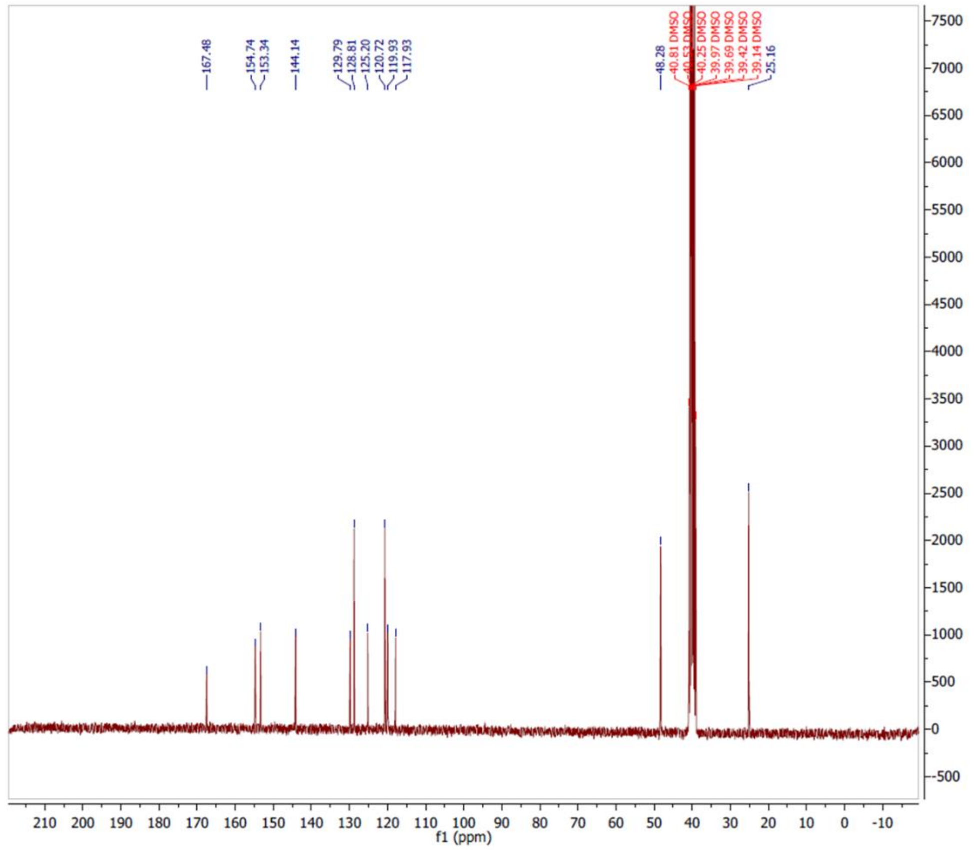

Parameter	Value
Title	tpcr11-46-3.11.fid
Origin	Bruker BioSpin GmbH
Instrument	spect
Solvent	DMSO
Temperature	294.8
Pulse Sequence	zgpg30
Experiment	1D
Probe	5 mm PABBO BB-1H/ D Z-GRD Z104275/ 0242
Number of Scans	4096
Receiver Gain	191.9
Relaxation Delay	2.0000
Pulse Width	10.0000
Acquisition Time	1.8175
Acquisition Date	2022-09-25T05:39:00
Spectrometer Frequency	75.48
Spectral Width	18028.8
Lowest Frequency	-1468.5
Nucleus	13C
Acquired Size	32768
Spectral Size	65536

^{13}C NMR (75 MHz, DMSO) δ 167.48, 154.74, 153.34, 144.14, 129.79, 128.81, 125.20, 120.72, 119.93, 117.93, 48.28, 25.16.

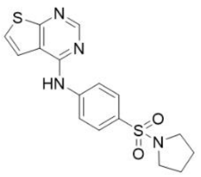

Compound **36**

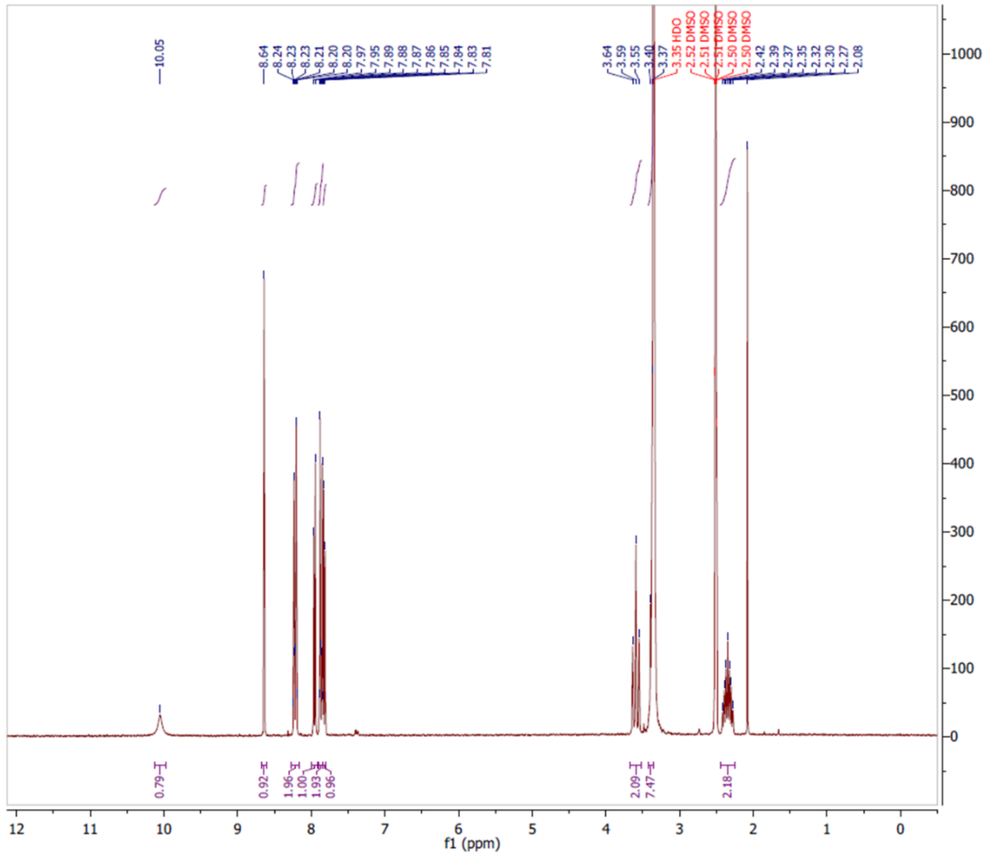

Parameter	Value
Title	HKB5_026_3.10.fid
Origin	Bruker BioSpin GmbH
Instrument	spect
Solvent	DMSO
Temperature	298.3
Pulse Sequence	zg30
Experiment	1D
Probe	5 mm PABBO BB-1H/ D Z-GRD Z104275/ 0242
Number of Scans	16
Receiver Gain	191.9
Relaxation Delay	1.0000
Pulse Width	14.0000
Spectrometer Frequency	300.13
Spectral Width	6009.6
Lowest Frequency	-1151.4
Nucleus	1H
Acquired Size	32768
Spectral Size	65536

^{1}H NMR (300 MHz, DMSO-d_6) δ 10.05 (s, 1H), 8.64 (s, 1H), 8.27 – 8.17 (m, 2H), 7.96 (d, J = 6.0 Hz, 1H), 7.90 – 7.84 (m, 2H), 7.82 (d, J = 6.0 Hz, 1H), 3.59 (t, J = 13.1 Hz, 2H), 3.38 (d, J = 7.3 Hz, 7H), 2.35 (tt, J = 14.3, 7.3 Hz, 2H).

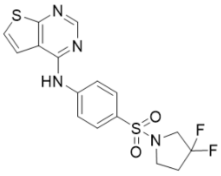

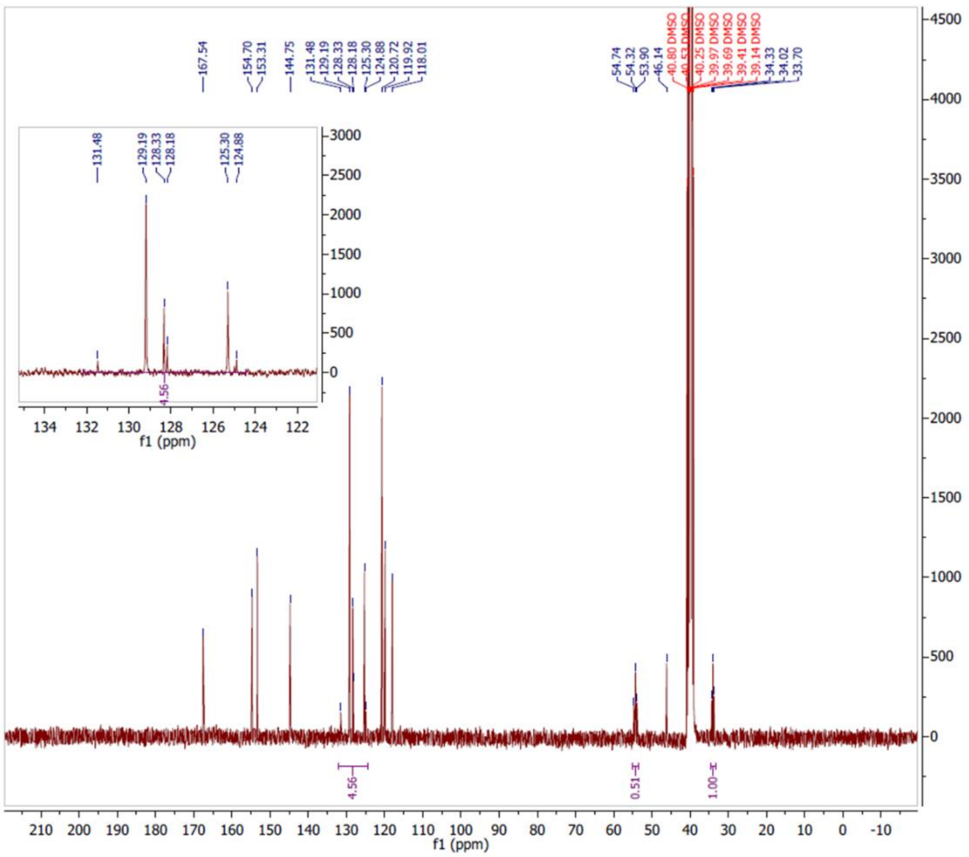

Parameter	Value
Title	tpcr11-46-4.12.fid
Origin	Bruker BioSpin GmbH
Instrument	spect
Solvent	DMSO
Temperature	295.5
Pulse Sequence	zgpg30
Experiment	1D
Probe	5 mm PABBO BB-1H/ D Z-GRD Z104275/ 0242
Number of Scans	4096
Receiver Gain	191.9
Relaxation Delay	2.0000
Pulse Width	10.0000
Acquisition Time	1.8175
Acquisition Date	2022-09-25T11:38:00
Spectrometer Frequency	75.48
Spectral Width	18028.8
Lowest Frequency	-1468.5
Nucleus	13C
Acquired Size	32768
Spectral Size	65536

^{13}C NMR (75 MHz, DMSO) δ 167.54, 154.70, 153.31, 144.75, 131.48, 129.19, 128.33, 128.18, 125.30, 124.88, 120.72, 119.92, 118.01, 54.74, 54.32, 53.90, 46.14, 34.33, 34.02, 33.70.

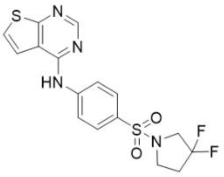

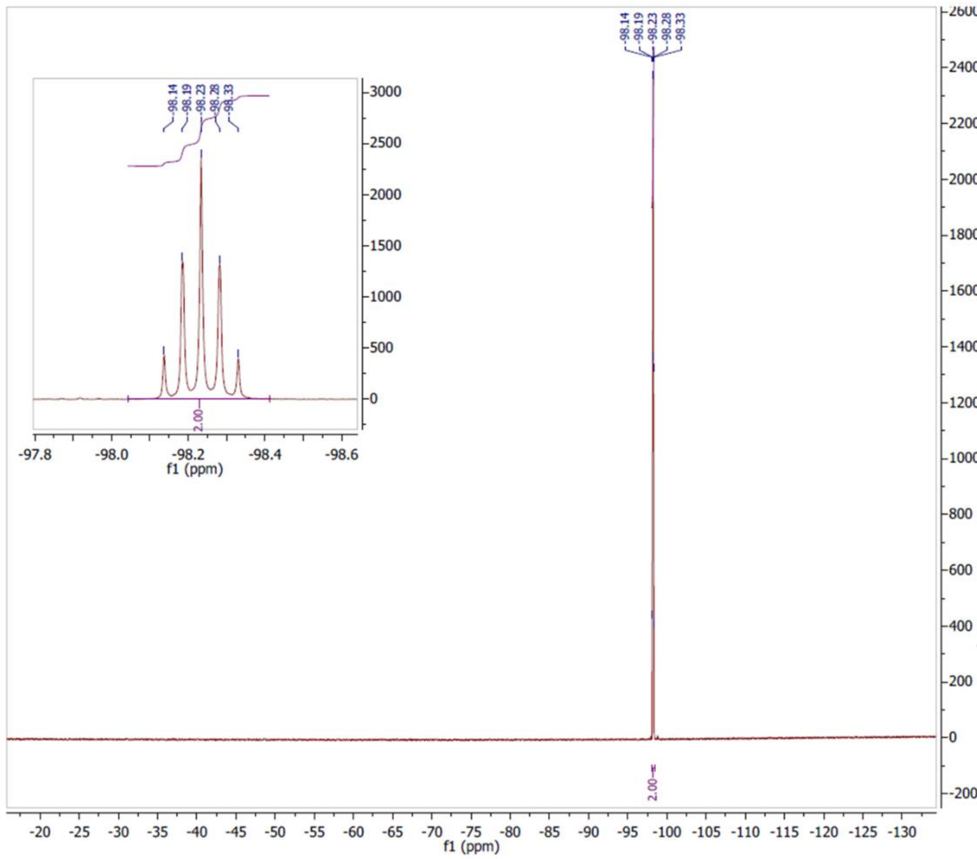

Parameter	Value
Title	tpcr11-46-4.11.fid
Origin	Bruker BioSpin GmbH
Instrument	spect
Solvent	DMSO
Temperature	293.8
Pulse Sequence	zgflqn
Experiment	1D
Probe	5 mm PABBO BB-1H/ D Z-GRD Z104275/ 0242
Number of Scans	32
Receiver Gain	191.9
Relaxation Delay	1.0000
Pulse Width	15.0000
Acquisition Time	0.9787
Acquisition Date	2022-09-25T07:10:00
Spectrometer Frequency	282.38
Spectral Width	66964.3
Lowest Frequency	-61722.4
Nucleus	19F
Acquired Size	65536
Spectral Size	131072

^{19}F NMR (282 MHz, DMSO-d_6) δ -98.23 (p, J = 13.6 Hz).

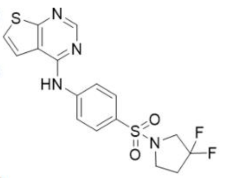

Compound **37**

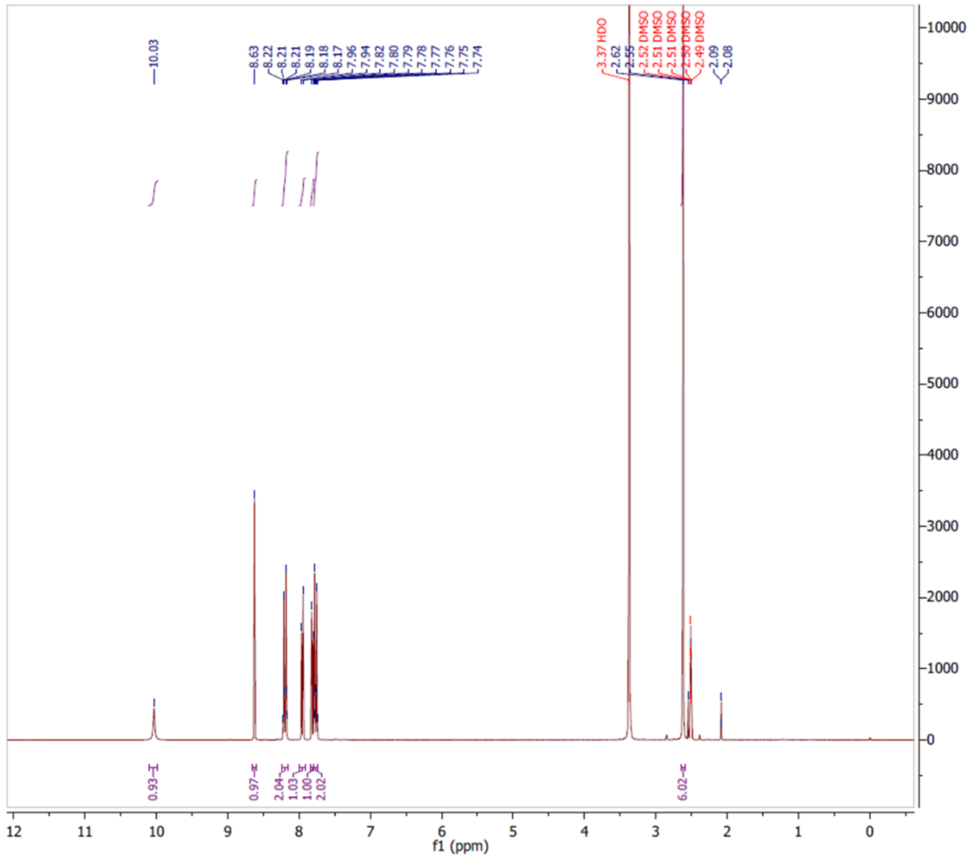

Parameter	Value
Title	HKB4-048-1-3.10.fid
Origin	Bruker BioSpin GmbH
Instrument	spect
Solvent	DMSO
Temperature	295.3
Pulse Sequence	zg30
Experiment	1D
Probe	5 mm PABBO BB-1H/ D Z-GRD Z104275/ 0242
Number of Scans	16
Receiver Gain	191.9
Relaxation Delay	1.0000
Pulse Width	14.0000
Spectrometer Frequency	300.13
Spectral Width	6009.6
Lowest Frequency	-1151.4
Nucleus	1H
Acquired Size	32768
Spectral Size	65536

^{1}H NMR (300 MHz, DMSO-d_6) δ 10.03 (s, 1H), 8.63 (s, 1H), 8.24 – 8.15 (m, 2H), 7.95 (d, J = 6.0 Hz, 1H), 7.81 (d, J = 6.0 Hz, 1H), 7.79 – 7.74 (m, 2H), 2.62 (s, 6H).

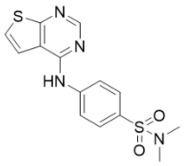

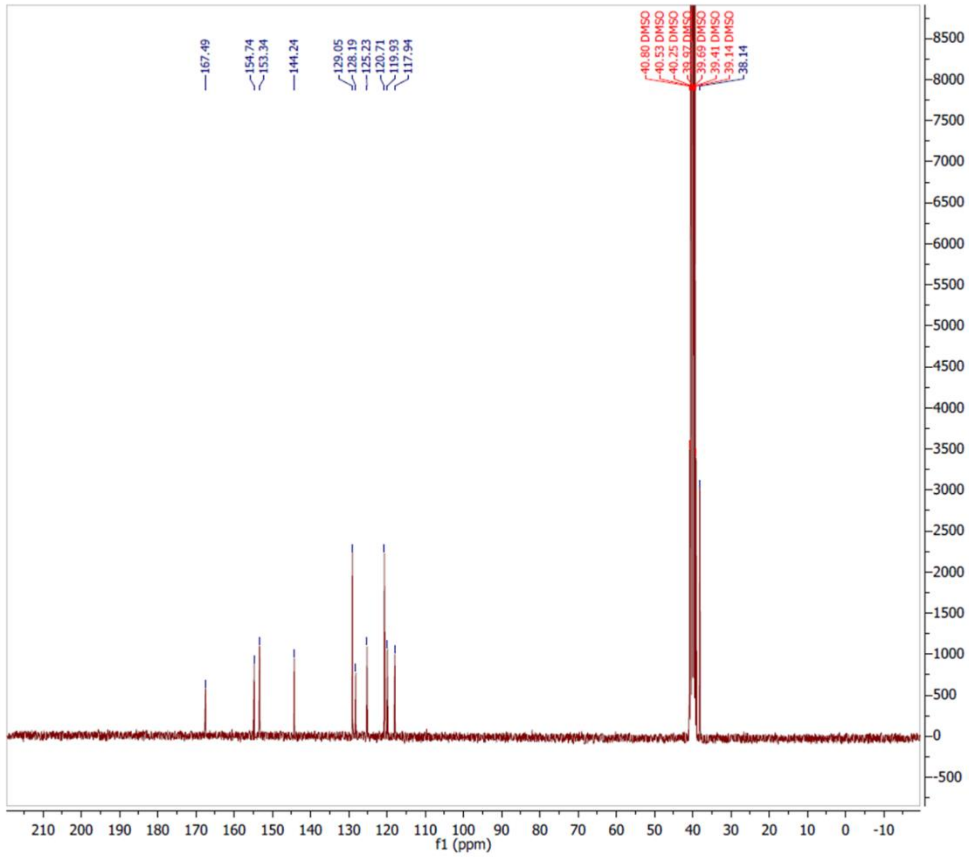

Parameter	Value
Title	tpcr11-46-5.11.fid
Origin	Bruker BioSpin GmbH
Instrument	spect
Solvent	DMSO
Temperature	294.6
Pulse Sequence	zgpg30
Experiment	1D
Probe	5 mm PABBO BB-1H/ D Z-GRD Z104275/ 0242
Number of Scans	4096
Receiver Gain	191.9
Relaxation Delay	2.0000
Pulse Width	10.0000
Acquisition Time	1.8175
Acquisition Date	2022-09-25T17:33:00
Spectrometer Frequency	75.48
Spectral Width	18028.8
Lowest Frequency	-1468.5
Nucleus	13C
Acquired Size	32768
Spectral Size	65536

^{13}C NMR (75 MHz, DMSO) δ 167.49, 154.74, 153.34, 144.24, 129.05, 128.19, 125.23, 120.71, 119.93, 117.94, 38.14.

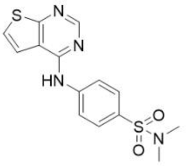

Compound **38**

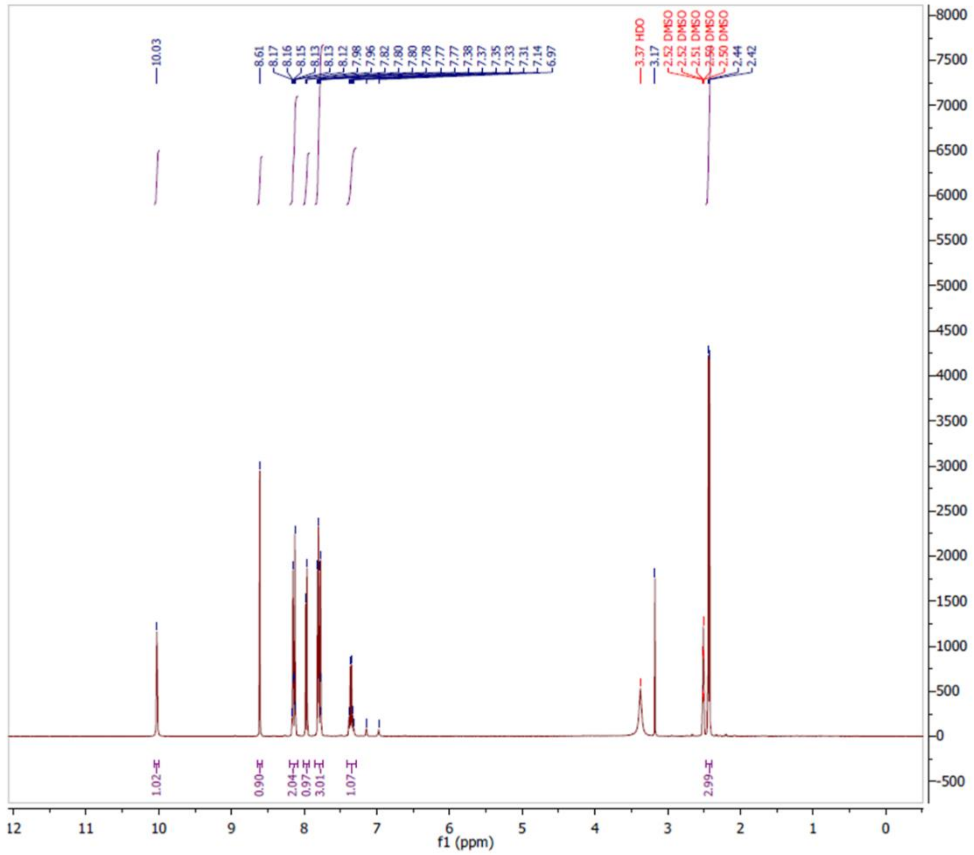

Parameter	Value
Title	tpcr11-46-6-3.10.fid
Origin	Bruker BioSpin GmbH
Instrument	spect
Solvent	DMSO
Temperature	294.5
Pulse Sequence	zg30
Experiment	1D
Probe	5 mm PABBO BB-1H/ D Z-GRD Z104275/ 0242
Number of Scans	16
Receiver Gain	191.9
Relaxation Delay	1.0000
Pulse Width	15.0000
Spectrometer Frequency	300.13
Spectral Width	6009.6
Lowest Frequency	-1151.4
Nucleus	1H
Acquired Size	32768
Spectral Size	65536

^{1}H NMR (300 MHz, DMSO-d_6) δ 10.03 (s, 1H), 8.61 (s, 1H), 8.20 – 8.09 (m, 2H), 7.97 (d, J = 6.1 Hz, 1H), 7.85 – 7.74 (m, 3H), 7.41 – 7.28 (m, 1H), 2.43 (d, J = 5.1 Hz, 3H).

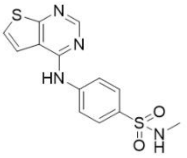

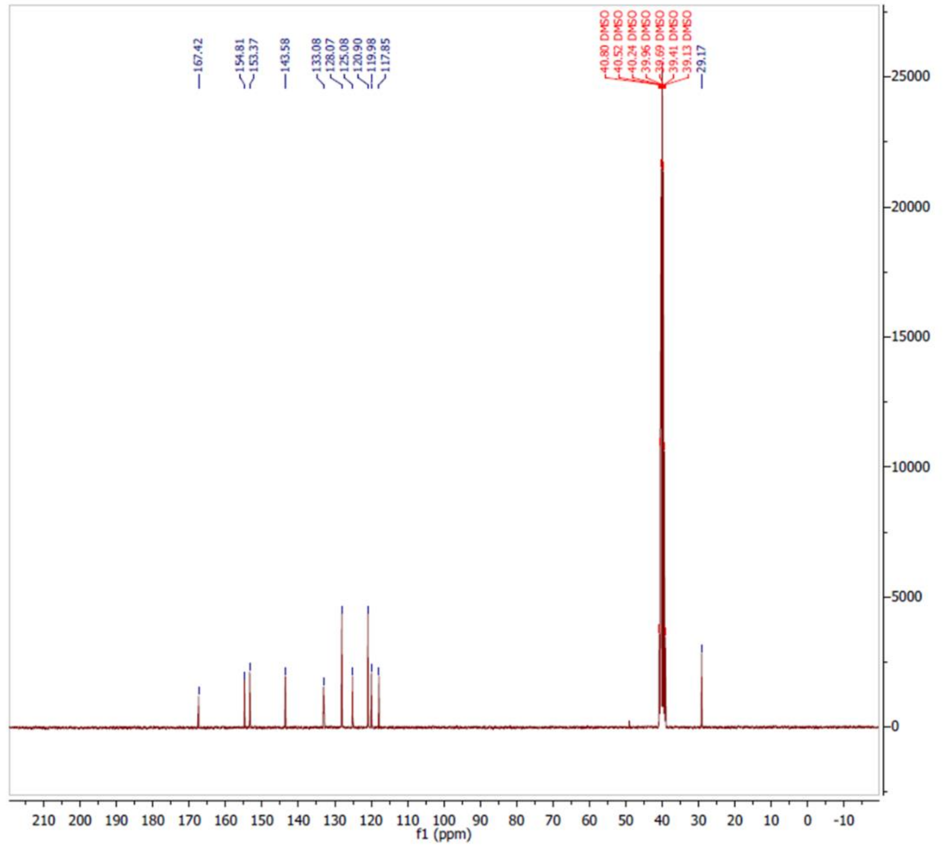

Parameter	Value
Title	tpcr11-46-6-3.11.fid
Origin	Bruker BioSpin GmbH
Instrument	spect
Solvent	DMSO
Temperature	294.1
Pulse Sequence	zgpg30
Experiment	1D
Probe	5 mm PABBO BB-1H/ D Z-GRD Z104275/ 0242
Number of Scans	4096
Receiver Gain	191.9
Relaxation Delay	2.0000
Pulse Width	10.0000
Spectrometer Frequency	75.48
Spectral Width	18028.8
Lowest Frequency	-1468.5
Nucleus	13C
Acquired Size	32768
Spectral Size	65536

^{13}C NMR (75 MHz, DMSO) δ 167.42, 154.81, 153.37, 143.58, 133.08, 128.07, 125.08, 120.90, 119.98, 117.85, 29.17.

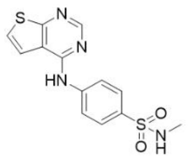

Compound **40**

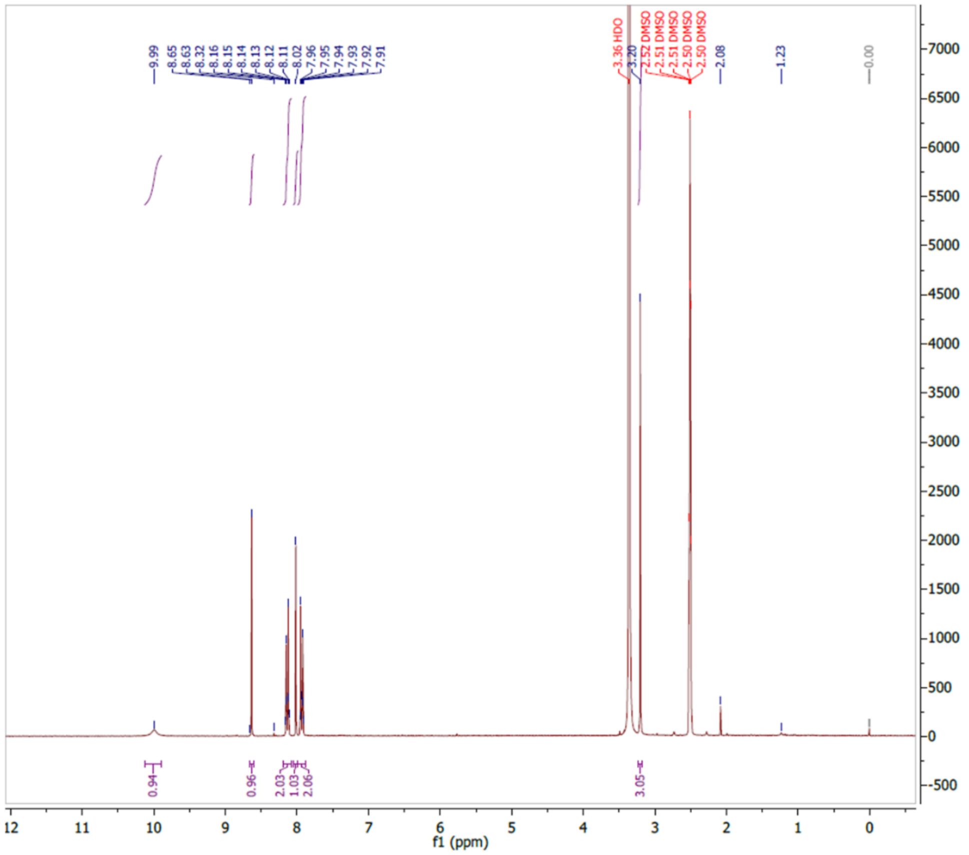

Parameter	Value
Title	hkb4-089-3.10.fid
Origin	Bruker BioSpin GmbH
Instrument	spect
Solvent	DMSO
Temperature	308.1
Pulse Sequence	zg30
Experiment	1D
Probe	5 mm PABBO BB-1H/ D Z-GRD Z104275/ 0242
Number of Scans	64
Receiver Gain	191.9
Relaxation Delay	1.0000
Pulse Width	14.0000
Spectrometer Frequency	300.13
Spectral Width	6009.6
Lowest Frequency	-1151.4
Nucleus	1H
Acquired Size	32768
Spectral Size	65536

^{1}H NMR (300 MHz, DMSO-d_6) δ 9.99 (s, 1H), 8.63 (s, 1H), 8.19 – 8.08 (m, 2H), 8.02 (s, 1H), 7.99 – 7.88 (m, 2H), 3.20 (s, 3H).

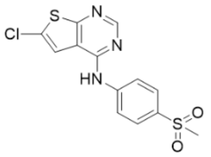

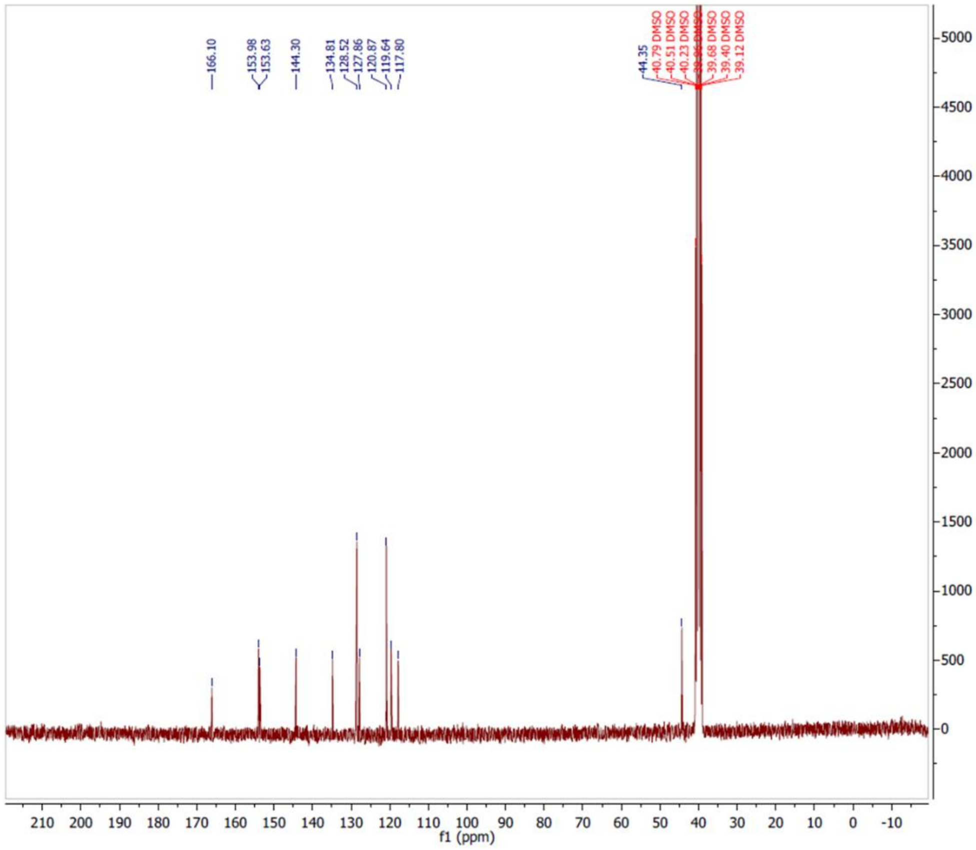

Parameter	Value
Title	tpcr11-47-1.11.fid
Origin	Bruker BioSpin GmbH
Instrument	spect
Solvent	DMSO
Temperature	292.2
Pulse Sequence	zgpg30
Experiment	1D
Probe	5 mm PABBO BB-1H/ D Z-GRD Z104275/ 0242
Number of Scans	4096
Receiver Gain	191.9
Relaxation Delay	2.0000
Pulse Width	10.0000
Acquisition Time	1.8175
Acquisition Date	2022-10-11T22:20:00
Spectrometer Frequency	75.48
Spectral Width	18028.8
Lowest Frequency	-1468.5
Nucleus	13C
Acquired Size	32768
Spectral Size	65536

^{13}C NMR (75 MHz, DMSO) δ 166.10, 153.98, 153.63, 144.30, 134.81, 128.52, 127.86, 120.87, 119.64, 117.80, 44.35.

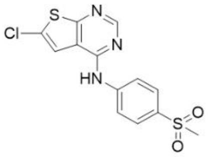

Compound **41:** (Insufficient material for ^{13}C spectrum)

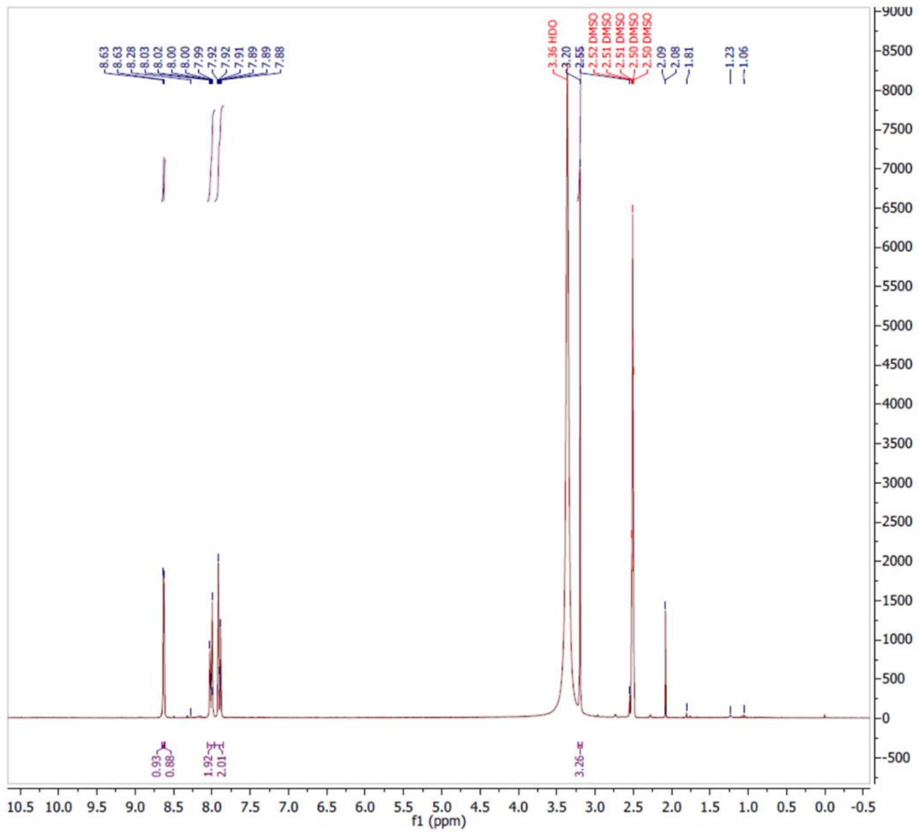

Parameter	Value
Title	HKB4-80-3.10.fid
Origin	Bruker BioSpin GmbH
Instrument	spect
Solvent	DMSO
Temperature	317.5
Pulse Sequence	zg30
Experiment	1D
Probe	5 mm PABBO BB-1H/ D Z-GRD Z104275/ 0242
Number of Scans	64
Receiver Gain	191.9
Relaxation Delay	1.0000
Pulse Width	14.0000
Spectrometer Frequency	300.13
Spectral Width	6009.6
Lowest Frequency	-1151.4
Nucleus	1H
Acquired Size	32768
Spectral Size	65536

^{1}H NMR (300 MHz, DMSO-d_6) δ 8.63 (s, 1H), 8.63 (s, 1H), 8.06 – 7.96 (m, 2H), 7.96 – 7.85 (m, 2H), 3.20 (s, 3H).

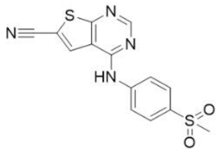

Compound **42**

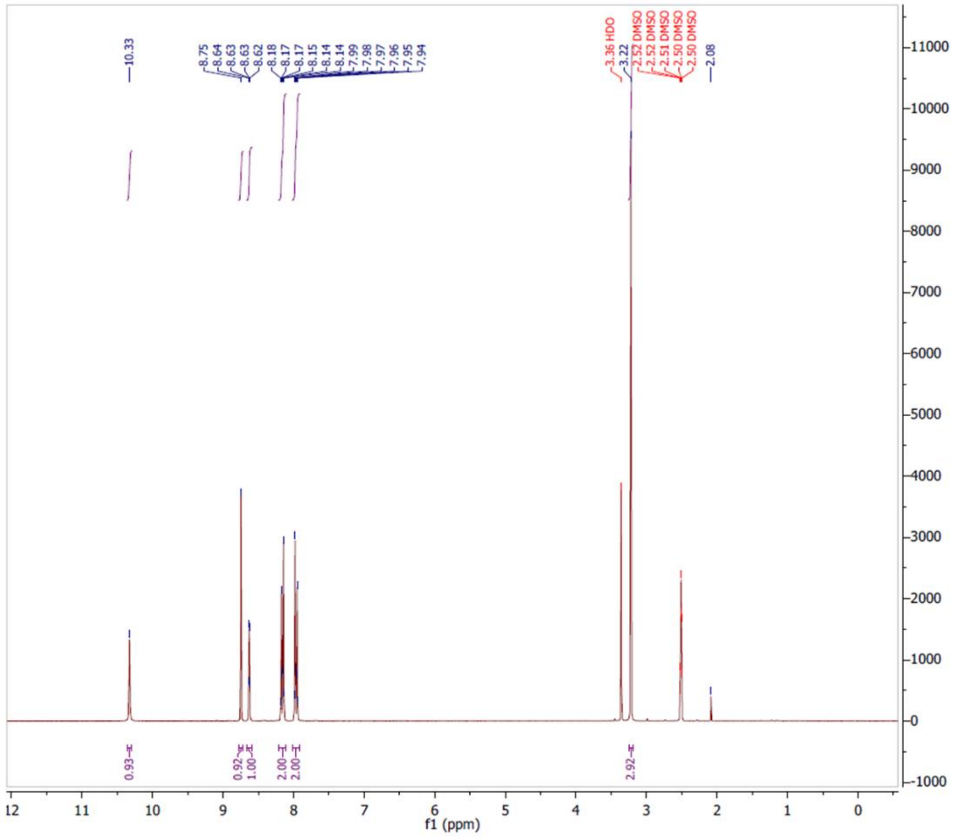

Parameter	Value
Title	tpcr11-47-3.10.fid
Origin	Bruker BioSpin GmbH
Instrument	spect
Solvent	DMSO
Temperature	292.2
Pulse Sequence	zg30
Experiment	1D
Probe	5 mm PABBO BB-1H/ D Z-GRD Z104275/ 0242
Number of Scans	32
Receiver Gain	191.9
Relaxation Delay	1.0000
Pulse Width	15.0000
Acquisition Time	5.4526
Acquisition Date	2022-10-12T18:43:00
Spectrometer Frequency	300.13
Spectral Width	6009.6
Lowest Frequency	-1151.4
Nucleus	1H
Acquired Size	32768
Spectral Size	65536

^{1}H NMR (300 MHz, DMSO-d_6) δ 10.33 (s, 1H), 8.75 (s, 1H), 8.63 (q, J = 1.4 Hz, 1H), 8.21 – 8.11 (m, 2H), 8.02 – 7.91 (m, 2H), 3.22 (s, 3H).

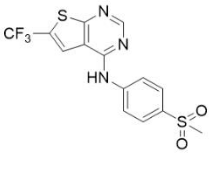

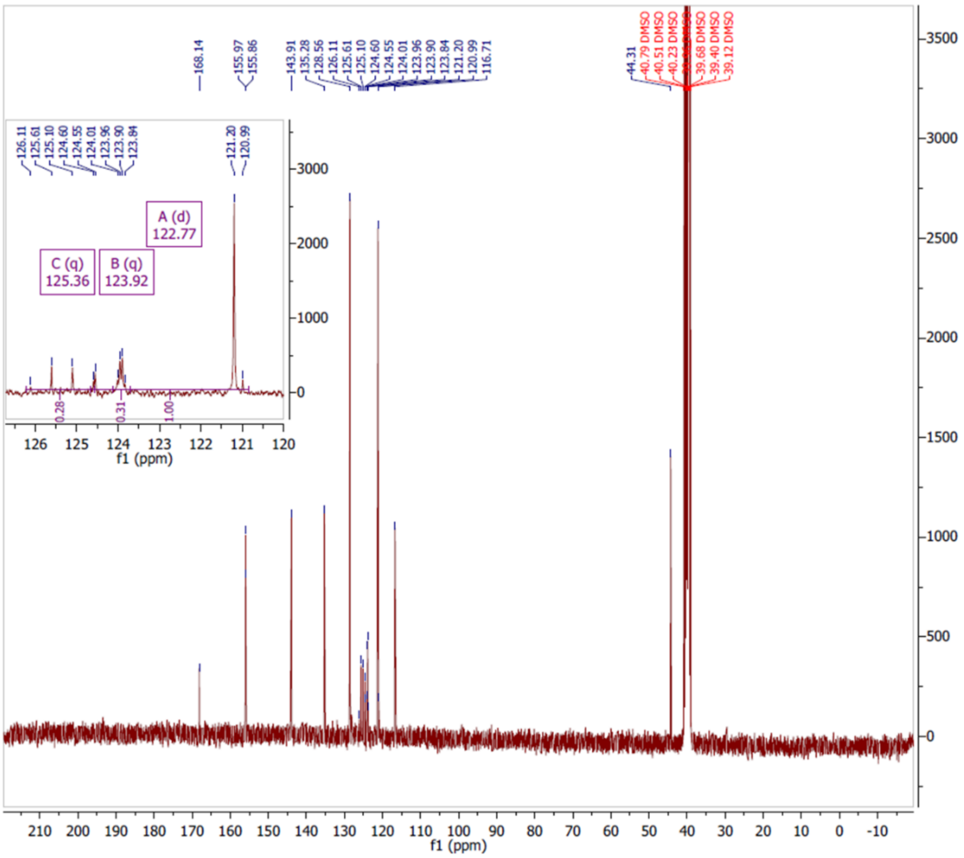

Parameter	Value
Title	tpcr11-47-3.11.fid
Origin	Bruker BioSpin GmbH
Instrument	spect
Solvent	DMSO
Temperature	293.0
Pulse Sequence	zgpg30
Experiment	1D
Probe	5 mm PABBO BB-1H/ D Z-GRD Z104275/ 0242
Number of Scans	4096
Receiver Gain	191.9
Relaxation Delay	2.0000
Pulse Width	10.0000
Acquisition Time	1.8175
Acquisition Date	2022-10-12T23:10:00
Spectrometer Frequency	75.48
Spectral Width	18028.8
Lowest Frequency	-1468.5
Nucleus	13C
Acquired Size	32768
Spectral Size	65536

^{13}C NMR (75 MHz, DMSO) δ 168.14, 155.97, 155.86, 143.91, 135.28, 128.56, 126.11, 125.61, 125.10, 124.60, 124.55, 124.01, 123.96, 123.90, 123.84, 121.20, 120.99, 116.71, 44.31.

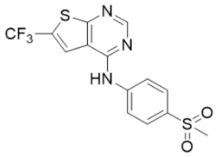

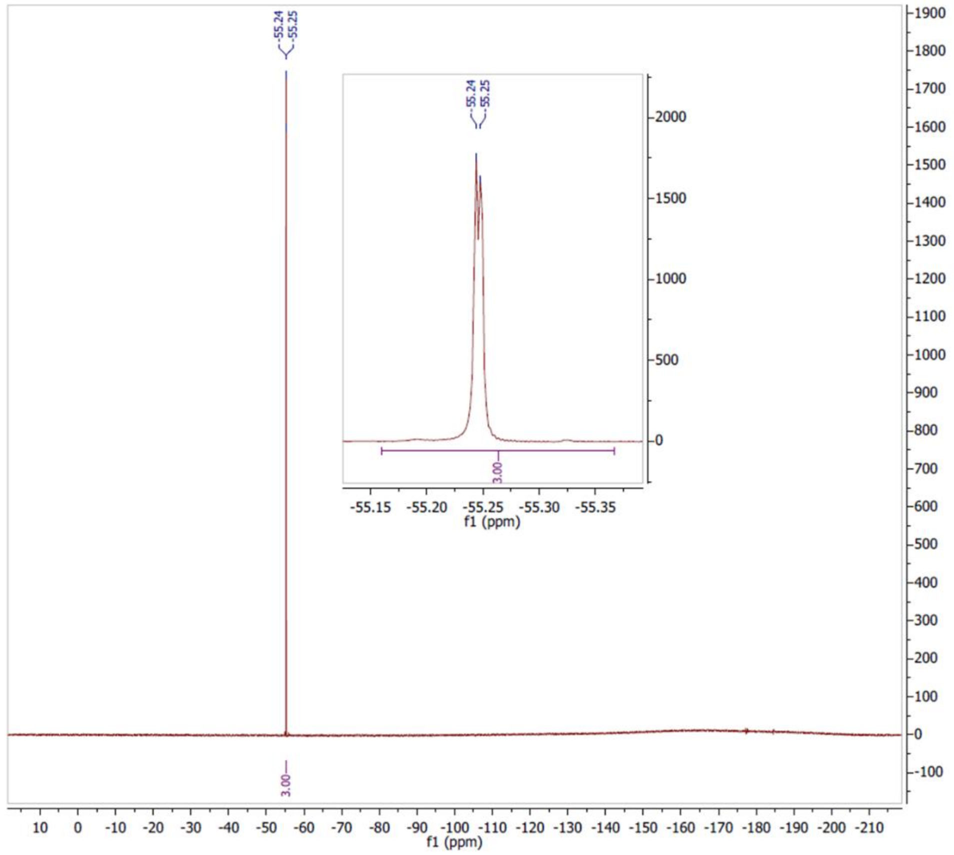

Parameter	Value
Title	GGA2-54-1.11.fid
Origin	Bruker BioSpin GmbH
Instrument	spect
Solvent	DMSO
Temperature	295.8
Pulse Sequence	zgflqn
Experiment	1D
Probe	5 mm PABBO BB-1H/ D Z-GRD Z104275/ 0242
Number of Scans	16
Receiver Gain	191.9
Relaxation Delay	1.0000
Pulse Width	15.0000
Spectrometer Frequency	282.38
Spectral Width	66964.3
Lowest Frequency	-61722.4
Nucleus	19F
Acquired Size	65536
Spectral Size	131072

^{19}F NMR (282 MHz, DMSO-d_6) δ -55.25 (d, J = 1.1 Hz).

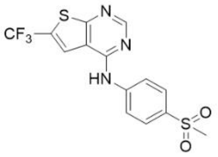

Compound **43**

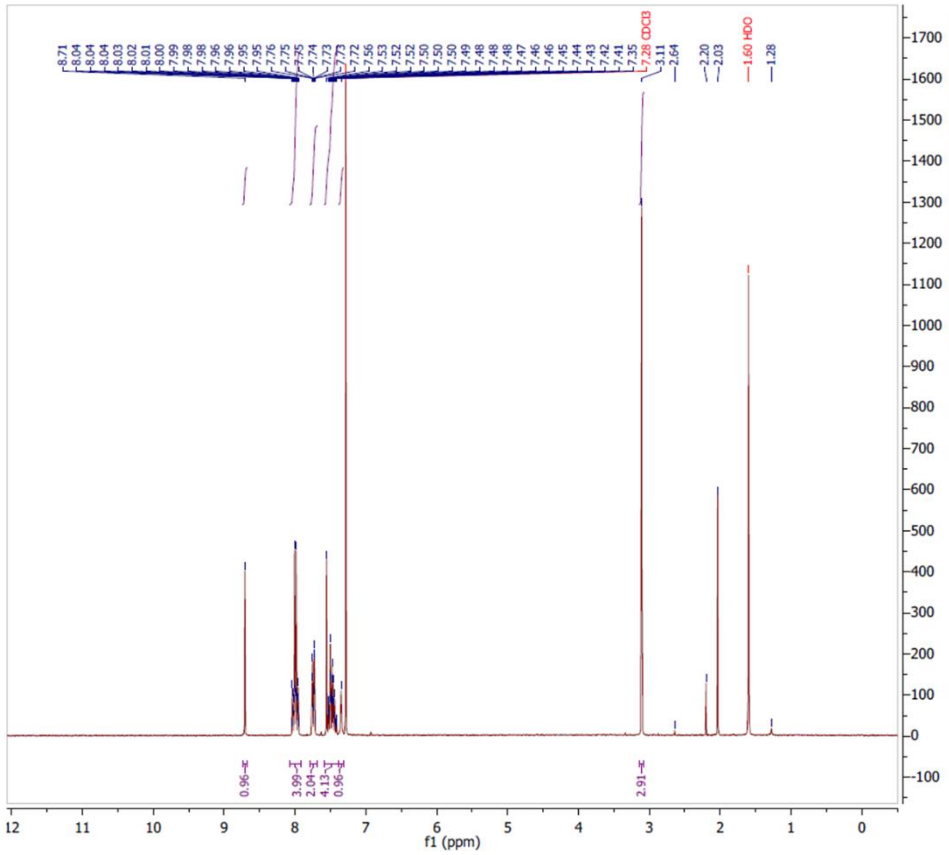

Parameter	Value
Title	Simon Edwards.20.fid
Origin	Bruker BioSpin GmbH
Instrument	spect
Solvent	CDCl3
Temperature	296.1
Pulse Sequence	zg30
Experiment	1D
Probe	5 mm PABBO BB-1H/ D Z-GRD Z104275/ 0242
Number of Scans	16
Receiver Gain	191.9
Relaxation Delay	1.0000
Pulse Width	14.0000
Spectrometer Frequency	300.13
Spectral Width	6009.6
Lowest Frequency	-1151.4
Nucleus	1H
Acquired Size	32768
Spectral Size	65536

^{1}H NMR (300 MHz, Chloroform-*d*) δ 8.71 (s, 1H), 8.07 – 7.92 (m, 4H), 7.79 – 7.69 (m, 2H), 7.59 – 7.38 (m, 4H), 7.35 (s, 1H), 3.11 (s, 3H).

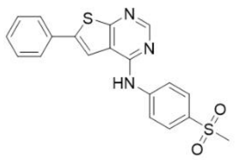

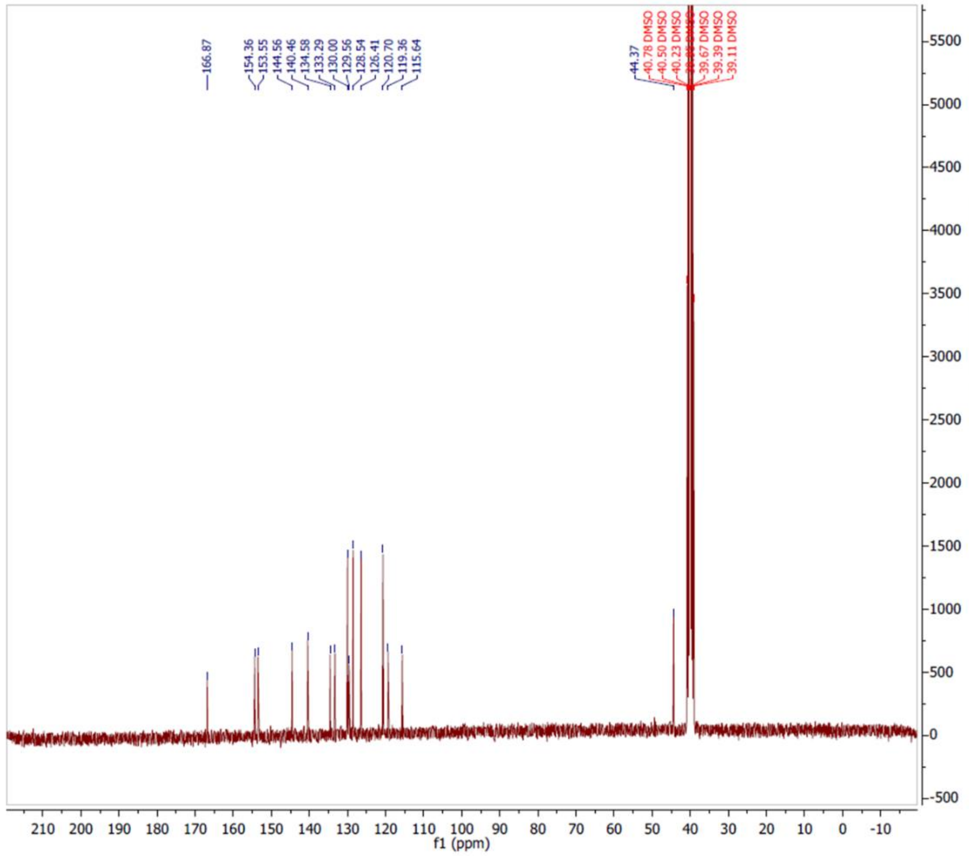

Parameter	Value
Title	tpcr11-47-2.11.fid
Origin	Bruker BioSpin GmbH
Instrument	spect
Solvent	DMSO
Temperature	291.8
Pulse Sequence	zgpg30
Experiment	1D
Probe	5 mm PABBO BB-1H/ D Z-GRD Z104275/ 0242
Number of Scans	4096
Receiver Gain	191.9
Relaxation Delay	2.0000
Pulse Width	10.0000
Acquisition Time	1.8175
Acquisition Date	2022-10-12T04:21:00
Spectrometer Frequency	75.48
Spectral Width	18028.8
Lowest Frequency	-1468.5
Nucleus	13C
Acquired Size	32768
Spectral Size	65536

^{13}C NMR (75 MHz, DMSO) δ 166.87, 154.36, 153.55, 144.56, 140.46, 134.58, 133.29, 130.00, 129.56, 128.54, 126.41, 120.70, 119.36, 115.64, 44.37.

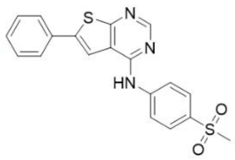

HPLC traces of compounds from Table 6

Compound **1**

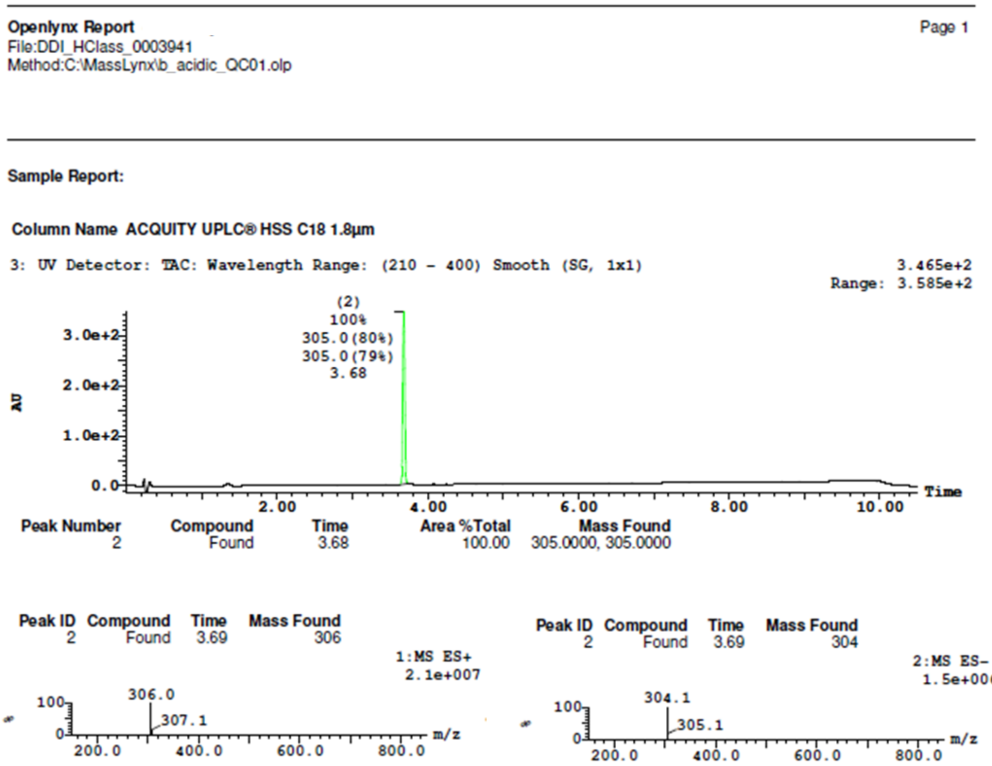

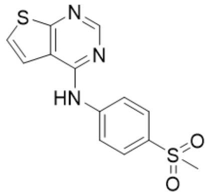

Exact Mass: 305.0

Compound **7**

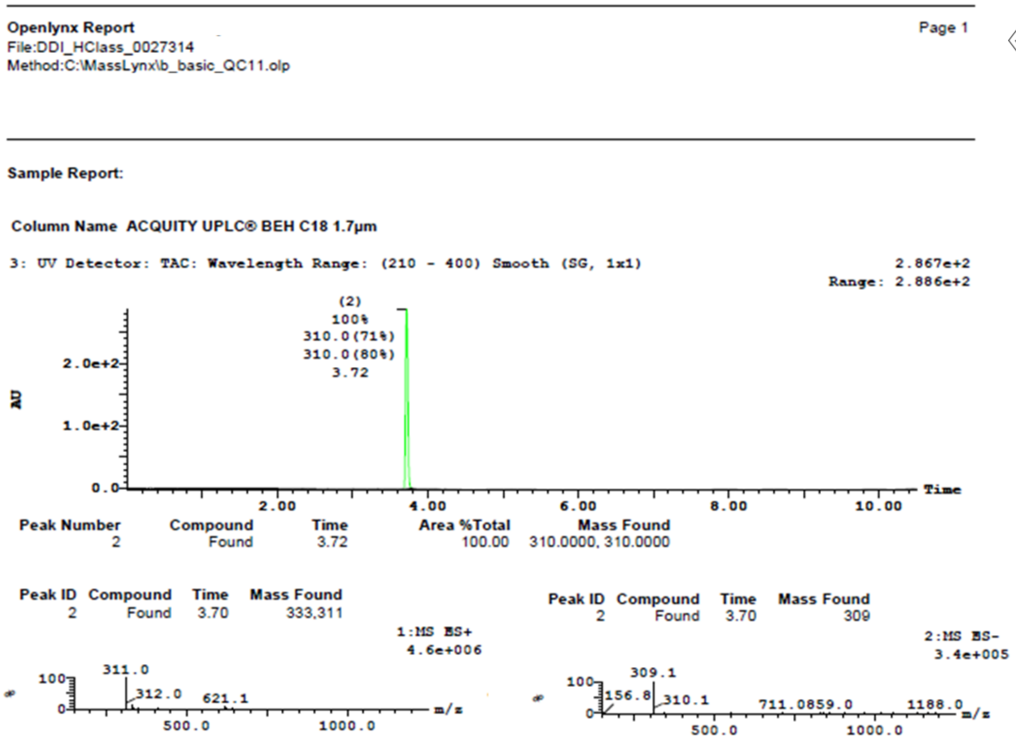

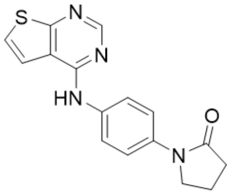

Exact Mass: 310.1

Compound **15**

Openlynx Report
File:DDI_HClass_0005444
Method:C:\MassLynx\b_basic_QC03.olp

Page 1

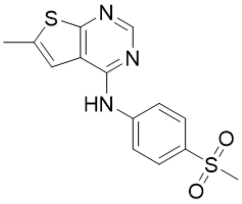

Exact Mass: 319.0

Sample Report:

Column Name ACQUITY UPLC® BEH C18 1.7µm

3: UV Detector: TAC: Wavelength Range: (210 - 400) Smooth (SG, 1x1)

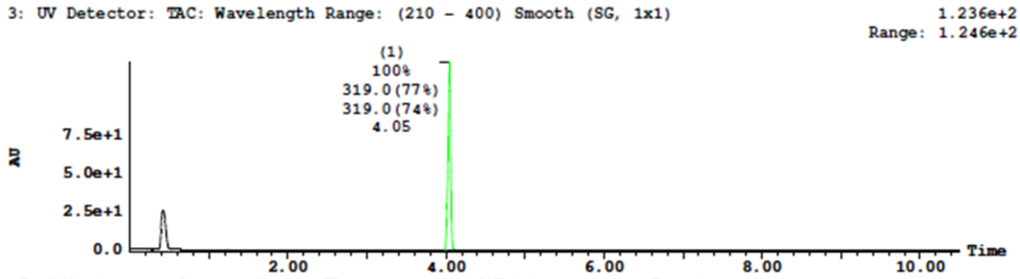

Peak Number	Compound	Time	Area %Total	Mass Found
1	Found	4.05	100.00	319.0000, 319.0000

Peak ID	Compound	Time	Mass Found
1	Found	4.04	320

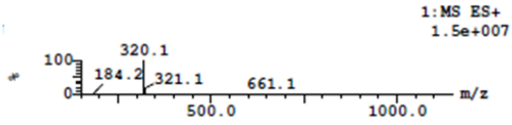

Peak ID	Compound	Time	Mass Found
1	Found	4.04	318

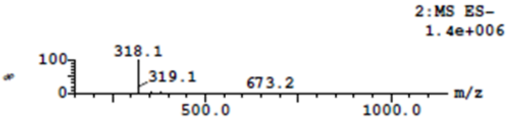

Compound **23**

Openlynx Report
File:DDI_HClass_0004608
Method:C:\MassLynx\b_basic_QC03.olp

Page 1

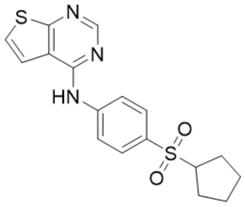

Exact Mass: 359.1

Sample Report:

Column Name ACQUITY UPLC® BEH C18 1.7µm

3: UV Detector: TAC: Wavelength Range: (210 - 400) Smooth (SG, 1x1)

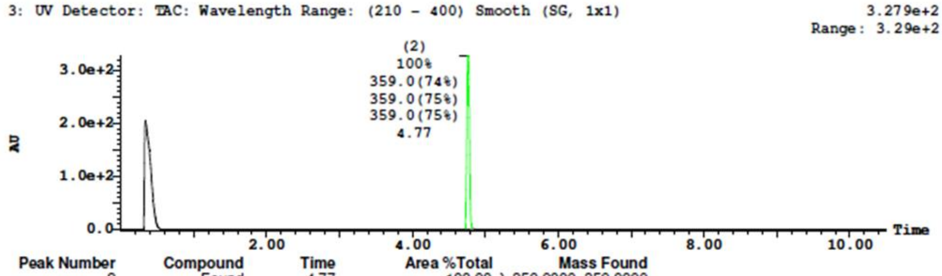

Peak Number	Compound	Time	Area %Total	Mass Found
2	Found	4.77	100.00	), 359.0000, 359.0000

Peak ID	Compound	Time	Mass Found
2	Found	4.79	382,360

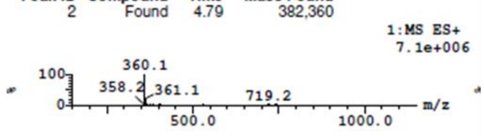

Peak ID	Compound	Time	Mass Found
2	Found	4.79	358

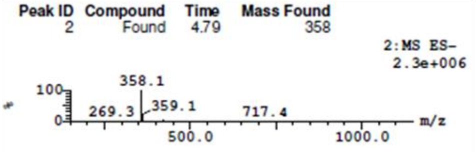

Compound **24**

Openlynx Report
File:DDI_HClass_0004593
Method:C:\MassLynx\b_basic_QC03.olp

Page 1

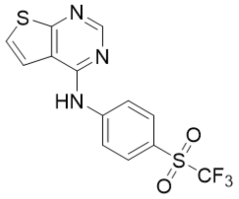

Exact Mass: 359.0

Sample Report:

Column Name ACQUITY UPLC® BEH C18 1.7µm

3: UV Detector: TAC: Wavelength Range: (210 - 400) Smooth (SG, 1x1)

2.517e+2
Range: 2.53e+2

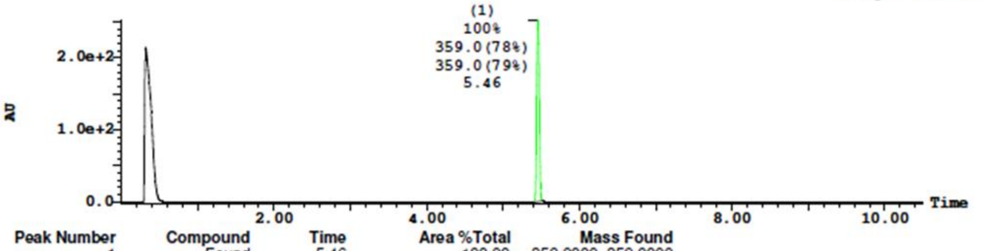

Peak Number	Compound	Time	Area %Total	Mass Found
1	Found	5.46	100.00	359.0000, 359.0000

Peak ID	Compound	Time	Mass Found
1	Found	5.45	360

1:MS ES+
1.3e+007

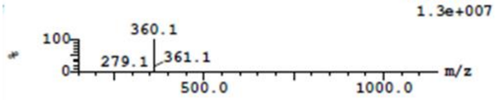

Peak ID	Compound	Time	Mass Found
1	Found	5.45	358

2:MS ES-
6.9e+006

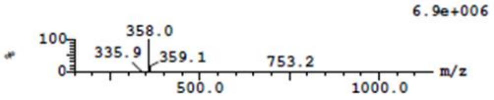

Compound **35**

Openlynx Report
File:DDI_HClass_0007535
Method:C:\MassLynx\b_basic_QC05.olp

Page 1

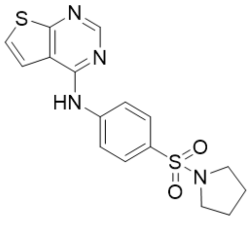

Exact Mass: 360.1

Sample Report:

Column Name ACQUITY UPLC® BEH C18 1.7µm

3: UV Detector: TAC: Wavelength Range: (210 - 400) Smooth (SG, 1x1)

6.012e+1
Range: 6.045e+1

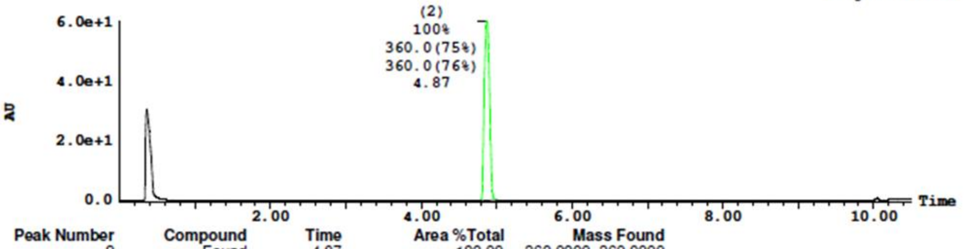

Peak Number	Compound	Time	Area %Total	Mass Found
2	Found	4.87	100.00	360.0000, 360.0000

Peak ID	Compound	Time	Mass Found
2	Found	4.86	383,361

1:MS ES+
1.3e+006

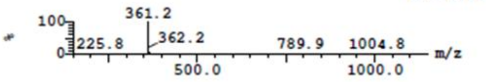

Peak ID	Compound	Time	Mass Found
2	Found	4.86	359

2:MS ES-
3.8e+005

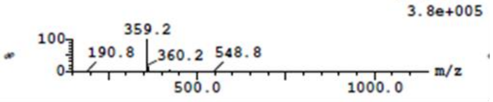

Compound **36**

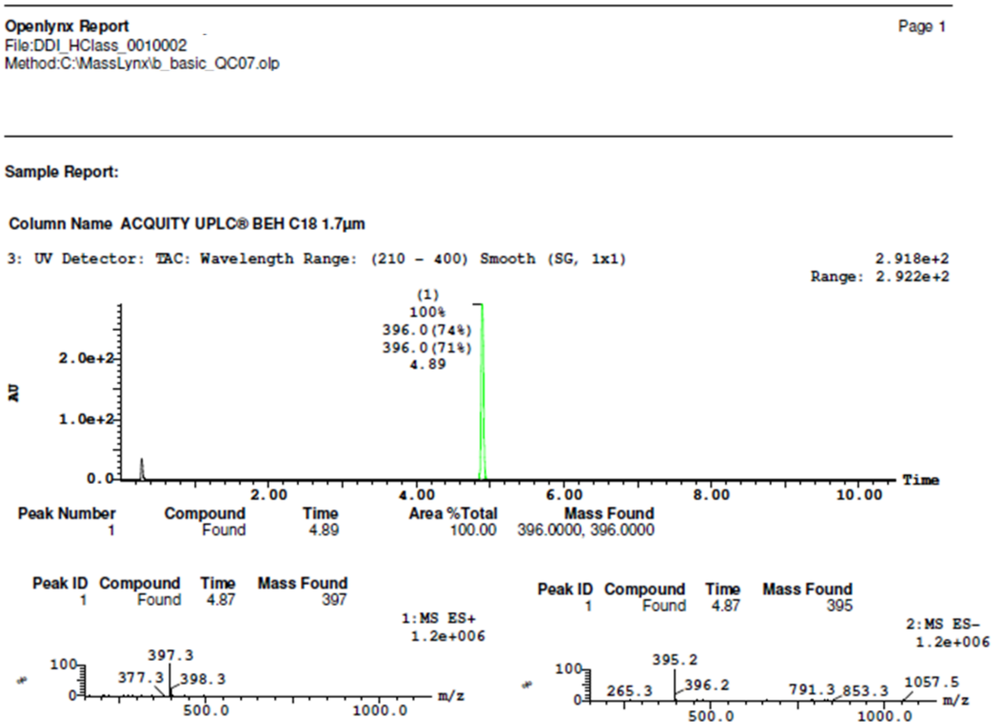

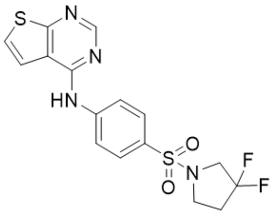

Exact Mass: 396.1

Compound **37**

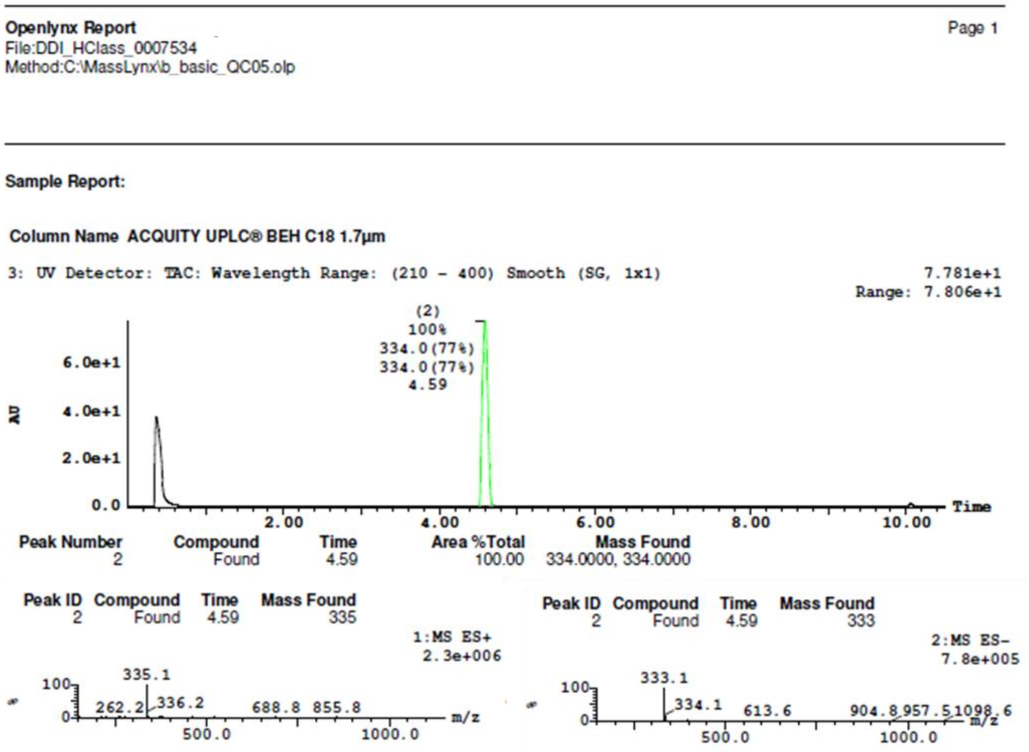

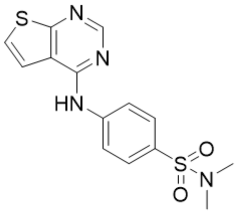

Exact Mass: 334.1

Compound **38**

Openlynx Report
File:DDI_HClass_0009940
Method:C:\MassLynx\b_basic_QC06.olp

Page 1

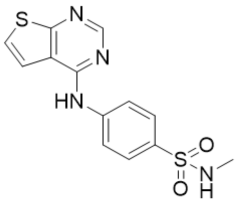

Exact Mass: 320.0

Sample Report:

Column Name ACQUITY UPLC® BEH C18 1.7µm

3: UV Detector: TAC: Wavelength Range: (210 - 400) Smooth (SG, 1x1) 2.365e+2 Range: 2.369e+2

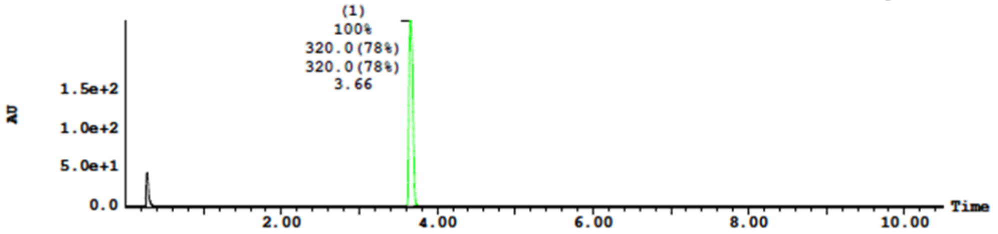

Peak Number	Compound	Time	Area %Total	Mass Found
1	Found	3.66	100.00	320.0000, 320.0000

Peak ID	Compound	Time	Mass Found
1	Found	3.64	321

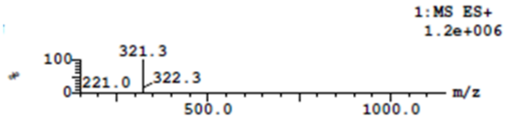

Peak ID	Compound	Time	Mass Found
1	Found	3.64	319

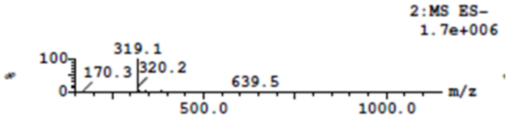

Compound **40**

Openlynx Report
File:DDI_HClass_0008985
Method:C:\MassLynx\b_acidic_QC03.olp

Page 1

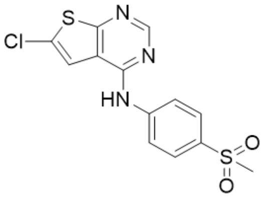

Exact Mass: 339.0

Sample Report:

Column Name ACQUITY UPLC® HSS C18 1.8µm

3: UV Detector: TAC: Wavelength Range: (230 - 400) Smooth (SG, 1x1) 1.996e+2 Range: 2.002e+2

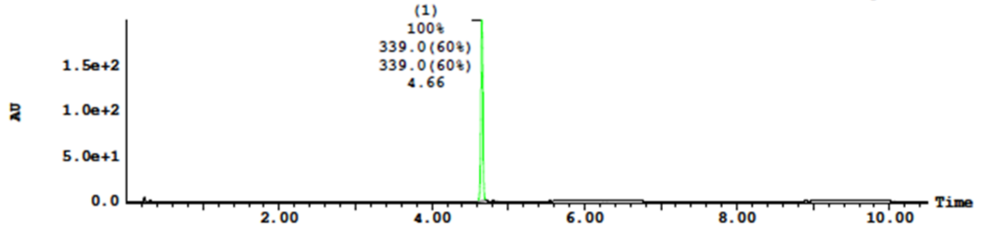

Peak Number	Compound	Time	Area %Total	Mass Found
1	Found	4.66	100.00	339.0000, 339.0000

Peak ID	Compound	Time	Mass Found
1	Found	4.66	340

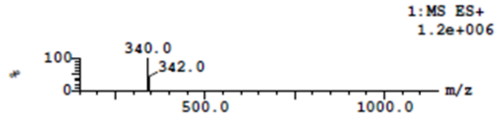

Peak ID	Compound	Time	Mass Found
1	Found	4.66	338

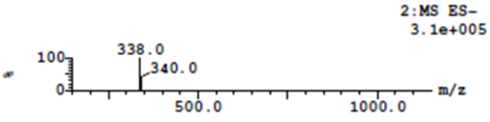

Compound **41**

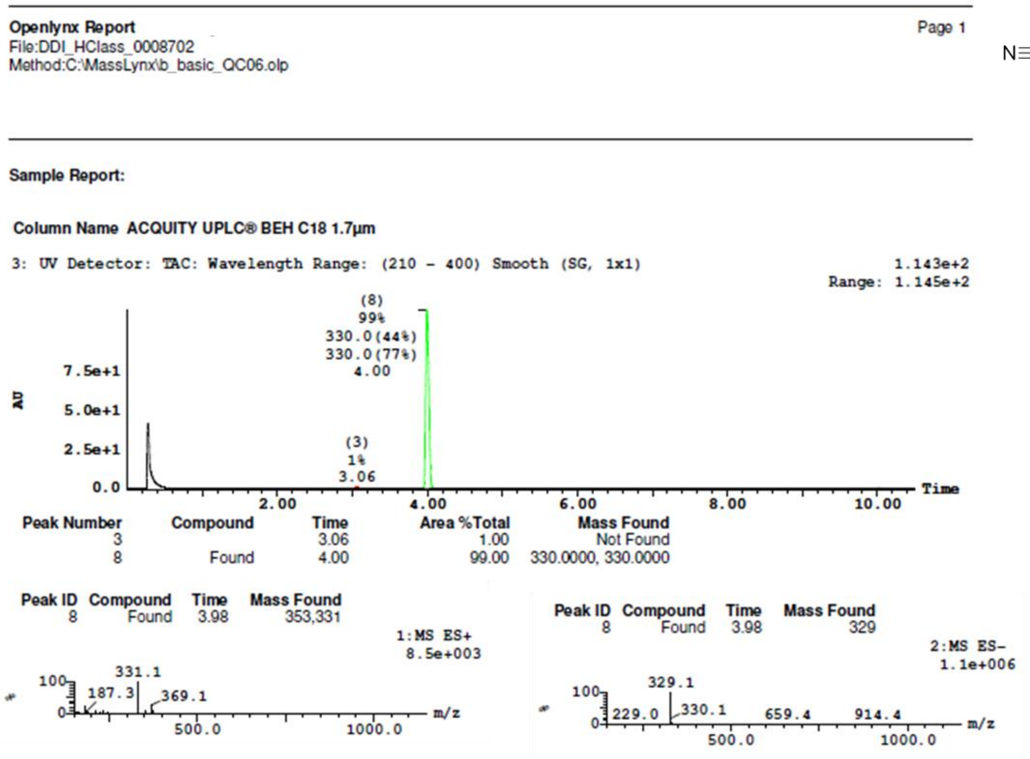
Openlynx Report
File:DDI_HClass_0008702
Method:C:\MassLynx\b_basic_QC06.olp
Page 1

Sample Report:

Column Name ACQUITY UPLC® BEH C18 1.7µm

3: UV Detector: TAC: Wavelength Range: (210 - 400) Smooth (SG, 1x1) 1.143e+2 Range: 1.145e+2

Peak Number	Compound	Time	Area %Total	Mass Found
3		3.06	1.00	Not Found
8	Found	4.00	99.00	330.0000, 330.0000

Peak ID	Compound	Time	Mass Found
8	Found	3.98	353,331

Peak ID	Compound	Time	Mass Found
8	Found	3.98	329

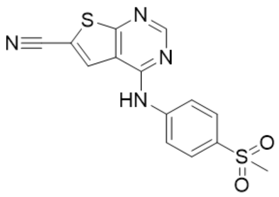

Exact Mass: 330.0

Compound **42**

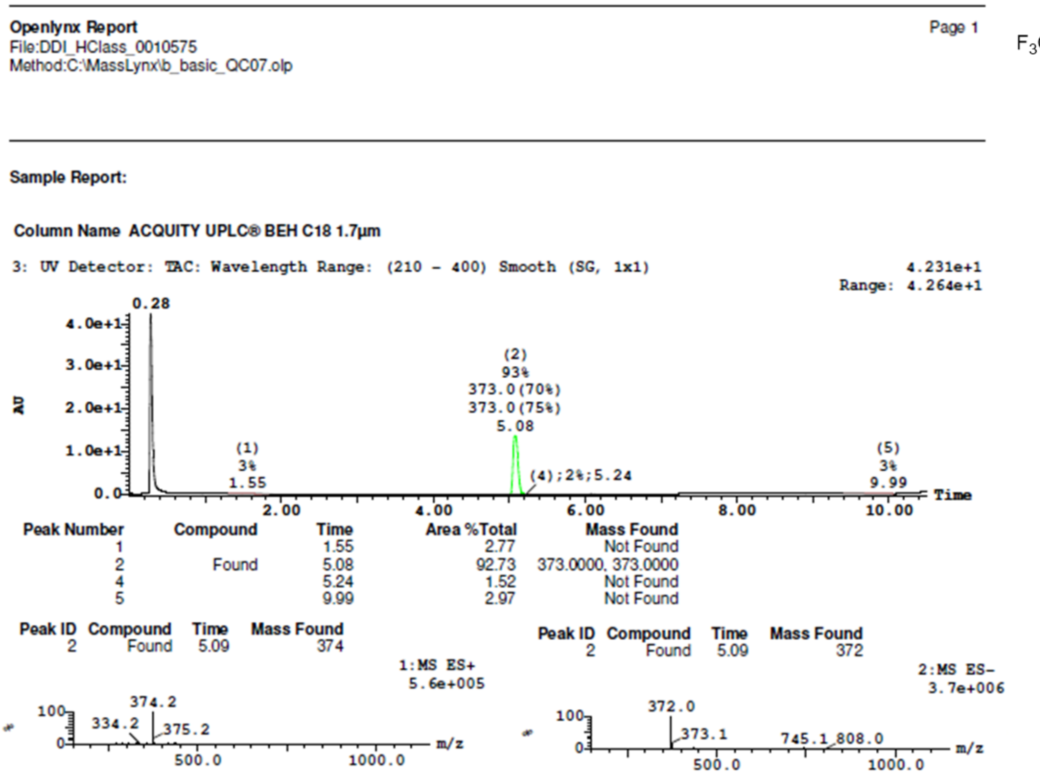
Openlynx Report
File:DDI_HClass_0010575
Method:C:\MassLynx\b_basic_QC07.olp
Page 1

Sample Report:

Column Name ACQUITY UPLC® BEH C18 1.7µm

3: UV Detector: TAC: Wavelength Range: (210 - 400) Smooth (SG, 1x1) 4.231e+1 Range: 4.264e+1

Peak Number	Compound	Time	Area %Total	Mass Found
1		1.55	2.77	Not Found
2	Found	5.08	92.73	373.0000, 373.0000
4		5.24	1.52	Not Found
5		9.99	2.97	Not Found

Peak ID	Compound	Time	Mass Found
2	Found	5.09	374

Peak ID	Compound	Time	Mass Found
2	Found	5.09	372

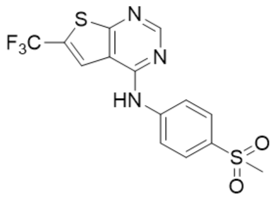

Exact Mass: 373.0

Compound **43**

Openlynx Report
File:DDI_HClass_0004693
Method:C:\MassLynx\b_basic_QC03.olp

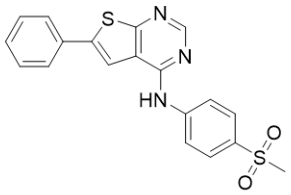

Exact Mass: 381.1

Sample Report:

Column Name ACQUITY UPLC® BEH C18 1.7µm

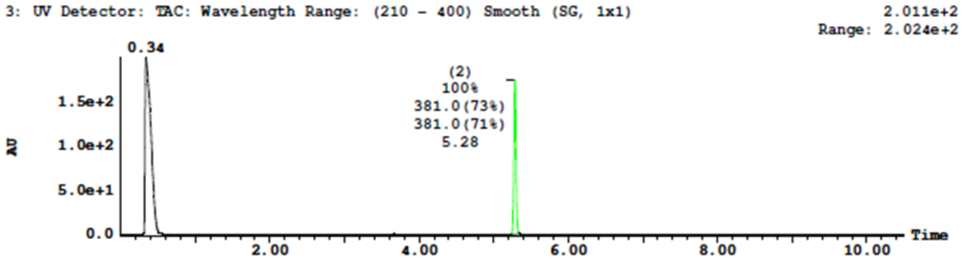

Peak Number	Compound	Time	Area %Total	Mass Found
2	Found	5.28	100.00	381.0000, 381.0000

Peak ID	Compound	Time	Mass Found
2	Found	5.28	382

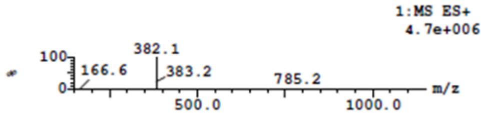

Peak ID	Compound	Time	Mass Found
2	Found	5.28	380

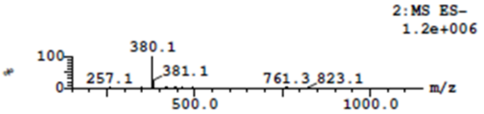

Supplement: Supplementary file 2 — jm2c01693_si_002.pdf [file jm2c01693_si_002.pdf]
